# MicroRNA-7: a versatile player and core target in brain disorders

**DOI:** 10.1186/s12967-026-08313-9

**Published:** 2026-05-27

**Authors:** Jianhua Deng, Daosheng Li, Zhiqi Li, Yuanming Pan, Jiajun Xiong

**Affiliations:** 1 Department of Oncology, Jiujiang City Key Laboratory of Cell Therapy, Jiujiang No.1 People’s Hospital, No.48 Taling South Road, Xunyang District, Jiujiang, Jiangxi Province 332001 China; 2 Cancer Research Center, Beijing Chest Hospital, Capital Medical University/Beijing. Tuberculosis and Thoracic Tumor Research Institute, No.9 Beiguan Street, Tongzhou District, Beijing, 101149 China; 3 Department of Cardiothoracic Surgery, Jiujiang City Key Laboratory of Cell Therapy, Jiujiang No.1 People’s Hospital, No. 48 Taling South Road, Xunyang District, Jiujiang, Jiangxi Province 332001 China

**Keywords:** miR-7, Brain diseases, Epigenetic regulation, CeRNA network, Nano-delivery system

## Abstract

**Objective:**

This article provides a systematic review of the biological characteristics, regulatory mechanisms, and roles of MicroRNA-7 (miR-7) in the nervous system, as well as explores its clinical translation potential and challenges.

**Methods:**

A review analysis integrating the progress of basic and clinical research related to miR-7.

**Results:**

Characteristics and Regulation: miR-7 is highly expressed in mammalian brain tissues, with its function finely regulated by ceRNA networks (such as ciRS-7 and lncRNA SNHG1) and epigenetic modifications, forming a multi-layer dynamic regulatory system. Mechanisms of Action: It plays multi-faceted intervention roles in protein aggregation, neuroinflammation, mitochondrial dysfunction, and tumor progression by targeting key factors such as α-synuclein, NLRP3 inflammasome, EGFR/PI3K/AKT/mTOR pathway, and mitochondrial-related proteins (e.g. VDAC1). Role in Disease: It has significant pathophysiological implications in diseases such as Parkinson’s disease, Alzheimer’s disease, ischemic stroke, cerebral hemorrhage, and glioblastoma. Translational Potential: Changes in miR-7 expression in bodily fluids (blood, cerebrospinal fluid) and extracellular vesicles demonstrate diagnostic and prognostic potential; delivery systems based on nanomedicine (e.g. liposomes, graphene oxide, AAV vectors) and their combination therapy strategies (e.g. in conjunction with chemotherapy and immunotherapy) enhance its brain targeting and therapeutic efficiency.

**Conclusion:**

miR-7 is a key regulatory molecule in brain diseases, with significant value for basic research and clinical translation. Future advancements should leverage cutting-edge technologies such as single-cell sequencing, spatial transcriptomics, and intelligent responsive nanocarriers to deepen the understanding of its regulatory networks and address challenges such as mechanism complexity, delivery system targeting, and the lack of clinical translation standards, to accelerate its transition from basic research to clinical application.

## Graphical Abstract

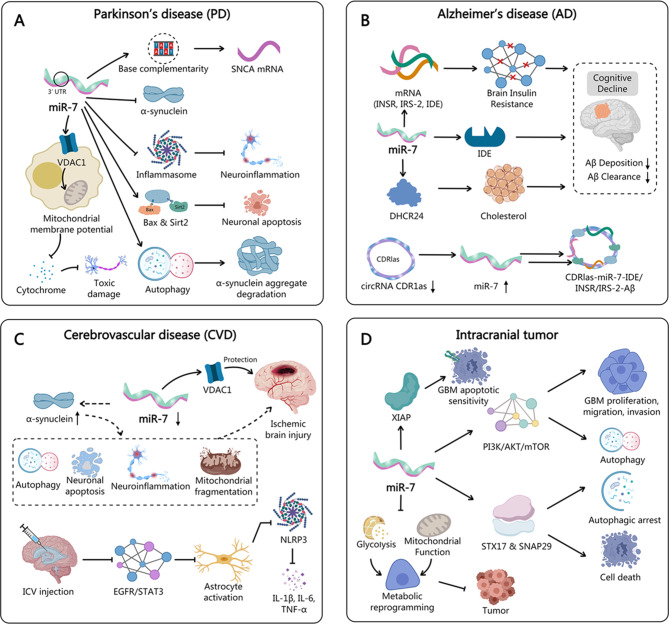

## Introduction

### Overview of the biological characteristics and functions of miR-7

#### Discovery and structural characteristics of miR-7

MicroRNAs (miRNAs) are a class of endogenous non-coding small RNA molecules approximately 22 nucleotides in length. They regulate gene expression at the post-transcriptional level by specifically binding to the 3’ untranslated region (3’-UTR) of target gene mRNAs, thereby participating in the regulation of various physiological and pathological processes such as cell proliferation, differentiation, and apoptosis [1]. As an important member of the miRNA family, miR-7 was first discovered in *Caenorhabditis elegans* in 2001 and subsequently identified in various mammals, including humans. In mammals, miR-7 is primarily encoded by three genes: MIR7-1 (located on chromosome 9), MIR7-2 (located on chromosome 15), and MIR7-3 (located on chromosome 19). These genes transcribe to form precursor structures (pre-miR-7) with different sequences, which are ultimately processed into mature miR-7 molecules with identical sequences [1]. This multi-gene encoding characteristic may confer flexibility and robustness to miR-7 in its expression regulation across different tissues and developmental stages.

miR-7 is highly abundant in the nervous system, particularly in the mammalian brain, and is predominantly expressed in neurons, which is closely related to its critical role in brain development and functional maintenance [1, 2]. For instance, during zebrafish embryonic development, the homolog of miR-7, miR-7a, is specifically expressed in the forebrain region, particularly during the development of dopaminergic neurons (DA neurons), suggesting its conserved function in neural cell fate determination [3]. In the human brain, miR-7 is not only expressed in developing brain tissues but also remains highly expressed in multiple regions of the adult brain, such as the cortex, hippocampus, and substantia nigra, where it participates in maintaining normal physiological functions of neurons and synaptic plasticity [1, 2].

Structurally, the mature miR-7 molecule exhibits typical miRNA secondary structural features, with its seed sequence (usually nucleotides 2–8 at the 5’ end) serving as the key region for recognizing and binding to target gene mRNAs. Through systematic prediction and experimental validation of miR-7 target genes, it has been found to bind to numerous mRNAs involved in cellular signal transduction, metabolic regulation, inflammatory responses, and neurodegeneration, thereby widely participating in the regulation of cellular life activities [1, 4]. For example, the seed sequence of miR-7 specifically recognizes the 3‘UTR of the α-synuclein (SNCA) gene mRNA, inhibiting its translation—an interaction that plays a crucial role in the pathophysiology of Parkinson’s disease (PD) [5–7]. Additionally, miR-7 can target genes such as the insulin receptor (INSR), insulin receptor substrate 2 (IRS-2), and insulin-degrading enzyme (IDE), participating in the regulation of insulin signaling pathways in the brain and closely associated with the development of neurodegenerative diseases like Alzheimer’s disease (AD) [8].

The expression regulation of miR-7 is precisely controlled by multiple mechanisms, including transcriptional and post-transcriptional regulation. At the transcriptional level, the expression of miR-7 host genes (such as HNRNPK, the host gene of MIR7-1) is regulated by various transcription factors and epigenetic modifications. For instance, insulin and liver X receptor (LXR) activators can promote the transcription of the HNRNPK gene, thereby upregulating the expression of miR-7–1 [8]. At the post-transcriptional level, the maturation process of miR-7 is regulated by various RNA-binding proteins (such as HuR) and non-coding RNAs (such as ciRS-7 and lncRNA Cyrano) [2, 9, 10]. These complex regulatory mechanisms ensure the precise expression of miR-7 under different physiological and pathological conditions to meet cellular functional demands.

#### Epigenetic regulation mechanism of miR-7

Epigenetic regulation refers to the heritable control of gene expression without altering the DNA sequence, achieved through mechanisms such as DNA methylation, histone modification, and non-coding RNA regulation [11–13]. As an important epigenetic regulator, miR-7‘s own expression is tightly controlled by epigenetic mechanisms. Meanwhile, miR-7 also participates in the epigenetic regulatory network by modulating the expression of target genes, thereby influencing cellular physiological and pathological processes [14–16].

The transcription of the miR-7 gene is regulated by epigenetic mechanisms such as DNA methylation and histone modifications. Although direct studies on the methylation status of the miR-7 promoter region are relatively limited, existing research indicates that in some tumor cells, the low expression of miR-7 is associated with hypermethylation of the promoter region, suggesting that DNA methylation may be one of the important mechanisms regulating miR-7 expression [4]. Additionally, histone modifications such as histone acetylation and methylation may also be involved in the transcriptional regulation of miR-7. For example, the transcription factor SREBP2 can bind to the promoter region of the miR-7 gene to regulate its transcription, and the activity of SREBP2 itself is influenced by cholesterol metabolism status. This metabolic-epigenetic cross-regulation may play a role in miR-7-mediated cholesterol homeostasis maintenance [17].

The maturation process of miR-7 is also finely regulated at the epigenetic level. The primary transcript of miR-7 (pri-miR-7) requires cleavage and processing by enzymes such as Drosha and Dicer to generate the mature miRNA molecule. This process is regulated by various RNA-binding proteins; for example, the HuR protein can bind to the conserved terminal loop structure of pri-miR-7–1, inhibiting its cleavage and processing, thereby negatively regulating miR-7 maturation [9]. In contrast, the natural product quercetin can promote miR-7 maturation by inhibiting the interaction between HuR and pri-miR-7–1, subsequently downregulating α-synuclein expression, which provides a potential epigenetic intervention target for the treatment of Parkinson’s disease (PD) [9].

More importantly, as an executor of epigenetic regulation, miR-7 indirectly influences the epigenetic states of other genes by targeting enzymes or proteins associated with epigenetic regulation. For instance, sirtuin 2 (Sirt2), a deacetylase involved in histone deacetylation and non-histone deacetylation modifications, plays roles in cell cycle regulation, metabolism, and neurodegenerative diseases. Studies have found that miR-7 can directly target the 3‘UTR of Sirt2, inhibiting its expression and thereby regulating the acetylation levels of downstream target genes and the process of apoptosis [18]. In an MPP+-induced PD cell model, miR-7 reduced neuronal apoptosis by inhibiting Sirt2 expression, exerting a neuroprotective effect [18]. Additionally, miR-7 can participate in a broader epigenetic regulatory network by targeting other epigenetic regulators, such as DNA methyltransferases or histone-modifying enzymes, though research in this area remains preliminary and requires further in-depth exploration.

The epigenetic regulation of miR-7 is also reflected in its interaction with other non-coding RNAs, forming a complex epigenetic regulatory network. For example, circRNA CDR1as (ciRS-7), a circular RNA highly expressed in neurons, contains approximately 70 binding sites for miR-7 and can act as an endogenous miR-7 “sponge,” competitively binding miR-7 and inhibiting its regulatory effects on target genes [2, 10, 19]. Under physiological conditions, ciRS-7 regulates glutamatergic synaptic transmission and neuronal connectivity by buffering miR-7 activity, thereby maintaining normal brain function [2]. In pathological states, such as cerebral ischemia, the downregulation of ciRS-7 expression leads to abnormal elevation of miR-7 activity, which exacerbates neuronal damage by targeting genes like α-synuclein [20]. Additionally, lncRNA Cyrano can trigger the degradation of miR-7 through extensive complementary pairing, indirectly regulating ciRS-7 expression levels. This forms a complex non-coding RNA regulatory network composed of Cyrano-miR-7-ciRS-7, which plays a crucial role in maintaining neuronal function and the development of brain diseases [10].

#### Molecular regulatory network of miR-7

miR-7, as a key regulatory non-coding RNA, establishes a complex and sophisticated molecular regulatory network through direct binding with target gene mRNAs and interactions with other non-coding RNAs, extensively participating in both physiological and pathological cellular processes [10, 21, 22]. This network not only includes the direct post-transcriptional regulation of downstream target genes by miR-7 [10, 22], but also involves mutual regulation among non-coding RNAs centered on the competitive endogenous RNA (ceRNA) mechanism [20, 21, 23], enabling miR-7 to influence cellular functions in a multidimensional and multi-level manner [10, 22, 24].

**Fig. 1:**
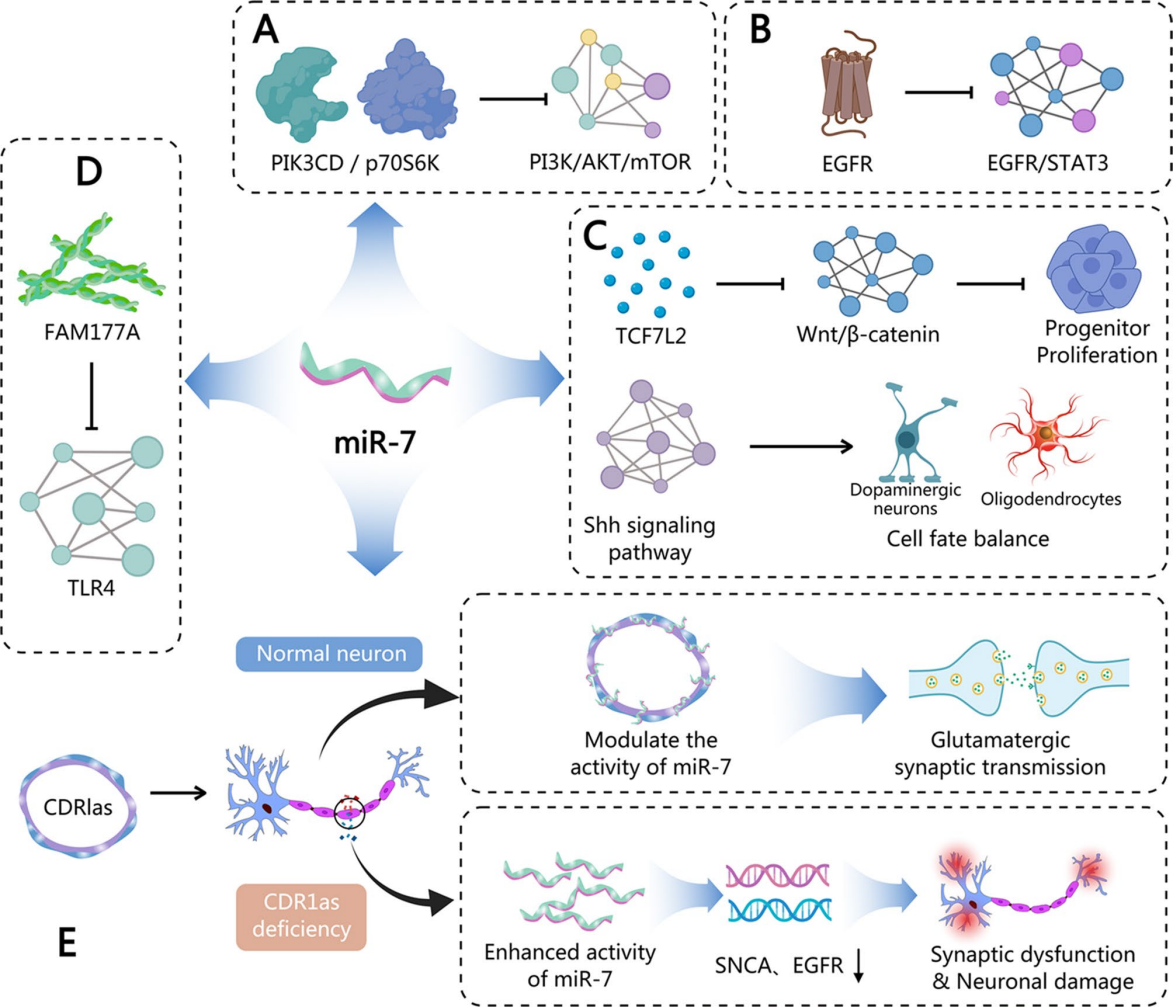
Schematic overview of miR-7–mediated regulatory networks in neuronal function and diseases. (**A**) miR-7 targets key molecules of the PI3K/AKT/mTOR pathway, PIK3CD and the p70S6K signaling pathway, inhibiting their expression and negatively regulating the activity of this pathway. (**B**) miR-7 suppresses the epidermal growth factor receptor (EGFR), inhibiting the EGFR/STAT3 signaling pathway. (**C**) miR-7 modulates TCF7L2 and Wnt/β-catenin signaling, influencing progenitor proliferation, shh signaling, and the balance between dopaminergic neurons and oligodendrocytes, thereby regulating cell fate. (**D**) miR-7 interacts with sequence similarity 177 member a (FAM177A), negatively regulating the TLR4 signaling pathway. (**E**) In normal neurons, ciRS-7 modulates miR-7 activity, affecting glutamatergic synaptic transmission. Enhanced miR-7 activity leads to downregulation of SNCA and EGFR, contributing to synaptic dysfunction and neuronal damage

miR-7 achieves precise regulation of the cellular signaling network by directly targeting components of multiple key signaling pathways. Among them, the PI3K/AKT/mTOR signaling pathway is one of the important targets regulated by miR-7. Studies have found that miR-7 can target key molecules of the PI3K/AKT/mTOR pathway, such as the PI3K catalytic subunit δ (PIK3CD) and p70S6K, inhibiting their expression and thereby negatively regulating the activity of this pathway (Fig. 1A) [25, 26]. In colorectal cancer cells, miR-7 inhibits the activation of the PI3K/AKT/mTOR pathway by targeting TYRO3, thereby suppressing the proliferation, migration, and invasion of cancer cells [26]. In glioblastoma (GBM), miR-7 similarly inhibits the activity of the PI3K/AKT/mTOR signaling pathway to induce autophagy initiation, while also targeting SNARE proteins such as STX17 and SNAP29 to block the late stages of autophagy, leading to impaired autophagic flux and thereby inhibiting GBM progression [27]. Additionally, miR-7 can target the epidermal growth factor receptor (EGFR) and inhibit the EGFR/STAT3 signaling pathway (Fig. 1B). In an intracerebral hemorrhage (ICH) model, miR-7 directly binds to the 3‘UTR of EGFR, inhibiting its expression and the phosphorylation of downstream STAT3, thereby alleviating blood-brain barrier disruption, cerebral edema, and neuroinflammation, and exerting neuroprotective effects [28].

The Wnt/β-catenin and Shh signaling pathways play important roles in neural development and neurodegenerative diseases. The balanced regulation of these two pathways by miR-7 is a key mechanism through which it participates in neural cell fate determination. In zebrafish embryos, miR-7 negatively regulates the Wnt/β-catenin signaling pathway by targeting TCF7L2, an effector of the Wnt pathway, thereby suppressing the excessive proliferation of dopaminergic progenitor cells (Fig. 1C) [3]. At the same time, miR-7 positively regulates the Shh signaling pathway to control the balance of oligodendrocyte and dopaminergic neuron cell fates (Fig. 1C) [3]. This dual regulatory effect on the Wnt and Shh pathways gives miR-7 potential application value in cell replacement therapy for neurodegenerative diseases such as Parkinson’s disease [3, 29].

miR-7 is also widely involved in the regulation of inflammation-related signaling pathways, particularly playing a crucial regulatory role in neuroinflammatory responses. The Toll-like receptor 4 (TLR4) signaling pathway is a key pathway mediating innate immunity and inflammatory responses. miR-7 can negatively regulate the TLR4 signaling pathway by targeting family with sequence similarity 177 member A (FAM177A) (Fig. 1D). In LPS-activated bone marrow-derived macrophages, the expression of miR-7 is upregulated, which suppresses TLR4-mediated MyD88-dependent and independent signal transduction by inhibiting FAM177A expression, thereby reducing the production of pro-inflammatory cytokines such as IL-1β, IL-6, and TNF-α [30]. Additionally, miR-7 can influence neuroinflammation by modulating the activity of the NLRP3 inflammasome. In PD models, miR-7 directly targets the 3‘UTR of NLRP3, inhibiting its expression, which reduces microglial activation and the release of pro-inflammatory factors like IL-1β, thereby alleviating damage to dopaminergic neurons [31, 32]. In ICH models, miR-7 mitigates intracerebral inflammatory responses and cerebral edema by inhibiting NLRP3 inflammasome activation, improving neurological functional outcomes [33].

The ceRNA mechanism is a recently discovered important mode of gene expression regulation, referring to RNA molecules such as mRNA, lncRNA, and circRNA competitively binding to shared miRNAs, thereby mutually regulating their respective expression levels. miR-7, as a highly expressed and functionally significant miRNA in the brain, participates in multiple ceRNA regulatory networks and forms complex interactions with various non-coding RNAs. ciRS-7 is one of the most extensively studied ceRNAs associated with miR-7. ciRS-7 contains numerous miR-7 binding sites and can efficiently sponge miR-7, thereby regulating the expression of miR-7 target genes [2, 19]. In normal neurons, ciRS-7 buffers miR-7 activity, ensuring normal glutamatergic synaptic transmission and neuronal network connectivity (Fig. 1E) [2]. When ciRS-7 is deficient, miR-7 activity increases, leading to downregulation of its target genes such as SNCA and EGFR, causing synaptic dysfunction and neuronal damage (Fig. 1E) [2, 19]. In cerebral ischemia models, downregulation of ciRS-7 expression reduces the inhibitory effect of miR-7, increases α-synuclein expression, and promotes neuronal death after ischemia [20]. Additionally, through its interaction with miR-7, ciRS-7 can influence the expression of other neurodegenerative disease-related proteins, such as amyloid-beta (Aβ) metabolism in Alzheimer’s disease (AD) [34, 35].

LncRNA also plays an important role in the ceRNA regulatory network of miR-7. For example, lncRNA SNHG1 is upregulated in brain specimens of PD patients and MPP+-treated SH-SY5Y cells. SNHG1 can act as a ceRNA, competitively binding to miR-7, thereby relieving miR-7‘s inhibition of NLRP3, leading to the activation of the NLRP3 inflammasome and exacerbation of neuroinflammation [31]. In an MPTP-induced PD mouse model, knocking down SNHG1 can inhibit microglial activation and NLRP3 inflammasome activation by upregulating miR-7 expression, alleviating the loss of dopaminergic neurons [31]. Another example is lncRNA HMMR-AS1, which is highly expressed in glioblastoma tissues and cells. HMMR-AS1 upregulates its target gene CDK4 by sponging miR-7, thereby promoting the proliferation, migration, and invasion of glioma cells [36]. The anesthetic sevoflurane can inhibit HMMR-AS1 expression, relieving its sponging effect on miR-7, subsequently suppressing CDK4 expression and exerting anti-glioma effects [36]. Additionally, in rheumatoid arthritis (RA), lncRNA ciRS-7 (which shares the same name as ciRS-7, but it may refer to a different transcript or involve naming confusion, requiring careful distinction) maintains the tumor-like biological characteristics of fibroblast-like synoviocytes (FLS) by inhibiting miR-7 expression, promoting the pathological progression of RA [37].

In addition to circRNA and lncRNA, some mRNAs can also function as ceRNAs to participate in the regulatory network of miR-7. For example, in the retinal detachment (RD) model, the mRNA of α-synuclein can bind to miR-7, thereby influencing miR-7‘s regulatory effects on other target genes [38]. Additionally, the mRNA of HNRNPK, the host gene of miR-7, may also bind to miR-7 or other miRNAs through its 3‘UTR or other regions, participating in the ceRNA regulatory network. However, research in this area remains limited and requires further exploration.

The molecular regulatory network of miR-7 also involves mutual regulation among miRNAs. For example, miR-671 can cleave ciRS-7, affecting its sponge function on miR-7 and thereby indirectly regulating miR-7 activity [10]. In the absence of Cyrano, excessive miR-7 leads to the degradation of ciRS-7 in the neuronal cytoplasm, partially through enhancing miR-671-mediated cleavage of ciRS-7 [10]. This complex interplay among miRNAs and circRNAs further increases the intricacy and precision of the miR-7 regulatory network.

### Association of miR-7 with brain diseases

miR-7, as a highly abundant miRNA in the brain, plays a central role in maintaining normal neuronal physiological functions and regulating neural circuit homeostasis. Dysregulation of its expression and function is closely associated with the development and progression of various brain diseases. Numerous studies have shown that miR-7 expression levels are significantly altered in neurodegenerative diseases, cerebrovascular diseases, brain tumors, and other brain disorders. By regulating its target gene networks, miR-7 participates in the pathophysiological processes of these diseases and has emerged as a potential diagnostic biomarker and therapeutic target.

#### Neurodegenerative diseases

Parkinson’s disease (PD) is the neurodegenerative disorder most extensively studied in association with miR-7. In the substantia nigra of PD patients, miR-7 expression is significantly reduced, and this reduction is closely linked to the abnormal aggregation of α-synuclein and the loss of dopaminergic neurons [5, 39]. miR-7 directly targets the 3‘UTR of the SNCA gene, inhibiting the translation of α-synuclein and thereby reducing the formation of its monomers and aggregates [5, 6]. In α-synuclein preformed fibril (PFF)-induced PD mouse models, overexpression of miR-7 significantly lowers α-synuclein levels in the striatum, reduces the accumulation of phosphorylated α-synuclein in striatal and nigral dopaminergic neurons, thereby alleviating neurodegeneration in the nigrostriatal pathway and improving behavioral outcomes [6].

**Fig. 2:**
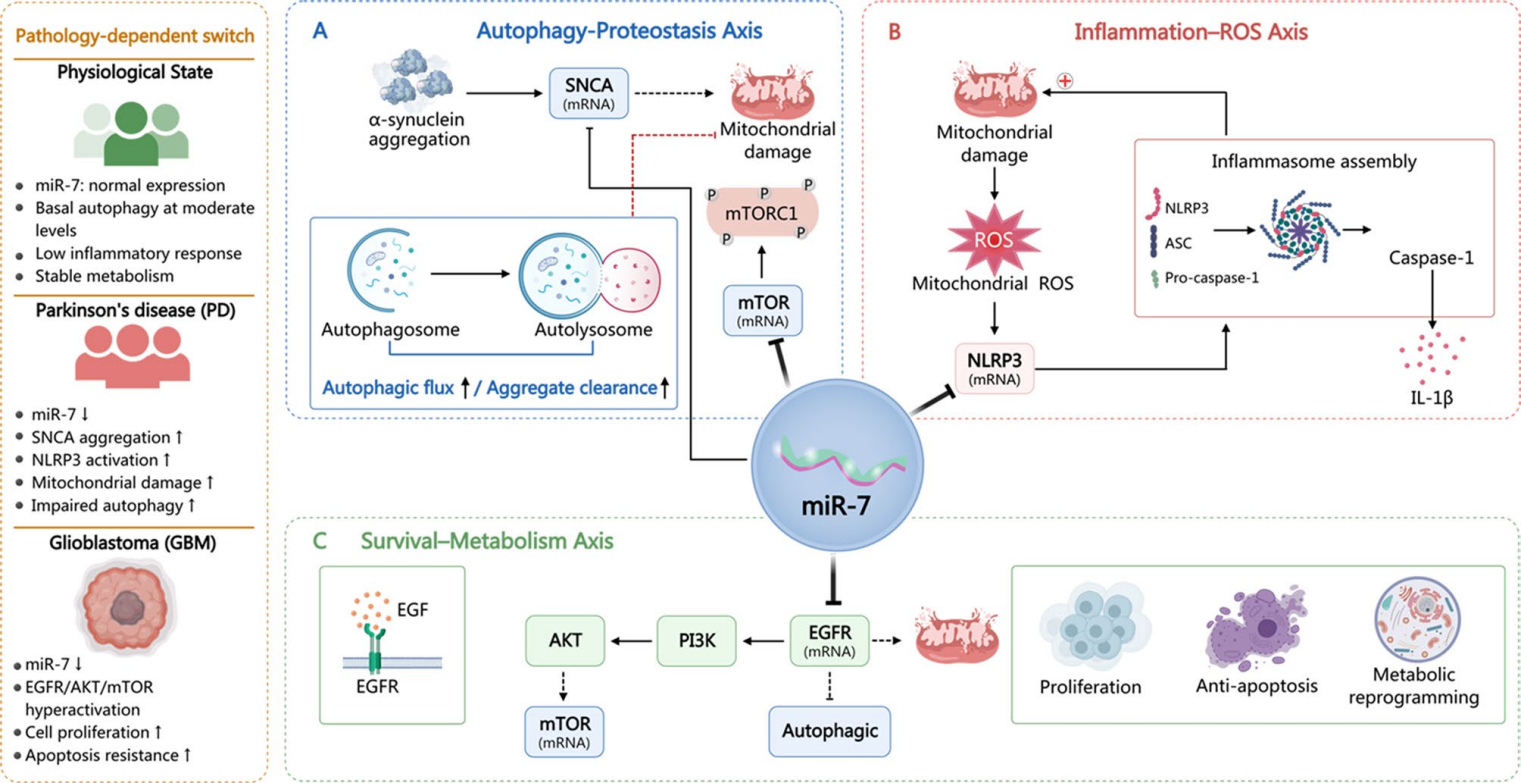
miR-7 orchestrates a pathology-dependent switch across autophagy, inflammation, and survival/metabolism axes in neurodegenerative disease. This diagram depicts miR-7 as a core integrator controlling three major functional axes under pathological conditions (e.g., Parkinson’s disease, other α-synucleinopathies): (**A**) Autophagy-Proteostasis axis – miR-7 modulates autophagic flux and aggregate clearance via mTORC1/mTOR signaling. SNCA (α-synuclein) is transcriptionally linked, and its dysregulation leads to proteostatic failure. (**B**) Inflammation-ROS axis – miR-7 influences ROS production and NLRP3 inflammasome activation, connecting oxidative stress to neuroinflammation. (**C**) Survival-metabolism axis – miR-7 interfaces with EGFR/PI3K/AKT/mTOR cascade, regulating cell survival and metabolic adaptation. The pathology-dependent switch determines which axis dominates: under disease conditions, miR-7 levels or activity shift the balance from protective autophagy toward inflammatory ROS/NLRP3 signaling, while modulating survival pathways. All key nodes are annotated with “(mRNA)” to indicate transcriptional/post-transcriptional regulation. The model highlights miR-7 as a master rheostat that integrates phenotypic outcomes (autophagic failure, inflammation, metabolic stress) and disease progression in synucleinopathies

Furthermore, miR-7 exerts neuroprotective effects through multiple mechanisms, including inhibition of NLRP3 inflammasome activation [31, 32], regulation of mitochondrial function (e.g., by targeting VDAC1 and Sirt2) [18, 40], and promotion of autophagic degradation of α-synuclein [41]. For example, miR-7 alleviates MPP+-induced neuronal apoptosis by suppressing the expression of Bax and Sirt2 [18]; by targeting voltage-dependent anion channel 1 (VDAC1), it stabilizes the mitochondrial membrane potential, reduces cytochrome c release and reactive oxygen species production, thereby protecting dopaminergic neurons from MPP+ toxicity [40]; it also accelerates the clearance of intracellular and extracellular α-synuclein aggregates by promoting autophagy-related pathways [41] (Fig. 2A). These multi-target effects make miR-7 a highly promising intervention target for PD treatment.

Alzheimer’s disease (AD) is pathologically characterized by the formation of Aβ plaques and hyperphosphorylation of tau protein. miR-7 participates in the pathogenesis and progression of AD by regulating the insulin signaling pathway and Aβ metabolism. In the brains of AD patients, miR-7 expression levels are elevated and negatively correlate with the expression of IRS-2 and IDE [8]. miR-7 directly targets the mRNAs of INSR, IRS-2, and IDE, inhibiting their expression, thereby impairing cerebral insulin signaling and Aβ degradation, leading to Aβ accumulation and cognitive dysfunction [8]. IDE is a key enzyme for degrading Aβ, and miR-7-mediated inhibition of IDE directly reduces Aβ clearance, exacerbating the pathological burden of AD. Additionally, miR-7 is involved in the regulation of cholesterol metabolism by targeting cholesterol synthesis-related genes such as DHCR24, affecting cholesterol homeostasis in the brain, and abnormal cholesterol metabolism is a significant risk factor for AD pathogenesis [17]. As a sponge for miR-7, ciRS-7 is downregulated in the brains of AD patients, leading to elevated levels of free miR-7 and further enhanced inhibition of its target genes, forming a “ciRS-7-miR-7-IDE/INSR/IRS-2-Aβ” regulatory axis that plays a crucial role in AD progression [34, 35].

#### Cerebrovascular diseases

Cerebrovascular diseases, such as ischemic stroke and intracerebral hemorrhage (ICH), are among the leading causes of neurological dysfunction and death. miR-7 influences the prognosis of these diseases by regulating inflammatory responses, apoptosis, and blood-brain barrier repair processes.

In cerebral ischemia models, transient middle cerebral artery occlusion (MCAO) leads to a marked downregulation of miR-7 and ciRS-7 levels in the ischemic penumbra [20]. The reduction in miR-7 increases the expression of its target gene α-synuclein, which in turn exacerbates ischemic brain injury by promoting apoptosis, abnormal autophagy, mitochondrial fragmentation, and neuroinflammation [20] (Fig. 2B). Concurrently, ciRS-7 expression is also significantly reduced after cerebral ischemia, further enhancing miR-7 activity by diminishing its sponge effect and aggravating α-synuclein-mediated ischemic brain injury [20]. Conversely, overexpression of ciRS-7 can sponge more miR-7, reduce α-synuclein expression, thereby decreasing infarct volume and improving neurological recovery [20]. Moreover, miR-7 participates in the neuroprotective mechanisms of post-ischemic conditioning by targeting VDAC1. Post-ischemic conditioning stabilizes the expression of VDAC1 and VDAC3, while miR-7 is a key factor causing the decline in VDACs expression after ischemia. Inhibiting miR-7 reduces neuronal death and ATP depletion, as well as shrinks the infarct volume [42].

In intracerebral hemorrhage (ICH) models, intracerebroventricular injection of miR-7 agomir alleviates pathological damage, cerebral edema, and neuroinflammation in perihematomal brain tissue by inhibiting the EGFR/STAT3 signaling pathway, thereby improving neurological function scores [28]; simultaneously, miR-7 directly targets NLRP3, inhibiting the activation of the NLRP3 inflammasome in microglia and the release of pro-inflammatory cytokines (such as IL-1β, IL-6, TNF-α), thus reducing secondary brain injury after ICH [33].

#### Brain tumors

Brain tumors, particularly glioblastoma (GBM), are among the most common and highly malignant tumors of the central nervous system. miR-7 is frequently downregulated in GBM and functions as a tumor suppressor, inhibiting the initiation and progression of GBM through multiple mechanisms.

First, miR-7 suppresses the proliferation, migration, and invasion of GBM cells by targeting key molecules in the PI3K/AKT/mTOR signaling pathway (such as PIK3CD and p70S6K), EGFR, and cell cycle regulators (e.g., CDK4) [25, 27, 36]. Second, miR-7 affects GBM cell survival by regulating autophagy: on one hand, it induces autophagy initiation by inhibiting the PI3K/AKT/mTOR pathway; on the other hand, it blocks autophagosome-lysosome fusion by targeting the autophagy-related SNARE proteins STX17 and SNAP29, leading to autophagic flux arrest and cell death [27]. Third, miR-7 reprograms the energy metabolism of GBM cells by inhibiting mitochondrial function and glycolysis, reducing ATP production and suppressing tumor growth [27]. In terms of therapeutic sensitivity, miR-7 enhances the susceptibility of GBM cells to TRAIL-induced apoptosis by targeting XIAP (X-linked inhibitor of apoptosis protein) [43]. Clinical studies also indicate that low expression of miR-7 is associated with poor prognosis in GBM patients [4]. Beyond GBM, miR-7 may also exert tumor-suppressive effects in other brain tumors, such as medulloblastoma, though the specific mechanisms require further elucidation.

#### Other brain diseases

In addition to the major diseases mentioned above, miR-7 is also associated with other neurological disorders. For example, in a spinal cord injury (SCI) model, AAV1-mediated overexpression of miR-7 improves hindlimb motor function by protecting neuronal survival and promoting axonal regeneration [44]. In a retinal detachment (RD) model, reduced miR-7 expression leads to α-synuclein accumulation, activating the PARP1-mediated parthanatos death pathway, while a miR-7 mimic alleviates photoreceptor cell damage by inhibiting α-synuclein expression [38]. In neuroinflammatory diseases, miR-7 negatively regulates neuronal inflammatory responses through synergistic action with RORα, mitigating lipopolysaccharide (LPS)-induced brain tissue inflammation [45]. Furthermore, miR-7 may also be involved in the pathological processes of neuropsychiatric disorders such as schizophrenia and epilepsy, though the specific mechanisms require further investigation.

### Research objectives, scope, and significance

Although miR-7 is a well-established player in various brain diseases, a systematic integration of its roles across different disorders—particularly regarding its complex regulatory networks (e.g., ceRNA mechanisms), emerging mechanisms (e.g., metabolic reprogramming, neuroinflammation), and translational potential—remains lacking. This review aims to fill this gap by comprehensively summarizing the expression changes, mechanisms, and research progress of miR-7 in common brain diseases, including neurodegenerative diseases, cerebrovascular disorders, and brain tumors.

The scope covers the basic biology of miR-7 (discovery, structure, epigenetic regulation, and molecular networks), its involvement in disease-specific pathological processes (Aβ/tau metabolism, α-synuclein aggregation, autophagy, mitochondrial function, inflammatory signaling, and energy reprogramming), and its potential as a biomarker and therapeutic target. Special attention is given to non-coding RNA crosstalk (ceRNA), inflammation-related pathways (e.g., NLRP3 inflammasome), and metabolic regulation (insulin signaling, cholesterol metabolism).

The significance of this review is threefold. (1) Theoretically, it provides a unified framework for understanding how miR-7 acts as a central node linking shared pathophysiological mechanisms (inflammation, protein aggregation, metabolic dysregulation) across different brain diseases. (2) Practically, it evaluates the diagnostic value of miR-7 in body fluids and the therapeutic efficacy of miR-7-based strategies (mimics, antagomirs, AAV/nanocarrier delivery), while addressing challenges such as blood-brain barrier penetration and targeting specificity. (3) Prospectively, it identifies key research gaps (cell-type-specific roles, dynamic network changes, standardized detection methods) and highlights future directions enabled by emerging technologies (single-cell sequencing, responsive nanocarriers), thereby facilitating the translation of miR-7 from basic research to clinical applications. Overall, this review offers a comprehensive reference for researchers and promotes the development of miR-7-based diagnostic and therapeutic approaches for brain diseases.

## The mechanism and research status of miR-7 in common brain diseases

miR-7, a microRNA highly expressed in the mammalian brain, has recently been shown to play a key regulatory role in the pathophysiological processes of various brain diseases. By targeting multiple critical genes, it participates in regulating neurodegenerative changes, inflammatory responses, cellular metabolism, and tumorigenesis, among other biological processes (Fig. 2C). This provides new perspectives for understanding the complex mechanisms of brain diseases and holds promise as a potential diagnostic marker and therapeutic target. This chapter will systematically review the specific mechanisms of action, expression changes, and experimental evidence of miR-7 in neurodegenerative diseases, cerebrovascular diseases, and brain tumors, with a focus on emerging research advancements in inflammation-related pathways, metabolic reprogramming, and non-coding RNA regulatory networks.

### The role of miR-7 in neurodegenerative diseases

Neurodegenerative diseases are a class of chronic neurological disorders characterized by progressive loss of neurons, including Alzheimer’s disease (AD) and Parkinson’s disease (PD), among others. Recent studies have shown that miR-7 plays a crucial role in the pathogenesis and progression of these diseases, and its abnormal expression is closely associated with pathological processes such as protein aggregation, neuroinflammation, oxidative stress, and mitochondrial dysfunction.

#### miR-7 and Alzheimer’s disease

Alzheimer’s disease is the most common neurodegenerative disorder, with its primary pathological features being senile plaques formed by β-amyloid (Aβ) deposition and neurofibrillary tangles resulting from hyperphosphorylation of tau protein. The altered expression of miR-7 in the brain tissue of AD patients and its regulatory role on key pathological proteins have become a research focus. Studies have found that miR-7 levels are significantly elevated in the brains of AD patients and are negatively correlated with the expression of insulin-degrading enzyme (IDE) and insulin receptor substrate 2 (IRS-2) [8]. IDE is a key enzyme responsible for degrading Aβ, while IRS-2 is a critical component of the insulin signaling pathway. Dysfunction of IRS-2 is associated with cerebral insulin resistance, which is considered one of the important pathological mechanisms of AD. By directly targeting the 3’-untranslated region (3’−UTR) of IDE and IRS-2, miR-7 suppresses their expression at the post-transcriptional level, thereby reducing Aβ clearance and exacerbating cerebral insulin resistance, ultimately promoting the pathological progression of AD [8]. Additionally, miR-7 can influence cholesterol metabolism by regulating the liver X receptor (LXR) pathway. Imbalanced cholesterol metabolism is a significant factor contributing to abnormal Aβ production and clearance [8].

In addition to directly regulating Aβ metabolism and insulin signaling, miR-7 is also involved in the complex non-coding RNA regulatory network in AD. CiRS-7, as an endogenous miR-7 sponge, modulates its activity by competitively binding to miR-7. In the brain tissues of AD patients, the expression of ciRS-7 is significantly reduced, leading to an increase in free miR-7 levels, which in turn enhances its inhibitory effect on target genes (such as UBE2A) [34, 35]. UBE2A is a key enzyme in the ubiquitin-proteasome system involved in Aβ degradation, and its downregulation leads to reduced Aβ clearance, accelerating the formation of senile plaques [34]. The imbalance in this circRNA-miRNA-mRNA regulatory axis is considered one of the important epigenetic mechanisms in AD pathogenesis, providing a new dimension for understanding the complexity of AD. Furthermore, miR-7 can regulate the final step of cholesterol biosynthesis by targeting the DHCR24 gene, and dysregulated cholesterol metabolism is a shared risk factor for AD and cardiovascular diseases [17]. In the APP/PS1 transgenic AD mouse model, intracerebral injection of miR-7 significantly reduced DHCR24 expression, suggesting a potential regulatory link between miR-7, cholesterol metabolism, and AD pathology [17]. These findings reveal the multifaceted role of miR-7 in the pathophysiology of AD, involving Aβ metabolism, insulin resistance, cholesterol balance, and non-coding RNA network regulation, offering potential targets for the diagnosis and treatment of AD.

#### miR-7 and Parkinson’s disease

Parkinson’s disease is the second most common neurodegenerative disorder, primarily characterized by the loss of dopaminergic neurons in the substantia nigra pars compacta and the formation of Lewy bodies (mainly composed of α-synuclein, α-syn). The role of miR-7 in PD has been extensively studied, involving key mechanisms such as α-syn regulation, neuroinflammation, and mitochondrial dysfunction.

##### Regulation of miR-7 and α-synuclein

The abnormal aggregation of α-synuclein (α-syn) is a core pathological event in Parkinson’s disease (PD), and the post-transcriptional regulation of α-syn expression by miR-7 is an important mechanism through which it contributes to PD pathogenesis. Studies have confirmed that miR-7 directly targets the 3’-UTR of the SNCA gene (which encodes α-syn), inhibiting α-syn synthesis [5, 6, 39]. In the substantia nigra of PD patients and MPTP-induced PD model animals, miR-7 expression is significantly reduced, leading to increased α-syn expression and promoting its aggregation and toxic effects [6, 39]. More importantly, miR-7 not only inhibits α-syn synthesis but also accelerates the clearance of preformed α-syn aggregates by promoting autophagy [41]. In neuronal cell models, overexpression of miR-7 activates the autophagy pathway, enhances α-syn degradation, and reduces the accumulation of intracellular α-syn aggregates [41]. This dual regulatory role makes miR-7 a highly promising therapeutic target for PD, as it can both reduce α-syn production at its source and enhance the cell’s ability to clear its toxic aggregates.

The regulation of α-syn by miR-7 is also influenced by other non-coding RNAs. For example, circSNCA (a circular RNA produced by the reverse splicing of the SNCA gene) can act as a ceRNA, upregulating the expression of α-syn by adsorbing miR-7 [46]. In MPP+-induced PD cell models, elevated circSNCA expression sponges miR-7, relieving its inhibitory effect on α-syn, leading to α-syn accumulation and neuronal damage [46]. Additionally, pramipexole (a dopamine receptor agonist commonly used in PD treatment) can downregulate circSNCA expression, restoring miR-7-mediated inhibition of α-syn, thereby exerting a neuroprotective effect [46]. These studies reveal the molecular mechanisms by which miR-7 influences α-syn expression and aggregation through a complex RNA regulatory network in PD, providing new insights for developing RNA-targeted therapeutic strategies for PD.

##### miR-7 and neuroinflammation

Neuroinflammation plays a significant role in the neurodegeneration of dopaminergic neurons in Parkinson’s disease (PD), and miR-7 plays a critical role in regulating PD-associated neuroinflammatory responses. Overactivation of the NLRP3 inflammasome is a key driver of neuroinflammation, leading to the maturation and release of pro-inflammatory cytokines such as IL-1β and IL-18, which exacerbate neuronal damage. Studies have shown that miR-7 can directly target the 3’-UTR of NLRP3, inhibiting its expression and thereby negatively regulating the activation of the NLRP3 inflammasome [31, 32, 47]. In MPTP-induced PD mouse models, miR-7 expression is reduced in the substantia nigra of the midbrain, accompanied by NLRP3 inflammasome activation, microglial activation, and increased release of pro-inflammatory cytokines. Conversely, overexpression of miR-7 via viral vectors significantly suppresses NLRP3 inflammasome activation, alleviating neuroinflammation and the loss of dopaminergic neurons [47]. Additionally, miR-7 levels are elevated in peripheral blood mononuclear cells of PD patients, which may represent a compensatory response to the excessive activation of the NLRP3 inflammasome [32].

The regulation of neuroinflammation by miR-7 also involves other inflammation-related pathways. For example, miR-7 can alleviate microglia-mediated secondary inflammatory responses by suppressing the expression of TLR4 (Toll-like receptor 4) [48]. In an intracerebral hemorrhage model, miR-7 targeted TLR4, inhibiting the activation of the TLR4/TRAF6/NF-κB signaling pathway and reducing the release of pro-inflammatory cytokines (such as TNF-α and IL-1β) [48]. Although this study was conducted in an intracerebral hemorrhage model, TLR4-mediated neuroinflammation is also involved in the pathological process of PD, suggesting that miR-7 may exert anti-inflammatory effects in PD through a similar mechanism. Additionally, SNHG1 can act as a ceRNA to adsorb miR-7, relieving its inhibition of NLRP3 and thereby promoting neuroinflammation [31]. In LPS-stimulated microglia and MPTP-induced PD mouse models, upregulated SNHG1 expression increased NLRP3 inflammasome activation by sponging miR-7, while knocking down SNHG1 restored miR-7 expression, suppressed inflammatory responses, and protected dopaminergic neurons [30]. These studies indicate that miR-7 plays a crucial negative regulatory role in the neuroinflammatory process of PD by directly targeting inflammatory factors or participating in the ceRNA regulatory network.

##### miR-7 and mitochondrial dysfunction

Mitochondrial dysfunction is one of the core mechanisms in the pathogenesis of PD, closely associated with oxidative stress, abnormal energy metabolism, and apoptosis. miR-7 plays a crucial role in maintaining neuronal mitochondrial homeostasis by regulating proteins related to mitochondrial structure and function. Voltage-dependent anion channel 1 (VDAC1) is the primary channel protein on the outer mitochondrial membrane, involved in the transport of energy metabolites and the transmission of apoptotic signals. miR-7 can directly target the 3’-UTR of VDAC1, inhibiting its expression, thereby reducing MPP+-induced loss of mitochondrial membrane potential, cytochrome c release, and reactive oxygen species (ROS) generation, protecting neurons from apoptosis [40]. In SH-SY5Y cells and primary neurons, overexpression of miR-7 significantly ameliorates MPP+-induced mitochondrial fragmentation and maintains normal mitochondrial function [40].

Additionally, miR-7 influences mitochondrial function by regulating other targets. For example, miR-7 can target and silence Sirtuin 2 (Sirt2), a deacetylase involved in regulating mitochondrial function and cell survival [18]. In MPP+-induced PD cell models, miR-7 alleviates mitochondrial damage and neuronal apoptosis by suppressing Sirt2 expression [18]. Bax, a pro-apoptotic protein of the Bcl-2 family, forms pores in the mitochondrial membrane, promoting cytochrome c release. miR-7 directly targets Bax and inhibits its expression, thereby reducing MPP+-induced mitochondrial-dependent apoptosis [18]. These studies demonstrate that miR-7 plays a crucial role in maintaining mitochondrial structural integrity, inhibiting oxidative stress, and suppressing apoptosis by targeting multiple mitochondrial function-related proteins, highlighting its importance as a key regulatory factor in protecting mitochondrial function in PD.

##### Integrating miR-7 networks: the autophagy-inflammation-metabolism axis

Although the SNCA, NLRP3, EGFR/PI3K/AKT/mTOR, and mitochondrial pathways are often described separately, they constitute a functional network through the autophagy-inflammation-metabolism triangle. Within this network, miR-7 acts as a “homeostatic tuner”: on one hand, it reduces protein aggregation burden by inhibiting SNCA [41, 49, 50]; on the other hand, it relieves the inhibition of autophagy by downregulating the EGFR/AKT/mTOR axis, thereby promoting the clearance of aggregated proteins and damaged mitochondria [51, 52]. Simultaneously, miR-7 limits excessive inflammation by directly targeting NLRP3 or indirectly reducing mitochondrial ROS [33]. Conversely, SNCA aggregation can directly damage mitochondrial complex I, increase ROS, and further activate NLRP3, forming a positive feedback loop [53]. In addition, EGFR/AKT signaling not only promotes cell survival but also inhibits autophagy in an mTOR-dependent manner, thereby impairing SNCA clearance [54, 55]. Consequently, loss of miR-7 function simultaneously releases the brakes on these three hubs, leading to synergistic deterioration of aggregation load, inflammatory level, and metabolic stress. Future therapeutic strategies should aim to restore the parallel regulation of these three axes by miR-7 rather than intervening in a single pathway (Fig. 2).

#### miR-7 and other neurodegenerative diseases

Apart from AD and PD, the role of miR-7 in other neurodegenerative diseases has also begun to attract attention, although related research is relatively limited. For example, in Huntington’s disease (HD), the expression of miR-7 may be altered and participate in the disease process by regulating the expression of the *HTT* gene (which encodes huntingtin), but the specific mechanisms require further investigation. In multiple system atrophy (MSA), the abnormal aggregation of α-syn is also a core pathological feature. Given the regulatory role of miR-7 on α-syn, it is speculated that miR-7 may play a similar role in MSA. Additionally, studies have found that miR-7 plays an important role in the development of dopaminergic neurons by inhibiting the Wnt/β-catenin signaling pathway and activating the Shh signaling pathway, regulating the proliferation and differentiation of dopaminergic neuronal progenitor cells, as well as the balance between oligodendrocyte and dopaminergic neuronal fates [3]. This finding suggests that miR-7 may have potential applications in neural repair and regeneration in neurodegenerative diseases by influencing neurogenesis and cell fate determination.

In other neurodegenerative diseases such as spinocerebellar ataxias (SCAs), the expression and function of miR-7 have not been fully studied. However, given its broad roles in neuronal survival, inflammation regulation, and protein homeostasis, it is reasonable to believe that miR-7 may be involved in the pathological processes of these diseases. Future research is needed to explore the specific mechanisms of miR-7 in these neurodegenerative diseases, thereby expanding our understanding of its universal regulatory role in neurological disorders.

### The role of miR-7 in cerebrovascular diseases

Cerebrovascular diseases refer to neurological dysfunctions caused by impaired cerebral blood supply due to vascular pathologies in the brain, including cerebral ischemia/reperfusion injury and cerebral hemorrhage, and are characterized by high incidence, disability, and mortality rates. Recent studies have shown that miR-7 plays a crucial regulatory role in the pathophysiological processes of cerebrovascular diseases, involving key aspects such as inflammatory responses, angiogenesis, and cell death, thereby offering new potential targets for the diagnosis and treatment of these conditions (Table 1).

**Table 1:** Different expression of miR-7 and its regulatory mechanism in brain diseases

Diseases	Expression of miR-7	Core mechanism	Main targets	Ref.
Parkinson’s disease	Significantly decreased	Promote the degradation of α-Syn and its aggregates, anti-neuroinflammatory effects, antioxidant effects.	SNCA	[5, 31, 41, 47, 56–58]
			SNHG1	
			NLRP3	
			Keap1	
Alzheimer’s disease	Significantly upregulated	Aβ clearance defect, inflammation	UBE2A	[8, 34, 59, 60]
			APP	
			BACE1	
Glioblastoma	Decreased	compromise the resolution of autophagy, cellular energy metabolism, and extracellular matrix remodeling	AKT	[27, 61–64]
			PI3K	
			EGFR	
			FAK	
			IRS	
Ischemic stroke	First decreased and then increased.	anti-neuroinflammation effects, protect against mitochondrial damage, neuroprotective	α-Syn	[20, 42, 65]
			VDAC1	
			Herpud2	
Intracerebral hemorrhage	Decreased	Relieve inflammatory, antagonize astrocyte activation	EGFR/STAT3	[33, 48, 66]
			NLRP3	
			TLR4	

#### miR-7 and cerebral ischemia/reperfusion injury

Cerebral ischemia/reperfusion injury is a major pathological process following ischemic stroke, involving complex mechanisms such as oxidative stress, inflammatory response, excitotoxicity, apoptosis, and autophagy. The altered expression and regulatory role of miR-7 in cerebral ischemia/reperfusion injury have become a research hotspot and demonstrate significant therapeutic potential.

##### miR-7 and inflammatory response

Inflammatory response is one of the core mechanisms of cerebral ischemia/reperfusion injury, involving a cascade of events including microglial activation, neutrophil infiltration, and the release of pro-inflammatory factors. miR-7 plays a crucial role in regulating the inflammatory response following cerebral ischemia. Studies have found that in the rat middle cerebral artery occlusion (MCAO) model, the expression of miR-7 in the ischemic brain tissue significantly decreases after ischemia/reperfusion [67–69]. Overexpression of miR-7 significantly inhibits the excessive activation of microglia and the release of pro-inflammatory factors (such as TNF-α, IL-1β, IL-6) after ischemia, thereby alleviating neuroinflammation [65]. This mechanism may be related to miR-7 targeting the endoplasmic reticulum stress-related protein Herpud2. Herpud2 is an endoplasmic reticulum stress-induced protein whose expression is upregulated after cerebral ischemia/reperfusion, exacerbating inflammatory responses and cellular damage by activating the endoplasmic reticulum stress pathway. miR-7 can directly bind to the 3’-UTR of Herpud2, inhibiting its expression and thus mitigating endoplasmic reticulum stress-mediated inflammation and cellular damage [65]. Additionally, miR-7 may also function by regulating other inflammatory pathways, such as the NLRP3 inflammasome, although this has not been directly confirmed in cerebral ischemia models. However, studies in intracerebral hemorrhage and Parkinson’s disease models suggest this possibility [33, 47].

##### miR-7 and angiogenesis

After cerebral ischemia, promoting angiogenesis in the ischemic region is crucial for improving local cerebral blood flow and facilitating neurological recovery. The role of miR-7 in angiogenesis regulation exhibits duality, which may be related to cell type, the stage of ischemia, and the microenvironment. On one hand, some studies suggest that miR-7 may inhibit angiogenesis. For example, in the chicken embryo chorioallantoic membrane model and human umbilical vein endothelial cells (HUVEC), miR-7 inhibits the proliferation, migration, and tube formation ability of vascular endothelial cells by targeting KLF4 (Kruppel-like factor 4) [70]. KLF4 is an important transcription factor that activates the expression of pro-angiogenic genes such as VEGF. In cerebral ischemia models, electroacupuncture stimulation at the Shuigou acupoint can promote angiogenesis in ischemic brain tissue by inhibiting miR-7 expression, thereby relieving its suppression of the KLF4/VEGF and ANG-2 (angiopoietin-2) pathways [71]. This suggests that inhibiting miR-7 after cerebral ischemia may be beneficial for vascular regeneration and neural repair.

However, on the other hand, some studies have shown that miR-7 may indirectly affect angiogenesis by regulating mitochondrial function and cellular metabolism. In glioma research, miR-7 reduces tumor angiogenesis by inhibiting mitochondrial function and glycolysis [27]. Although this finding was observed in a tumor model, it suggests that miR-7‘s regulation of angiogenesis may be cell-context-dependent. During different stages of cerebral ischemia, the expression and function of miR-7 may undergo dynamic changes, and its ultimate impact on angiogenesis depends on the combined effects of multiple factors. Therefore, in-depth research into the specific regulatory mechanisms of miR-7 on angiogenesis during different phases of cerebral ischemia and in different cell types is crucial for developing miR-7-based therapeutic strategies.

##### miR-7 and cell death

Cerebral ischemia/reperfusion injury can induce neuronal death through multiple pathways, including apoptosis, necrosis, autophagy, and ferroptosis. miR-7 plays a crucial role in inhibiting neuronal death after cerebral ischemia, primarily by regulating α-synuclein and mitochondrial function. Studies have shown that the downregulation of miR-7 following cerebral ischemia leads to a reduction in its inhibitory effect on α-syn, resulting in increased α-syn expression. The accumulation of α-syn can cause mitochondrial fragmentation, oxidative stress, and abnormal autophagy, ultimately leading to neuronal death [67–69]. In the MCAO model, miR-7 knockout mice exhibited significantly larger cerebral infarct volumes and more severe neurological deficits; however, administration of miR-7 mimics reduced mitochondrial damage and neuronal apoptosis, decreased infarct size, and improved neurological function by inhibiting α-syn expression [67, 68]. This protective effect was abolished in α-syn knockout mice, confirming that the neuroprotective role of miR-7 depends on the inhibition of α-syn [67].

Additionally, miR-7 directly inhibits apoptosis by targeting other apoptosis-related genes. For instance, miR-7 can target the pro-apoptotic proteins Bax and Sirt2, suppressing MPP^+^ -induced neuronal apoptosis [18], a mechanism that may also apply to cerebral ischemia models. miR-7 also regulates the key component of the mitochondrial permeability transition pore (mPTP), VDAC1, to maintain mitochondrial membrane potential and reduce cytochrome c release, thereby inhibiting the mitochondrial-dependent apoptotic pathway [40, 42]. In global cerebral ischemia models, upregulation of miR-7 expression leads to decreased VDAC1 expression, exacerbating neuronal damage; whereas inhibiting miR-7 restores VDAC1 expression, maintains neuronal Ca^2+^ buffering capacity and mitochondrial function, and reduces neuronal death in the hippocampal CA1 region [42]. This suggests that the regulation of VDAC1 by miR-7 may exhibit different effects depending on the ischemia model (focal vs. global ischemia) or the stage of ischemia, and its specific mechanisms and clinical implications require further elucidation.

#### miR-7 and intracerebral hemorrhage

Intracerebral hemorrhage (ICH) is a severe cerebrovascular disease, and its secondary brain injury involves a series of pathological processes such as perihematomal edema, inflammatory response, blood-brain barrier disruption, and neuronal death. Recent studies have shown that miR-7 plays a crucial role in neuroprotection after ICH, primarily by inhibiting inflammatory responses and reducing astrocyte activation.

##### miR-7 and NLRP3 inflammasome-mediated neuroinflammation

After ICH, hemoglobin and its degradation products (such as heme and iron ions) released from the hematoma can activate the NLRP3 inflammasome in microglia, triggering a strong neuroinflammatory response and exacerbating brain tissue damage. miR-7 has been identified as a key negative regulator of the NLRP3 inflammasome [7]. In rat ICH models, miR-7 expression in the perihematomal brain tissue decreased, while the expression of NLRP3 inflammasome components (NLRP3, ASC, caspase-1) and downstream pro-inflammatory cytokines (IL-1β, IL-18) significantly increased [33]. Intraventricular injection of miR-7 mimics significantly suppressed NLRP3 expression, reduced inflammasome activation and pro-inflammatory cytokine release, thereby alleviating neuroinflammation, cerebral edema, and neurological deficits [33]. Dual-luciferase reporter assays confirmed that miR-7 directly binds to the 3’-UTR of NLRP3, inhibiting its expression at the post-transcriptional level [33]. This finding reveals a novel mechanism by which miR-7 regulates neuroinflammation after ICH by targeting the NLRP3 inflammasome.

In addition to the NLRP3 inflammasome, miR-7 can also alleviate the inflammatory response after ICH by regulating the TLR4/NF-κB signaling pathway. In rat ICH models and LPS-stimulated microglia, miR-7 expression is decreased while TLR4 expression is increased. Overexpression of miR-7 can directly target TLR4, inhibit the activation of the TLR4/TRAF6/NF-κB pathway, and reduce the production of pro-inflammatory factors such as TNF-α and IL-1β, thereby mitigating microglia-mediated secondary brain injury [48]. These findings suggest that miR-7 exerts its anti-inflammatory and neuroprotective effects after ICH through multiple inflammatory pathways.

miR-7 also participates in the pathological process after ICH by inhibiting the overactivation of astrocytes. Following ICH, astrocytes become activated, proliferate, and express glial fibrillary acidic protein (GFAP), playing roles in blood-brain barrier repair and inflammation regulation. However, excessive activation can lead to glial scar formation, which hinders neural regeneration. The epidermal growth factor receptor (EGFR)/signal transducer and activator of transcription 3 (STAT3) pathway is a key signaling pathway regulating astrocyte activation. In a rat ICH model, miR-7 expression was reduced in the perihematomal brain tissue, while the phosphorylation levels of EGFR and STAT3 were increased. Injection of miR-7 mimics, by directly targeting the 3’-UTR of EGFR, inhibited the activation of the EGFR/STAT3 pathway, reduced GFAP expression and astrocyte activation, thereby alleviating cerebral edema and neurological deficits [28]. This study reveals another important mechanism by which miR-7 exerts neuroprotective effects after ICH through suppressing astrocyte overactivation and inflammatory responses.

### The role of miR-7 in brain tumors

Brain tumors are among the most severe diseases of the central nervous system, with glioblastoma multiforme (GBM) being the most common and highly malignant type, exhibiting an extremely poor prognosis. In recent years, significant progress has been made in understanding the role of miR-7 as a potential tumor suppressor in brain tumors, particularly GBM, involving mechanisms such as cell proliferation, apoptosis, autophagy, metabolic reprogramming, and extracellular matrix remodeling.

#### Regulation of miR-7 on the occurrence and development of glioma

Glioblastoma is the most common primary malignant brain tumor in adults, characterized by high invasiveness, proliferative capacity, and treatment resistance. The expression of miR-7 is often significantly reduced in GBM tissues and cell lines, and its low expression is closely associated with the malignant phenotype of the tumor and poor prognosis [27, 36, 43, 72]. By targeting multiple oncogenes and signaling pathways, miR-7 plays a broad-spectrum tumor-suppressive role in the development and progression of GBM.

##### miR-7 and cell proliferation, apoptosis, and invasion

miR-7 inhibits the malignant progression of GBM by regulating multiple signaling pathways involved in cell proliferation and apoptosis. The epidermal growth factor receptor (EGFR) is one of the most commonly mutated oncogenes in GBM, and its abnormal activation drives sustained proliferation of tumor cells. miR-7 directly targets the 3’-UTR of EGFR, inhibiting its expression and the activation of the downstream PI3K/AKT/mTOR signaling pathway, thereby suppressing GBM cell proliferation and inducing apoptosis [27, 28, 72]. In GBM xenograft models, overexpression of miR-7 significantly reduces the levels of EGFR and phosphorylated AKT, inhibiting tumor growth [72] (Fig. 2C). Additionally, miR-7 enhances tumor cell sensitivity to tumor necrosis factor-related apoptosis-inducing ligand (TRAIL) by upregulating the expression of death receptor 5 (DR5). By inhibiting the EGFR/AKT pathway, miR-7 relieves its suppression of NF-κB, thereby upregulating DR5 transcription [72]. In TRAIL-resistant GBM cells and patient-derived GBM stem cells (GSCs), the combination of miR-7 mimics and stem cell-secreted soluble TRAIL (S-TRAIL) significantly enhances antitumor effects and even completely eradicates tumors [72].

miR-7 also targets other key regulatory factors to affect the proliferation and apoptosis of GBM cells. For example, miR-7 can directly target X-linked inhibitor of apoptosis protein (XIAP), a potent caspase inhibitor, thereby promoting TRAIL-induced apoptosis [43]. miR-7 can also inhibit the cell cycle progression and proliferation of GBM cells by targeting cyclin-dependent kinase 4 (CDK4) [36]. The long non-coding RNA HMMR-AS1 is highly expressed in GBM and promotes tumor cell proliferation and invasion by sponging miR-7, thereby relieving its inhibition of CDK4. In contrast, the anesthetic sevoflurane can inhibit HMMR-AS1, restore miR-7‘s regulation of CDK4, and exert anti-tumor effects [36]. These studies indicate that miR-7, by targeting multiple key nodes such as EGFR/PI3K/AKT/mTOR, DR5/NF-κB, XIAP, and CDK4, forms a complex regulatory network that collectively inhibits the proliferation of GBM cells and promotes their apoptosis.

In terms of cell invasion and metastasis, miR-7 inhibits the invasive ability of GBM by regulating the expression of extracellular matrix-degrading enzymes such as matrix metalloproteinases (MMPs). Although direct evidence is limited, miR-7 has been reported in other tumors (e.g., non-small cell lung cancer) to reduce the secretion of MMP-2 and MMP-9 by inhibiting the EGFR/PI3K/AKT pathway [73], a mechanism that may also apply to GBM. Furthermore, the broad regulatory role of miR-7 in extracellular matrix remodeling (see below) indirectly supports its function in inhibiting GBM invasion.

##### miR-7 and autophagy

Autophagy plays a dual role in GBM, serving as a survival mechanism for tumor cells under stress conditions (such as nutrient deprivation and hypoxia), while also potentially promoting cell death under specific circumstances. The regulation of autophagy by miR-7 exhibits a unique “bidirectional modulation” characteristic—promoting the initiation of autophagy while blocking its late stages, ultimately leading to the stagnation of autophagic flux and cell death. In GBM cells, miR-7 induces the formation of autophagosomes by inhibiting the activity of the PI3K/AKT/mTORC1 signaling pathway, thereby relieving its suppression of autophagy initiation [27]. However, miR-7 simultaneously inhibits the fusion of autophagosomes with lysosomes by targeting key SNARE proteins STX17 (Syntaxin 17) and SNAP29 involved in the autophagosome-lysosome fusion process, thereby blocking the completion of autophagy [27]. This separation of autophagy initiation and degradation stages results in the accumulation of immature autophagosomes within cells, preventing the effective clearance of damaged organelles and proteins, ultimately triggering cellular stress and death. In GBM xenograft models, overexpression of miR-7 can reproduce this autophagic flux stagnation phenomenon in vivo and is associated with the inhibition of tumor growth [27]. This unique regulatory mechanism enables miR-7 to selectively induce GBM cell death by leveraging the autophagy process, offering a novel approach to overcoming therapeutic resistance in GBM.

##### miR-7 and energy metabolism (glycolysis, mitochondrial function)

Metabolic reprogramming is a key feature of tumor cells. GBM cells primarily rely on glycolysis for energy even under aerobic conditions (Warburg effect) and exhibit adaptive changes in mitochondrial function. miR-7 plays a critical role in regulating metabolic reprogramming in GBM cells, achieving a “dual strike” on tumor cell energy metabolism by simultaneously inhibiting glycolysis and mitochondrial function. In GBM cells, overexpression of miR-7 significantly reduces glucose uptake, lactate production, and intracellular ATP levels, indicating its inhibitory effect on glycolysis [27]. The mechanism may involve miR-7 targeting key enzymes in the glycolytic pathway (such as hexokinase and pyruvate kinase) or their upstream regulators, although specific targets require further confirmation. At the same time, miR-7 also significantly inhibits mitochondrial oxidative phosphorylation function, manifested as reduced mitochondrial membrane potential, decreased ATP production, and elevated reactive oxygen species (ROS) levels [27]. This may be partly attributed to miR-7‘s inhibition of VDAC1, which not only regulates apoptosis but also serves as a key channel for mitochondrial metabolite exchange [40, 42]. The dual inhibition of glycolysis and mitochondrial function by miR-7 completely cuts off the energy supply of GBM cells, leading to cell growth arrest and death. This “metabolic collapse” effect is a fundamental basis for miR-7‘s anti-tumor action [27, 51].

##### miR-7 and extracellular matrix remodeling

Remodeling of the extracellular matrix (ECM) is a critical step in the invasion and metastasis of GBM, involving dynamic changes in matrix metalloproteinases (MMPs), integrins, and other ECM components. Recent studies have shown that miR-7 can significantly alter the composition and structure of the ECM in GBM, thereby inhibiting tumor invasion and progression. In GBM xenograft models, overexpression of miR-7 leads to significant changes in the expression profiles of ECM-related genes in tumor tissues, including the downregulation of collagen, fibronectin, and MMPs [27]. These changes may result from miR-7 directly targeting enzymes involved in ECM remodeling or their upstream regulatory transcription factors, although the specific target genes and mechanisms have not yet been fully elucidated. Abnormal ECM remodeling may alter the interaction between tumor cells and the surrounding microenvironment, inhibiting their migration and invasion capabilities. This finding expands the scope of miR-7‘s role in GBM, suggesting that it not only affects the intrinsic properties of tumor cells but also exerts anti-tumor effects by remodeling the tumor microenvironment.

#### Expression and significance of miR-7 in other brain tumors

Although research on miR-7 in brain tumors has primarily focused on GBM, preliminary evidence suggests it may also play a significant regulatory role in other types of brain tumors. For instance, in medulloblastoma, miR-7 expression may be epigenetically regulated, and its low expression is associated with poor prognosis, though its specific functions and mechanisms have not been thoroughly investigated. In different subtypes of glioma, the expression patterns and roles of miR-7 may vary, reflecting the heterogeneity of tumor molecular subtypes. Additionally, in some metastatic brain tumors, such as lung cancer brain metastases and breast cancer brain metastases, miR-7 may function by regulating the invasive and metastatic capabilities of primary tumors or influencing tumor cell colonization in the brain microenvironment. For example, in non-small cell lung cancer, ciRS-7 acts as a sponge for miR-7, upregulating the expression of target genes such as EGFR, CCNE1, and PIK3CD, thereby promoting tumor progression and metastasis [73]. This mechanism may similarly be involved in the process of lung cancer brain metastasis.

The role of miR-7 in other neuroendocrine tumors has also been reported. For example, in multiple myeloma, miR-7 is downregulated by HDAC inhibitors, and overexpression of miR-7 inhibits cell proliferation and induces apoptosis, a mechanism associated with targeting PSME3 (proteasome activator) [74]. Although multiple myeloma is not a primary brain tumor, it may involve the central nervous system, and this study suggests the potential role of miR-7 at the intersection of hematological malignancies and neuro-oncology.

Overall, miR-7 functions as a versatile tumor suppressor, playing a critical regulatory role in various pathophysiological processes of glioma, particularly glioblastoma (GBM). The diversity and synergy of its mechanisms of action provide a robust theoretical foundation and broad application prospects for the development of miR-7-based diagnostic and therapeutic strategies. However, the specific roles and mechanisms of miR-7 in other brain tumors remain to be further explored, which will help expand our understanding of its universality and specificity in nervous system tumors.

### The potential of miR-7 in the diagnosis of brain disorders

#### Detection of miR-7 in body fluids

The detection of miRNA in body fluids holds significant value in the diagnosis of brain diseases due to its minimally invasive nature and repeatability. miR-7, a miRNA highly expressed in brain tissue, exhibits expression changes in blood and cerebrospinal fluid that are closely related to various brain disorders, providing a potential biomarker for early disease diagnosis.

In the field of blood testing, multiple studies have explored the expression levels of miR-7 in the peripheral blood of patients with various brain disorders. For instance, in Parkinson’s disease (PD) models, researchers observed upregulation of miR-7 in the brains of rats but downregulation in peripheral blood. This differential expression may be related to tissue-specific regulation of miR-7 during disease progression [75]. However, another study on the serum of PD patients did not detect significant changes in miR-7 levels, which could be attributed to differences in sample size, detection methods, or disease stages [76]. This discrepancy suggests that stricter experimental designs and standardized detection protocols are needed when considering miR-7 as a blood biomarker for PD. In patients with schizophrenia, peripheral blood miR-7 expression also shows abnormalities. A systematic review indicated dysregulation of hsa-miR-7 in blood samples from schizophrenia patients, potentially involved in the pathophysiological processes of the disease [77, 78]. Furthermore, in patients with idiopathic inflammatory myopathy complicated by interstitial lung disease (IIM/ILD), plasma miR-7 levels were significantly reduced, with an area under the curve (AUC) of 0.8978 for distinguishing IIM/ILD patients from non-ILD patients, demonstrating strong diagnostic potential [79]. This finding suggests that miR-7 may serve as a biomarker not only in central nervous system disorders but also in systemic inflammatory diseases involving the lungs, with its expression changes possibly reflecting the interplay between systemic inflammation and neuroinflammation.

Cerebrospinal fluid (CSF), as a bodily fluid in direct contact with the central nervous system, may more directly reflect pathological changes in the brain through its miR-7 levels. miR-7 is primarily expressed by neurons in the brain, particularly those with sensory or neurosecretory functions [1]. When brain lesions occur, neuronal damage or disruption of the blood-brain barrier may lead to the release of miR-7 into the CSF. For example, in Alzheimer’s disease (AD), miR-7 participates in the clearance of β-amyloid by targeting genes such as UBE2A, and changes in its CSF levels may be associated with the pathological progression of AD [35]. Although there are currently limited studies on directly detecting CSF miR-7 for diagnosing AD or other neurodegenerative diseases, based on miR-7‘s crucial regulatory role in the brain and its expression changes in the blood, it is hypothesized that CSF miR-7 may offer higher diagnostic specificity and sensitivity, warranting further in-depth investigation.

It is noteworthy that the expression of miR-7 is regulated by ceRNA, such as ciRS-7. As a circular RNA, ciRS-7 can adsorb miR-7 through a sponge effect, thereby inhibiting its function [21, 80]. In various cancers, the high expression of ciRS-7 leads to a decrease in miR-7 levels, forming a ciRS-7/miR-7 axis regulatory network [25, 73, 81, 82]. Although there is currently limited research on the correlation between ciRS-7 and miR-7 expression in body fluids in brain diseases, this regulatory mechanism suggests that combined detection of miR-7 and its related ceRNA levels in body fluids may improve diagnostic accuracy more effectively than detecting miR-7 alone. For example, in AD, a decrease in ciRS-7 levels may lead to increased miR-7 expression, which in turn affects disease progression by regulating downstream target genes [35]. Therefore, the ciRS-7/miR-7 ratio in blood or cerebrospinal fluid may serve as a more sensitive diagnostic biomarker for AD.

Furthermore, the epigenetic regulation of miR-7, such as its DNA methylation status, may also influence its expression in bodily fluids. Studies have found that the methylation status of the miR-7 promoter region is associated with the sensitivity of non-small cell lung cancer (NSCLC) patients to cisplatin chemotherapy, and methylated miR-7 may serve as a biomarker for predicting NSCLC prognosis [14, 83]. Although this finding primarily focuses on cancer, it suggests that the epigenetic modification status of miR-7 (such as methylation) may also serve as a diagnostic or prognostic marker for brain diseases. For example, in gliomas, the expression of miR-7 is epigenetically regulated, and its methylation level may be correlated with the malignancy of the tumor [83]. Therefore, detecting the methylation status of miR-7 or the levels of its precursor molecules in bodily fluids may provide new insights for the diagnosis of brain diseases.

However, the detection of miR-7 in body fluids still faces many challenges. First, the abundance of miRNA in body fluids is typically low, requiring highly sensitive detection methods such as quantitative real-time PCR (qRT-PCR), microarray chips, or next-generation sequencing technologies. Second, differences in sample processing and detection methods across studies may lead to inconsistent results, such as the conflicting reports on serum miR-7 levels in PD patients mentioned in studies [75, 76, 84]. Therefore, establishing a standardized miR-7 detection process, including uniform protocols for sample collection, RNA extraction, reverse transcription, and amplification conditions, is crucial for advancing its clinical application. Additionally, factors such as individual differences, physiological states (e.g., age, gender, diet), and comorbidities may also affect miR-7 levels in body fluids, which need to be considered and adjusted for in large-scale clinical studies.

#### Expression and significance of miR-7 in extracellular vesicles

Extracellular vesicles (EVs), including exosomes and microvesicles, are important mediators of intercellular communication, capable of transporting bioactive molecules such as miRNA to transmit information between cells [85–87]. The miRNAs in EVs, protected by the vesicle membrane, exhibit high stability and resistance to RNase degradation, making them more suitable as biomarkers for disease diagnosis than free miRNAs [88, 89]. The enrichment of miR-7 in EVs and its altered expression in brain diseases provide new research directions for non-invasive diagnosis and disease monitoring [1, 90, 91].

miR-7 is abundant in EVs derived from neural cells. Both neurons and glial cells secrete EVs, which not only participate in neural development and synaptic plasticity regulation under normal physiological conditions but also release specific miRNA profiles into the extracellular environment under pathological conditions [1]. For example, in Alzheimer’s disease (AD) models, neurons may regulate the function of surrounding cells by releasing miR-7-enriched EVs in response to the toxic effects of β-amyloid [35]. When neurons are damaged, the content of miR-7 in EVs may change, and such alterations can reflect the pathological state of the brain through the detection of EVs in cerebrospinal fluid or blood [92–94]. Studies have shown that EVs can cross the blood-brain barrier, making it possible to detect neuron-derived EV miR-7 in peripheral blood, paving the way for non-invasive diagnosis of brain diseases [95, 96].

In the field of brain tumors, the expression of miR-7 in EVs holds significant diagnostic and prognostic implications. Glioma is the most common malignant brain tumor, where miR-7 typically functions as a tumor suppressor gene by inhibiting tumor cell proliferation and invasion through suppressing the PI3K/AKT/mTOR pathway and regulating mitochondrial function and glycolysis [27]. The level of miR-7 in tumor cell-derived EVs may align with its expression in tumor tissues, i.e., exhibiting a low expression state. One study identified a 4-miRNA signature (miR-21, miR-10b, miR-7, miR-491) in brain tumor patients, which can distinguish high-grade gliomas, low-grade gliomas, and brain metastases, and is associated with patients’ Karnofsky Performance Status scores [97]. Although this study did not specify whether miR-7 originated from EVs or in a free form, based on the stability and specificity of EVs-miRNA observed in other cancer studies, it is speculated that EVs-miR-7 may be a key component of this signature. Additionally, in non-small cell lung cancer (NSCLC), ciRS-7 is highly expressed in tumor tissues and downregulates miR-7 via a sponging effect, promoting tumor progression. A similar ciRS-7/miR-7 expression pattern may also exist in blood EVs of NSCLC patients with brain metastases, potentially serving as an early warning marker for brain metastasis [73].

The regulatory mechanism of miR-7 in EVs is similar to its intracellular regulation and is influenced by the ceRNA network. ciRS-7, as the main sponge molecule of miR-7, also exists in EVs and may be transmitted between cells via EVs, thereby regulating miR-7 activity in target cells [21, 80]. For example, in hepatocellular carcinoma, ciRS-7 promotes the malignant phenotype of tumor cells by adsorbing miR-7, and EVs containing high levels of ciRS-7 and low levels of miR-7 may participate in the remodeling of the tumor microenvironment and the metastasis process [25, 80]. In brain diseases such as glioblastoma, EVs secreted by tumor cells may be rich in ciRS-7, thereby inhibiting the tumor-suppressive function of miR-7 in surrounding normal brain tissue and promoting tumor infiltration. Therefore, the combined detection of ciRS-7 and miR-7 levels in EVs, as well as their ratio, may more accurately reflect the malignancy and treatment response of tumors than detecting miR-7 alone.

Research on EVs-miR-7 in neurodegenerative diseases is still in its early stages, but there are clues suggesting its potential value. In PD, abnormal aggregation of α-synuclein is one of the main pathological features, and miR-7 can mitigate its neurotoxicity by regulating the expression of α-synuclein [75, 98, 99]. EVs play a significant role in the propagation of α-synuclein, and EVs containing abnormal α-synuclein may also carry miR-7 in an attempt to inhibit further aggregation of α-synuclein. Therefore, the ratio of miR-7 to α-synuclein in cerebrospinal fluid or blood EVs may serve as a monitoring indicator for the progression of PD. In AD, miR-7 affects the clearance of β-amyloid by targeting UBE2A, and the level of EVs-miR-7 may be associated with the burden of amyloid plaques [35]. A study, through integrative analysis of brain regions in AD patients, identified abnormal expression of a 23 non-coding RNA signature, including the miR-7 precursor, suggesting that the processing and maturation of miR-7 may be disrupted in AD, a process that might be reflected in EVs [100].

The main challenges in EVs-miR-7 detection lie in the isolation and purification of EVs and the quantification methods for miRNA. Currently used EV isolation methods, such as ultracentrifugation, immunomagnetic beads, and size exclusion chromatography, each have their own advantages and disadvantages, which may affect the purity and recovery rate of EVs, thereby impacting the accuracy of miR-7 detection. Additionally, the heterogeneity of EVs means that different subpopulations (e.g., neuron-derived, glial cell-derived, tumor cell-derived EVs) may contain varying levels of miR-7. How to specifically isolate and analyze disease-relevant EV subpopulations is key to improving diagnostic specificity. For example, using magnetic beads coated with neuron-specific surface markers (such as L1CAM) or tumor cell-specific markers (such as EGFRvIII) to isolate EVs can enrich EVs from specific sources, thereby enhancing the disease relevance of miR-7 detection [27].

Despite the challenges, EVs-miR-7 holds great potential as a biomarker for brain diseases. Its advantages include: (1) High stability: The membrane structure of EVs protects miR-7 from degradation, extending its half-life in body fluids; (2) Tissue specificity: EVs from different cell sources carry molecular signatures of their parent cells, reflecting pathological changes in specific tissues; (3) Minimally invasive nature: It can be detected through body fluids such as blood or cerebrospinal fluid, avoiding the risks of brain tissue biopsy. Future research needs to optimize EVs isolation and miR-7 detection techniques, conduct large-scale multi-center clinical trials to validate the diagnostic efficacy of EVs-miR-7 in various brain diseases, and explore its synergistic effects when combined with other biomarkers (such as proteins and metabolites) to improve diagnostic accuracy and reliability.

### The value of miR-7 in prognostic assessment of brain diseases

miR-7 not only holds potential for the early diagnosis of brain diseases, but its expression levels and the state of its regulatory network are also closely related to disease prognosis, providing an important basis for clinical prognosis assessment and the formulation of treatment strategies.

In the field of brain tumors, the expression level of miR-7 is significantly associated with patient prognosis. Glioma, as the most common malignant brain tumor, exhibits considerable variation in malignancy and prognosis. Research has found that miR-7 is generally downregulated in glioma tissues and is closely related to tumor grade, depth of invasion, and patient survival rate. A study involving 38 high-grade gliomas, 10 low-grade gliomas, and 5 brain metastases showed that miR-7 is one of the 4-miRNA signatures distinguishing different types of brain tumors and is associated with patients’ Karnofsky Performance Status and survival status [97]. In glioblastoma (GBM), miR-7 suppresses tumor progression by inhibiting autophagy resolution, energy metabolism, and extracellular matrix remodeling. In vivo experiments have demonstrated that miR-7 overexpression significantly inhibits the size and progression of established GBM tumors [27]. This suggests that low miR-7 expression in tumor tissues may indicate a worse prognosis, and restoring miR-7 expression could become a therapeutic strategy to improve outcomes. Additionally, in non-brain tumors such as lung adenocarcinoma, low miR-7 expression is associated with poorer tumor differentiation, more advanced pathological T stage, higher incidence of lymph node metastasis, and shorter overall survival. Multivariate analysis revealed that miR-7 expression status is an independent molecular marker for predicting overall survival in lung adenocarcinoma patients [101]. Although this data pertains to lung adenocarcinoma, given that brain metastasis is a common complication of lung cancer, the expression status of miR-7 in primary tumors may also influence the occurrence and prognosis of brain metastases, warranting further investigation.

The epigenetic modification status of miR-7, particularly methylation, has also been shown to be an important indicator for assessing prognosis. In patients with early-stage non-small cell lung cancer (NSCLC), the methylation status of the miR-7 promoter region is associated with poorer survival, especially in adenocarcinoma patients, where the progression-free survival (PFS) of the miR-7 methylated group is significantly shorter than that of the unmethylated group (median 18.9 months vs. 59.7 months) [83]. This finding suggests that the methylation status of miR-7 may serve as an epigenetic marker for determining tumor aggressiveness and prognosis. In gliomas, although the relationship between miR-7 methylation and prognosis has not been directly studied, existing research indicates that DNA methylation is a key mechanism regulating miRNA expression and is associated with glioma grading and prognosis [14]. Therefore, it is hypothesized that the methylation status of miR-7 may similarly affect the prognosis of glioma patients, with higher methylation levels leading to lower miR-7 expression, increased tumor malignancy, and poorer patient outcomes.

The regulatory network of miR-7, particularly its interaction with ceRNA, also plays a crucial role in prognostic evaluation. ciRS-7, as the primary sponge molecule for miR-7, typically exhibits an inverse correlation in expression levels with miR-7, together forming molecular indicators for prognostic assessment. In hepatocellular carcinoma (HCC), high expression of ciRS-7 is an independent risk factor for microvascular invasion (MVI), which is a significant marker of poor prognosis in HCC [25]. By sequestering miR-7, ciRS-7 relieves the inhibition of miR-7 on its target genes (such as PIK3CD and p70S6K), thereby promoting tumor invasion and metastasis [25]. In glioblastoma, a similar ciRS-7/miR-7 axis may also influence tumor invasion and recurrence, with patients exhibiting high ciRS-7 and low miR-7 expression potentially facing higher recurrence risks and shorter survival. In colorectal cancer, circHIPK3 promotes tumor progression by sponging miR-7, and high expression of circHIPK3 is an independent risk factor for poor prognosis in colorectal cancer patients [102]. Whether this mechanism applies to colorectal cancer cells with brain metastasis and whether the ratio of ciRS-7/circHIPK3 to miR-7 can serve as a prognostic marker for brain metastases warrant further investigation.

Research on the prognostic value of miR-7 in neurodegenerative diseases is relatively limited, but existing evidence suggests its potential. In PD, miR-7 participates in the early stages of the disease by regulating the BDNF/α-synuclein axis [75]. The aggregation and spread of α-synuclein are key to PD progression, and miR-7‘s inhibitory effect on α-synuclein may slow disease advancement. Therefore, PD patients with higher levels of miR-7 in cerebrospinal fluid or blood may experience slower disease progression and better prognoses. In AD, miR-7 affects the clearance of β-amyloid by targeting UBE2A [35]. Sufficient miR-7 expression may indicate a stronger ability to clear β-amyloid, thereby delaying cognitive decline. One study found abnormal expression of miR-7 precursors in the brain regions of AD patients, suggesting that miR-7 biosynthesis may be disrupted in AD, and the extent of this disruption may be related to disease severity and prognosis [100]. Additionally, in schizophrenia, dysregulation of miR-7 may be associated with chronicity and poor prognosis, though direct clinical prognostic data are currently lacking [77, 78].

miR-7 also holds potential value in predicting treatment response, which indirectly affects patient prognosis. In lung adenocarcinoma, overexpression of miR-7 increases cellular sensitivity to cisplatin by targeting Bcl-2 to induce apoptosis [101]. In ovarian cancer, the methylation status of miR-7 is associated with cisplatin resistance, and methylated miR-7 may indicate a poor response to cisplatin chemotherapy [14]. Although these studies focus on chemotherapeutic drugs, they suggest that miR-7 could serve as a biomarker for predicting treatment response. In targeted therapy for glioma, miR-7 expression levels may predict the efficacy of targeted drugs such as EGFR inhibitors, as miR-7 itself is a negative regulator of EGFR [27, 82]. For instance, glioma patients with high miR-7 expression may be more sensitive to EGFR inhibitors, as miR-7 and the drug can synergistically inhibit the EGFR pathway. Therefore, detecting miR-7 expression levels can help identify patients most likely to benefit from specific targeted therapies, thereby improving prognosis.

However, applying miR-7 as a prognostic biomarker in clinical practice still faces numerous challenges. First, differences in detection methods, sample types (tissue, blood, cerebrospinal fluid, EVs), and cutoff values across studies make it difficult to directly compare and standardize results. For instance, studies on serum miR-7 levels in PD patients have yielded conflicting findings [75, 76]. Second, miR-7 expression is regulated by various factors, such as genetic background, environmental factors, comorbidities, and therapeutic interventions, all of which may affect the stability of miR-7 as a prognostic biomarker. For example, vitamin D supplementation can reduce plasma miR-7 levels in prediabetes patients and is associated with improved glycemic control [103]. Whether vitamin D levels influence miR-7 expression and prognosis in patients with brain disorders requires further investigation. Additionally, the regulatory network of miR-7 is complex, and detecting miR-7 alone may not be sufficient to comprehensively reflect disease prognosis. Multi-marker joint analysis, incorporating its target genes (e.g., EGFR, PIK3CD, Bcl-2), upstream regulators (e.g., ciRS-7, LincIN), and other miRNAs (e.g., miR-21, miR-10b), is needed to improve the accuracy of prognostic evaluation [97, 104].

To promote the clinical application of miR-7 in the prognosis assessment of brain diseases, the following tasks need to be carried out in the future: (1) establish standardized miR-7 detection methods and unified cutoff values to ensure the reliability and comparability of test results; (2) conduct large-scale, multi-center prospective cohort studies to validate the value of miR-7 and its regulatory network in the prognosis assessment of various brain diseases; (3) integrate multi-dimensional data such as imaging and clinical indicators to construct a comprehensive prognostic model that includes miR-7; (4) explore the combined application of miR-7 as a therapeutic target and prognostic marker to achieve personalized treatment and prognosis monitoring.

### Limitations and prospects of miR-7 as a biomarker

Although miR-7 demonstrates significant potential in the diagnosis and prognostic assessment of brain diseases, its clinical translation as a biomarker still faces numerous limitations, while also holding broad development prospects. A deep understanding of these limitations and active exploration of solutions are key to advancing miR-7 from basic research to clinical application.

#### Limitations

The limitations of miR-7 as a biomarker are first reflected in the contradiction between its tissue-specific expression and detection in body fluids. miR-7 is highly enriched in the brain, particularly in neurons with sensory or neurosecretory functions [1]. Theoretically, neuronal damage or dysfunction caused by brain diseases could lead to changes in miR-7 levels in the cerebrospinal fluid, which may then permeate into the peripheral blood in small amounts. However, the sources of miR-7 in peripheral blood are complex; in addition to brain tissue, it may also originate from other organs and cell types, such as the liver, lungs, and immune cells [14, 79, 101]. This makes it difficult to accurately attribute changes in peripheral blood miR-7 to brain diseases. For example, in patients with IIM/ILD, whether the decrease in plasma miR-7 levels reflects a systemic inflammatory state or neuromuscular involvement still requires further differentiation [79]. Additionally, different brain diseases may cause similar trends in miR-7 expression, such as a decrease in peripheral blood miR-7 in both glioma and PD [75, 76, 105], which reduces its diagnostic specificity.

Secondly, the regulatory network of miR-7 is complex, and its expression level is influenced by multiple factors, which increases the difficulty of interpreting it as a biomarker. The expression of miR-7 is not only regulated by genetic transcription and epigenetic mechanisms (such as DNA methylation) [14, 83, 106], but is also tightly controlled by ceRNA networks, most notably the sponge effect of ciRS-7 [21, 80]. ciRS-7 adsorbs miR-7 through numerous binding sites, preventing it from inhibiting target genes. In various cancers, high expression of ciRS-7 leads to functional inactivation of miR-7, while the expression level of miR-7 may not be significantly reduced [25, 73, 81, 82]. Therefore, simply detecting the expression level of miR-7 may not accurately reflect its actual biological activity. Additionally, other ceRNAs such as LincIN and circHIPK3 can also influence miR-7 function through regulation [102, 104]. This complex regulatory mechanism affects the specificity and sensitivity of miR-7 as a biomarker, necessitating simultaneous consideration of the status of its regulatory factors.

The standardization and stability of detection technology are also major obstacles to the clinical application of miR-7. Currently, there are various methods for detecting miRNA, including qRT-PCR, microarray chips, and next-generation sequencing, with differences in sensitivity, specificity, and reproducibility among these methods. For example, while qRT-PCR has high sensitivity, it is susceptible to the quality of RNA extraction, primer design, and amplification efficiency. Sequencing technologies can discover novel miRNAs but are costly and involve complex data analysis. In miR-7 detection, inconsistencies in reference genes, sample processing methods, and data analysis pipelines across different studies make results difficult to compare and reproduce. For instance, in the detection of serum miR-7 in PD patients, one study did not observe significant changes, which may be related to sample size, detection methods, or disease stage [76]. Additionally, miRNA exists in various forms in body fluids (free, bound to proteins, or within EVs), and the stability of miR-7 differs across these forms. Detecting different forms of miR-7 may yield different results [1, 97].

The clinical validation evidence for miR-7 as a biomarker remains insufficient. Most studies are still in the exploratory phase with small sample sizes, lacking validation through large-scale, multi-center, prospective clinical trials. For instance, although miR-7 was included in a 4-miRNA signature for distinguishing different brain tumors, the sample size of that study was only 53 cases [97]. In neurodegenerative diseases, research on miR-7 as a prognostic marker is even scarcer, with a lack of long-term follow-up data. Additionally, the combined application value of miR-7 with existing clinical indicators (such as the Aβ42/40 ratio in AD, α-synuclein aggregation in PD, and IDH mutation status in gliomas) remains unclear, and whether it can provide prognostic information independent of existing markers still requires validation [97, 107].

#### Outlook

Despite the aforementioned limitations, the prospects of miR-7 as a biomarker for brain diseases remain broad. With the deepening of research and technological advancements, these issues are expected to be gradually resolved, promoting the clinical translation of miR-7.

First, to address the issue of tissue specificity, future research should focus on detecting miR-7 in cerebrospinal fluid (CSF) and combine it with extracellular vesicle (EV) isolation techniques to improve the specificity of peripheral blood detection. CSF directly reflects the state of the central nervous system, and changes in its miR-7 levels may have greater diagnostic value than those in peripheral blood. For instance, developing highly sensitive CSF miR-7 detection methods, combined with other Alzheimer’s disease biomarkers (such as phosphorylated tau protein), could significantly enhance early diagnosis rates [35, 100]. For peripheral blood detection, isolating neuron-derived EVs (e.g., EVs expressing markers like NeuN and CD31) through methods such as immunomagnetic beads can enrich miR-7 originating from brain tissue and reduce interference from other sources [1, 97]. For example, measuring the ratio of miR-7 to ciRS-7 in neuron-derived EVs may more accurately reflect the state of brain diseases.

Secondly, in-depth analysis of the regulatory network of miR-7 and the development of multi-marker combined detection strategies are key to improving the accuracy of diagnosis and prognosis assessment. Future research should focus on molecular modules composed of miR-7 and its key regulatory factors (such as ciRS-7, DNA methylation) and target genes (such as EGFR, α-synuclein, UBE2A), rather than relying on a single marker. For example, in glioma, the combined detection of miR-7 methylation status, ciRS-7 expression levels, and EGFR protein expression can form a comprehensive molecular scoring system to more accurately predict tumor grade, treatment response, and prognosis [27, 97]. In Alzheimer’s disease, the expression combination of miR-7/ciRS-7/UBE2A may better reflect β-amyloid clearance capacity and disease progression than miR-7 alone [35]. Additionally, integrating multi-omics data (transcriptomics, proteomics, metabolomics) and utilizing machine learning algorithms to construct multi-dimensional predictive models that include miR-7 will further enhance the efficacy of diagnosis and prognosis assessment.

Innovations and standardization in detection technology will provide robust support for the clinical application of miR-7. Novel detection techniques such as digital PCR (dPCR) offer higher sensitivity and absolute quantification capabilities, enabling precise detection of trace amounts of miR-7 in bodily fluids [97]. CRISPR-Cas system-derived detection methods (e.g., SHERLOCK, DETECTR) also demonstrate ultra-high sensitivity and hold promise for rapid point-of-care testing of miR-7. Meanwhile, establishing standardized operating procedures (SOPs) for miRNA detection is crucial, covering all steps from sample collection, RNA extraction, reverse transcription, amplification, to data analysis. International standardization organizations and academic bodies should promote the development of relevant guidelines to ensure comparability of results across different laboratories. For instance, selecting stably expressed reference miRNAs (such as members of the let-7 family) for normalization can reduce detection errors [108].

Finally, enhancing the clinical translation of miR-7 in brain diseases is a key future direction. Large-scale, multicenter prospective cohort studies are needed to validate the diagnostic, prognostic, and therapeutic response prediction value of miR-7 and its regulatory network in various brain diseases (such as glioma, AD, PD, and schizophrenia). For example, in glioma patients, prospective studies can determine the relationship between miR-7 expression levels and postoperative recurrence, sensitivity to radiotherapy and chemotherapy, and survival, thereby establishing a miR-7-based prognostic risk stratification model [27, 97]. In PD, long-term follow-up observations can assess the correlation between miR-7 levels and the progression of motor symptoms and cognitive decline [75, 98, 99]. Additionally, exploring the combined application of miR-7 as a therapeutic target and biomarker, known as the “theranostics” model, will be an important future trend. For instance, by detecting miR-7 levels, patients suitable for miR-7 mimic therapy can be screened, and changes in miR-7 levels can be monitored during treatment to evaluate efficacy and adjust dosages, achieving personalized precision medicine.

Interdisciplinary integration will inject new vitality into miR-7 research. Advances in epigenetics, nanomedicine, systems biology, and other disciplines will provide new ideas and methods for the detection and regulation of miR-7. For example, using nanomaterials to develop efficient EVs isolation and miR-7 detection platforms [27]; employing systems biology approaches to construct mathematical models of miR-7 regulatory networks to predict disease progression and treatment response [109]; combining single-cell sequencing and spatial transcriptomics to analyze the specific roles of miR-7 in different cell types and regions of the brain [1]. These interdisciplinary integrations will greatly advance our understanding of the biological functions of miR-7 and accelerate its clinical translation and application.

In summary, miR-7 holds significant potential as a biomarker for brain diseases, but challenges such as tissue specificity, regulatory complexity, detection standardization, and clinical validation must still be overcome. Through technological innovation, multi-biomarker integration, and in-depth clinical research, miR-7 is expected to become a crucial tool for the early diagnosis, prognosis assessment, and personalized treatment of brain diseases in the future, contributing to improved patient outcomes and quality of life.

## Treatment strategies for brain diseases based on miR-7 and nanomedical applications

### Therapeutic applications of miR-7 mimics and inhibitors

miR-7, as a microRNA highly expressed in the brain, plays a critical role in the pathophysiological processes of various brain disorders, providing a solid theoretical basis for its use as a therapeutic target or therapeutic agent. To address abnormal miR-7 expression levels, current primary therapeutic strategies include the use of miR-7 mimics (agonists) to restore or enhance its insufficient expression in disease states, as well as the use of miR-7 inhibitors (antagonists or antagomirs) to suppress its potential overexpression in specific diseases. These two strategies aim to precisely regulate the biological activity of miR-7, thereby intervening in its downstream target gene networks to achieve therapeutic goals.

In the field of neurodegenerative diseases, the application of miR-7 mimics has been most extensively studied, particularly in Parkinson’s disease (PD). Numerous studies have shown that miR-7 expression is significantly reduced in key brain regions (such as the substantia nigra) of PD patients, and this reduction is closely associated with the abnormal accumulation of α-synuclein (α-Syn) [39, 75, 110]. α-Syn is the main component of Lewy bodies, the pathological hallmark of PD, and its encoding gene SNCA is a direct target of miR-7. Therefore, exogenous supplementation of miR-7 mimics can inhibit the translation of α-Syn by directly binding to the 3’-UTR region of SNCA mRNA, thereby reducing its aggregation and toxicity in neurons [39, 41, 75, 110]. For example, in MPTP-induced PD animal models, stereotactic injection of miR-7 mimics into the striatum significantly reduced α-Syn protein levels, attenuated the loss of dopaminergic neurons, and improved motor dysfunction in the model animals [38]. Further mechanistic studies revealed that miR-7 mimics not only function by inhibiting α-Syn but also suppress neuronal apoptosis by regulating other target genes such as Bax, Sirt2, and Krüppel-like factor 4 (KLF4), alleviating oxidative stress and mitochondrial dysfunction, and inhibiting NLRP3 inflammasome-mediated neuroinflammation [18, 47, 70]. These multi-target regulatory effects endow miR-7 mimics with disease-modifying potential in PD treatment, potentially delaying or even halting disease progression rather than merely alleviating symptoms [39]. In manganese-induced Parkinsonian neurotoxicity models, miR-7 expression is similarly downregulated, and overexpression of miR-7 effectively reverses manganese-induced upregulation of SNCA and FGF-20, suggesting that miR-7 mimics may also have protective effects against environmentally induced PD [110].

In addition to PD, miR-7 mimics have also shown therapeutic potential in AD, although related research is relatively limited. The main pathological features of AD include β-amyloid (Aβ) deposition and hyperphosphorylation of tau protein. Studies have indicated that abnormal expression of an endogenous circular RNA (circRNA)-ciRS-7 -in the brains of AD patients may lead to miR-7 dysfunction. ciRS-7 contains numerous binding sites for miR-7 and acts as a “molecular sponge,” competitively adsorbing miR-7 and thereby inhibiting its activity. In the brains of AD patients, the loss of ciRS-7 leads to elevated levels of free miR-7, which excessively suppresses the expression of its target genes, such as the ubiquitin-conjugating enzyme UBE2A. UBE2A is involved in the ubiquitination and degradation of Aβ, and its reduced expression impairs Aβ clearance, exacerbating AD pathology [34, 35]. Although this mechanism suggests that miR-7 inhibition may be necessary in specific contexts of AD, it also highlights the complexity of the miR-7 regulatory network and its importance in AD pathology. Future approaches may require precise modulation of miR-7 activity based on different stages or specific subtypes of AD, rather than simple enhancement or suppression.

In the field of cerebrovascular diseases, miR-7 mimics have demonstrated neuroprotective effects in both ischemic stroke and intracerebral hemorrhage (ICH) models. Studies have found that transient focal cerebral ischemia leads to sustained downregulation of miR-7 (particularly miR-7a-5p) in rat brains, which alleviates the inhibition of its target gene α-Syn, resulting in α-Syn protein-induced neuronal death [67, 68]. Administration of miR-7 mimics via intracerebroventricular or intravenous injection significantly reduces infarct volume after cerebral ischemia and improves motor and cognitive function recovery in rodent models of different ages and sexes, with minimal peripheral toxicity [67]. Further studies confirmed that the neuroprotective effect of miR-7 depends on its inhibition of α-Syn, as the neuroprotective effect of miR-7 mimics was abolished in α-Syn knockout mice [67, 68]. Additionally, miR-7 knockout (double knockout of miR-7a and miR-7b) mice exhibited more severe brain damage and motor dysfunction after mild focal ischemia, while supplementation with miR-7 mimics restored its neuroprotective effect, further confirming that endogenous miR-7 is essential for neuroprotection after ischemic brain injury [68]. miR-7 may also influence neuronal Ca^2 +^ buffering capacity and energy metabolism by regulating mitochondrial voltage-dependent anion channels (VDACs). Ischemic postconditioning may exert neuroprotective effects by stabilizing VDACs, and miR-7 is considered one of the factors leading to the decreased expression of VDAC1 and VDAC3 after ischemia. Therefore, inhibiting miR-7 may also be a potential therapeutic strategy for ischemic brain injury, suggesting that miR-7 may play different roles in different diseases or stages of disease, requiring fine regulation [42].

For ICH, research has similarly confirmed the therapeutic value of miR-7 mimics. After ICH, intracerebroventricular injection of miR-7 mimics significantly improves neurological function scores in model rats, reduces cerebral edema, and suppresses the inflammatory response in brain tissue surrounding the hematoma. The mechanism primarily involves miR-7‘s inhibition of the epidermal growth factor receptor (EGFR)/signal transducer and activator of transcription 3 (STAT3) signaling pathway, thereby reducing the overactivation of astrocytes (decreased GFAP expression) [28]. Additionally, miR-7 can directly target and inhibit the expression of the NLRP3 inflammasome, reducing the production of pro-inflammatory cytokines (such as TNF-α, IL-1β, IL-6) in microglia/macrophages, further alleviating secondary brain injury after ICH [33].

In brain tumors, particularly glioblastoma (GBM), miR-7 is generally regarded as a tumor-suppressive microRNA, with its expression significantly downregulated in GBM tissues. Therefore, restoring miR-7 expression (i.e., using miR-7 mimics) is a primary therapeutic strategy. miR-7 mimics can inhibit the proliferation, invasion, migration, and epithelial-mesenchymal transition (EMT) of GBM cells, and induce their apoptosis by targeting multiple oncogenes and signaling pathways. For example, miR-7 can directly target T-box transcription factor 2 (TBX2), whose high expression is associated with poor prognosis in GBM. By inhibiting TBX2, miR-7 upregulates E-cadherin and downregulates vimentin, thereby reversing the EMT process and reducing cell invasion and distant metastasis [111]. miR-7 can also target key molecules in the mTOR/PI3K/Akt signaling pathway (such as mTOR, PI3K, and Akt) as well as PTEN, regulating the cell cycle and cell metabolism, and inhibiting the growth of GBM cells [112]. In studies on the inhibition of GBM progression by sevoflurane (a volatile anesthetic), it was found that sevoflurane can downregulate the long non-coding RNA HMMR-AS1 (a ceRNA of miR-7), thereby relieving its sponge adsorption effect on miR-7, restoring miR-7 expression, subsequently inhibiting the expression of its target gene CDK4, and ultimately suppressing the viability, invasion, and colony-forming ability of GBM cells [39]. Additionally, miR-7 can exert antitumor effects by targeting oncogenes such as EGFR, which is consistent with reports in other cancer types [113].

In contrast to miR-7 mimics, the application scenarios for miR-7 inhibitors are relatively limited, primarily targeting situations where excessive miR-7 expression leads to pathological damage. For example, in a trinitrobenzene sulfonic acid (TNBS)-induced inflammatory bowel disease (IBD) mouse model, miR-7 expression was significantly elevated in colonic tissues, while trefoil factor 3 (TFF3) expression was reduced, showing a negative correlation between the two. TFF3 has a protective effect on the intestinal mucosa, and further studies revealed that miR-7 directly targets the 3’-UTR of TFF3. Intraperitoneal injection of miR-7 antagomir (inhibitor) significantly reduced colonic miR-7 levels, upregulated TFF3 expression, improved the disease activity index (DAI) in mice, and alleviated pathological damage to the intestinal mucosa [114]. Although this research was conducted in the context of intestinal diseases, it suggests that in the central nervous system, if similar miR-7 overactivation leads to neural damage, miR-7 inhibitors may have therapeutic potential. Additionally, in Alzheimer’s disease (AD), the loss of ciRS-7 results in a relative surplus of miR-7, theoretically making it possible to use miR-7 inhibitors to neutralize the excess miR-7 and restore normal expression of target genes such as UBE2A [34, 35]. However, direct studies on the application of miR-7 inhibitors in brain diseases are still very scarce, which may be related to the fact that miR-7 is highly expressed in normal brain tissue and generally plays a neuroprotective role. Inhibiting miR-7 requires careful evaluation of its potential side effects.

In summary, miR-7 mimics and inhibitors, as two primary miRNA-targeted therapeutic tools, demonstrate clear therapeutic potential in various brain diseases. miR-7 mimics have become a research hotspot due to their multi-target protective and inhibitory effects in neurodegenerative diseases (PD), cerebrovascular diseases (stroke, ICH), and brain tumors (GBM), while the application of miR-7 inhibitors requires further basic research to identify suitable disease models and therapeutic targets. However, whether for mimics or inhibitors, their clinical translation faces a core challenge—how to efficiently and specifically deliver these RNA molecules to target cells in the brain while minimizing off-target effects and toxic side effects. This is precisely the key issue that nanomedicine needs to address in the therapeutic application of miR-7.

### Advantages and challenges of nanocarriers in miR-7 delivery

miR-7, as a highly promising therapeutic target for brain diseases, faces its greatest clinical application bottleneck in the lack of safe and effective delivery systems. miRNA molecules themselves possess characteristics such as small molecular weight, susceptibility to degradation, negative charge, poor cell membrane penetration ability, and rapid renal clearance in vivo, making direct in vivo application largely ineffective and potentially triggering immune responses [115–118]. The advancement of nanocarrier technology provides powerful tools to address these challenges. Nanocarriers can effectively encapsulate miR-7 mimics or inhibitors, protecting them from nuclease degradation, prolonging their circulation time in the bloodstream, and enabling targeted delivery through modifications, thereby enhancing the enrichment of miR-7 in brain lesion areas and reducing toxic side effects on normal tissues. However, applying nanocarriers for brain miR-7 delivery still faces multiple challenges, including blood-brain barrier (BBB) penetration, cellular internalization, endosomal escape, as well as the biocompatibility and biodegradability of carrier materials.

#### Selection and modification of nanomaterials

There is a wide variety of nanomaterials used for miR-7 delivery, each with its unique physicochemical and biological properties. Currently, the most extensively studied ones mainly include lipid-based nanocarriers, polymer-based nanocarriers, inorganic nanocarriers, and biologically derived nanocarriers (such as extracellular vesicles).

Lipid-based nanocarriers, particularly liposomes and lipid nanoparticles (LNPs), are among the most well-researched nucleic acid delivery systems. They consist of natural or synthetic phospholipid bilayers, exhibiting excellent biocompatibility and biodegradability, and can efficiently encapsulate negatively charged miRNA molecules through electrostatic interactions. For instance, a cationic liposome composed of 1,2-dioleoyl-3-trimethylammonium-propane (DOTAP), 1,2-dioleoyl-sn-glycero-3-phosphoethanolamine (DOPE), and cholesterol has been successfully used to encapsulate miR-7 mimics and achieve effective in vivo delivery in ovarian cancer models [113]. In brain disease research, liposomes have also been explored for delivering miR-7 to brain tumors or ischemic regions. The surface of liposomes can undergo various modifications to enhance delivery efficiency and targeting, such as PEGylation (polyethylene glycol modification) to reduce clearance by the reticuloendothelial system (RES) and prolong circulation time, while the attachment of targeting ligands like transferrin receptor antibodies, insulin receptor antibodies, or peptides (e.g., RVG29 peptide derived from the rabies virus glycoprotein) aids in crossing the blood-brain barrier (BBB) and targeting specific brain cell types [118–120].

Polymer-based nanocarriers have also garnered widespread attention due to their advantages, such as strong structural designability, high drug-loading capacity, and the ability to optimize performance by adjusting monomer composition and molecular weight. Commonly used polymers include polyethyleneimine (PEI), poly (lactic-co-glycolic acid) (PLGA), dendrimers, and chitosan, among others. PEI, rich in amino groups, becomes highly positively charged upon protonation at physiological pH, facilitating endosomal escape through the “proton sponge effect” and enhancing the cytoplasmic release efficiency of miRNA. However, its high charge density also results in significant cytotoxicity, which necessitates toxicity reduction through PEGylation or other modifications [89]. PLGA is an FDA-approved biodegradable polymer with excellent biosafety, often used to prepare nanoparticles or microspheres. By modulating its hydrophilicity and hydrophobicity, the release rate of miR-7 can be controlled [121]. For instance, an mPEG-PLGA-PLL (polyethylene glycol-poly (lactic-co-glycolic acid)-polylysine) triblock copolymer nanoparticle was employed for the co-delivery of paclitaxel (PTX) and miR-7, achieving synergistic antitumor effects between chemotherapeutic and genetic drugs [121]. Graphene and its derivatives, such as graphene oxide (GO), as novel two-dimensional carbon nanomaterials, also demonstrate potential as miRNA delivery carriers. GO possesses an enormous specific surface area and abundant surface functional groups (e.g., hydroxyl, carboxyl, epoxy groups), enabling efficient loading of miR-7 through π-π stacking, electrostatic adsorption, or covalent bonding. In GBM research, GO forms a nano-system via self-assembly with miR-7 mimics, effectively transfecting various GBM cell lines such as U87 and U118, and inhibiting tumor growth in xenograft models [112]. The surface of GO can also be functionalized to enhance biocompatibility and targeting capabilities.

Inorganic nanocarriers such as gold nanoparticles, magnetic nanoparticles, silica nanoparticles, and nanodiamonds (NDs) also hold a place in miR-7 delivery due to their unique physicochemical properties (e.g., optical and magnetic characteristics) and high stability. Nanodiamonds, with their excellent biocompatibility, low toxicity, and surface modifiability, have been used in miR-7 delivery research for Parkinson’s disease (PD). miR-7-loaded nanodiamonds (*N*-7) can be efficiently taken up by dopaminergic neurons, inhibit α-Syn expression, reduce oxidative stress, and restore dopamine levels, thereby exerting neuroprotective effects [122]. Magnetic nanoparticles, on the other hand, enable targeted delivery of miR-7 under the guidance of an external magnetic field, increasing drug concentration at the lesion site. This “magnetofection” technology has been applied in localized brain delivery, such as using Neuromag® magnetic particles to deliver miRNA inhibitors to the rat striatum [123].

Biologically derived nanocarriers, primarily referring to extracellular vesicles (EVs), including exosomes, microvesicles, and others, are nano-scale membranous vesicles naturally secreted by cells. EVs possess inherent biocompatibility, low immunogenicity, and intrinsic cell-targeting capabilities, enabling them to cross the blood-brain barrier (BBB), making them ideal candidates for brain drug delivery systems [124, 125]. Mesenchymal stem cell (MSC)-derived EVs are commonly used as delivery vehicles for miRNAs. Through genetic engineering, MSCs can be made to overexpress specific miR-7, and the secreted EVs, which are enriched with miR-7, can be collected for the treatment of neurodegenerative diseases [125]. For instance, small EVs derived from umbilical cord blood mononuclear cells loaded with miR-124-3p have demonstrated neuroprotective effects in Parkinson’s disease (PD) models [124], and similar strategies can be applied to miR-7. The surface of EVs can also be modified via genetic engineering or chemical methods to introduce targeting ligands, further enhancing their delivery efficiency and specificity.

In addition to the main types mentioned above, several novel intelligent responsive nanocarriers are also under exploration. For instance, near-infrared (NIR) light-responsive semiconducting polymer (SP) nanocarriers can generate photoacoustic radiation force under NIR pulsed laser irradiation, enabling rapid delivery of miR-7 to tumor tissues within 20 minutes—achieving a fivefold increase in efficiency compared to traditional passive targeting [126]. This “light-controlled delivery” strategy offers the potential for spatiotemporally precise release of miR-7.

**Fig. 3:**
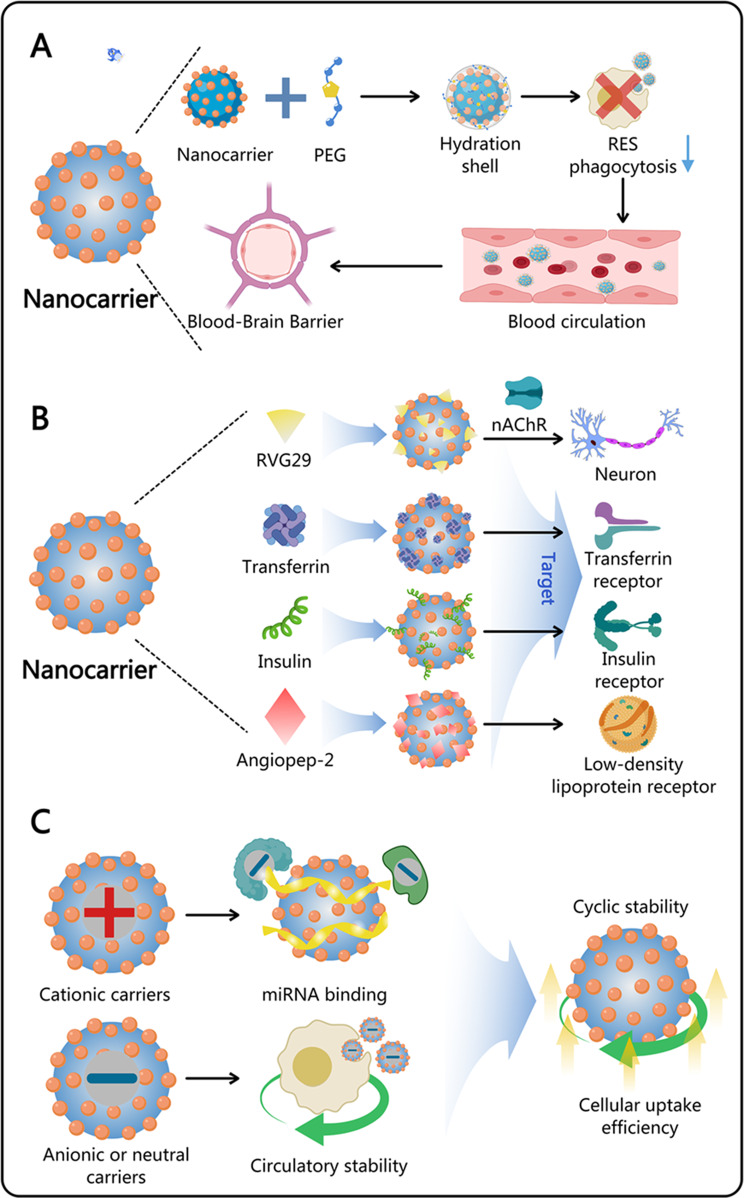
Schematic illustrations of nanocarrier design strategies for brain delivery. (**A**) PEGylated nanocarriers escape RES phagocytosis due to the formation of a hydration shell, leading to prolonged blood circulation. The nanocarrier must subsequently cross the blood–brain barrier (BBB) to reach the brain parenchyma, reduce plasma protein adsorption and opsonization → decrease RES phagocytic clearance, increasing the chance of drugs reaching the BBB. (**B**) Targeting ligands (RVG29, transferrin, insulin, angiopep-2) bind to specific receptors on the BBB endothelium, including nicotinic acetylcholine receptor (nAchr), transferrin receptor, insulin receptor, and low-density lipoprotein receptor, facilitating receptor-mediated transcytosis, enabling precise drug localization to target cells. (**C**) Comparison of carrier surface charge properties: using pH-sensitive materials or charge-reversal polymers, smart carriers can be designed to alter surface charge under different physiological environments (e.g., blood, tumor microenvironment, intracellular endosomes), cationic carriers enable miRNA binding but may compromise circulatory stability, balancing circulation stability and cellular uptake efficiency. This combines the advantages of high endocytosis efficiency of cationic carriers and the excellent circulation stability of anionic carriers. whereas anionic or neutral carriers improve circulatory stability and cellular uptake efficiency

Surface modification of nanomaterials is a key step in enhancing the efficiency of miR-7 delivery to the brain. The most commonly used modification strategies include:

1. PEGylation: This is the gold standard for enhancing the circulatory stability of nanocarriers. PEG chains form a hydration layer on the surface of nanocarriers, reducing plasma protein adsorption and opsonization, thereby decreasing RES phagocytic clearance, prolonging their circulation time in the blood, and increasing the opportunity to reach the BBB (Fig. 3A) [113, 120].
2. Targeted Ligand Modification: To cross the BBB and target specific brain cells (such as neurons, glial cells, and tumor cells), targeting molecules are often conjugated to the surface of nanocarriers. Rabies virus glycoprotein-derived peptides (e.g., RVG29) can specifically recognize nicotinic acetylcholine receptors (nAChR) on the neuronal surface, thereby efficiently mediating brain delivery and neuronal targeting of nanocarriers [119]. Other commonly used targeting ligands include transferrin (targeting transferrin receptors), insulin (targeting insulin receptors), and Angiopep-2 (targeting low-density lipoprotein receptor-related protein 1, LRP1) [117, 119, 127]. For example, DNA nanoflowers (DFs) modified with RVG29 peptide can simultaneously achieve BBB penetration and neuronal targeting, enabling efficient delivery of miR-124 to the hippocampus of AD model mice [127]. This strategy is also applicable for the brain delivery of miR-7 (Fig. 3B).
3. Charge Regulation: The surface charge of nanocarriers is crucial for their interaction with cell membranes, endocytosis efficiency, and blood stability. Cationic carriers typically exhibit strong binding capacity with negatively charged miRNAs and high cellular endocytosis efficiency, but they are also prone to binding with negatively charged plasma proteins, leading to aggregation and clearance (Fig. 3C). Although anionic or neutral carriers demonstrate better circulatory stability, their endocytosis efficiency may be lower (Fig. 3C). Therefore, by utilizing pH-sensitive materials or charge-reversal polymers, smart carriers can be designed to alter their surface charge in different physiological environments (e.g., blood, tumor microenvironment, intracellular endosomes), thereby balancing circulatory stability and cellular uptake efficiency [118].

#### Targeting and safety of nano-delivery systems

**Fig. 4:**
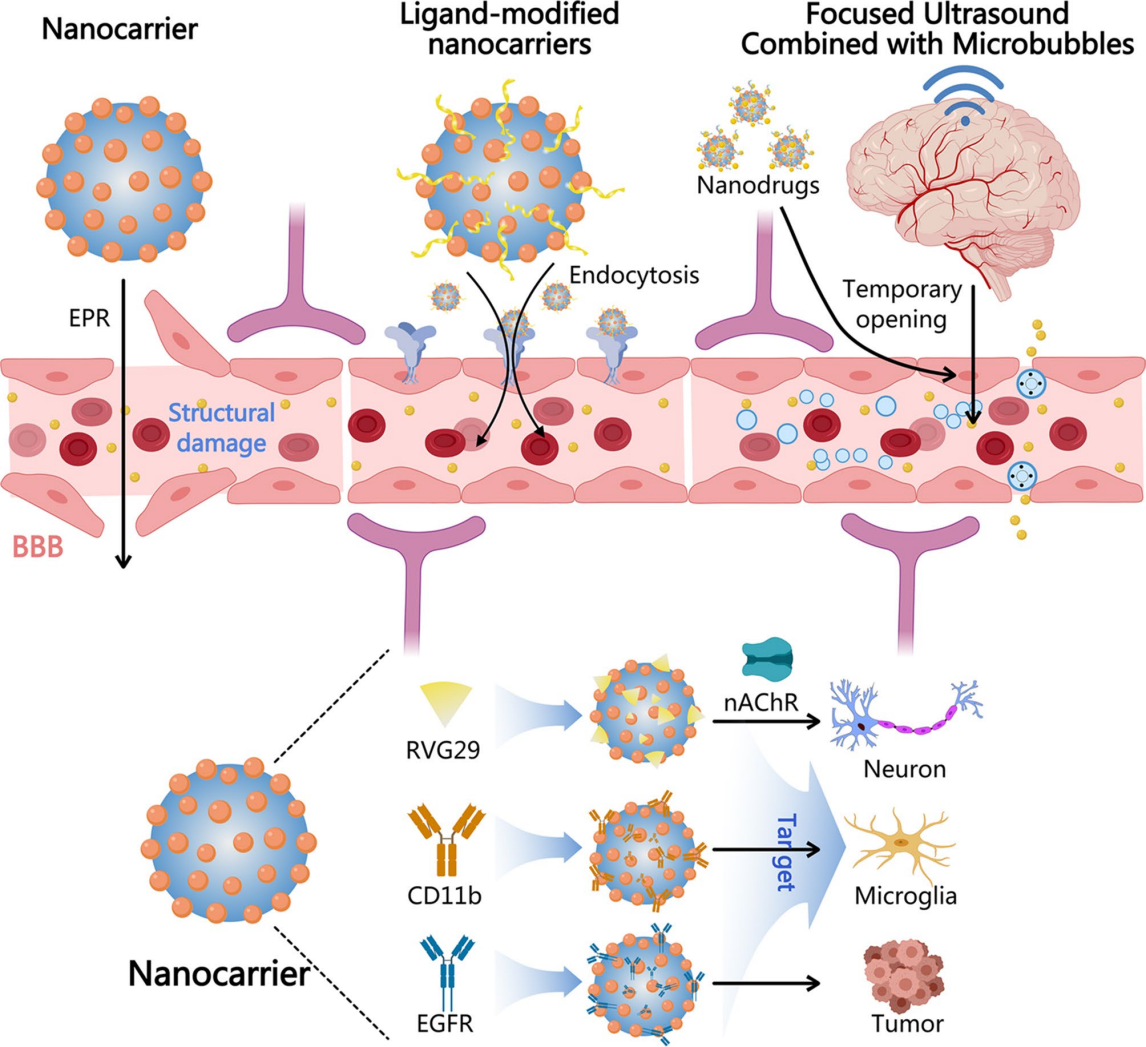
Schematic illustration of combined strategies for enhanced drug delivery across the blood–brain barrier (BBB) and targeting of brain tumors. Ligand-modified nanocarriers (e.g., RVG29 targeting nAchr) facilitate receptor-mediated endocytosis, while focused ultrasound combined with microbubbles induces temporary BBB opening and structural damage to improve passive permeability. Once across the BBB, nanodrugs can target tumor cells (e.g., via EGFR) and modulate microglia (e.g., via CD11b), achieving site-specific therapeutic delivery to the brain lesion

The efficient delivery of miR-7 to specific pathological regions and cell types in the brain is the core prerequisite for achieving its therapeutic effects, which primarily involves two major challenges: crossing the blood-brain barrier (BBB) and achieving cell-specific targeting. The BBB, composed of tightly connected brain microvascular endothelial cells, pericytes, astrocytic end-feet, and the basement membrane, serves as a natural physiological barrier that effectively blocks the entry of the vast majority of foreign substances-including over 98% of small-molecule drugs and nearly all large-molecule drugs into brain tissues [117–119]. Therefore, effectively penetrating the BBB is the primary obstacle for nanocarrier delivery to the brain. Currently, the main mechanisms for nanocarriers to cross the BBB include (Fig. 4 and Table 2):

1. Passive targeting: Leveraging the structural integrity disruption and increased permeability of the BBB under pathological conditions (such as brain tumors, cerebral ischemia, and neuroinflammation), nanocarriers passively leak into the lesion area through the “enhanced permeability and retention effect” (EPR effect). This is one of the primary mechanisms for nanodrug delivery in brain tumors, but it has limited efficacy for chronic neurodegenerative diseases with relatively intact BBB (such as early-stage PD and AD) [112, 117].
2. Active targeting: By modifying the surface of nanocarriers with ligands that can be recognized by specific receptors on BBB endothelial cells, the nanocarriers are transported into the brain through receptor-mediated endocytosis (such as receptor-mediated transcytosis). This is currently the most widely studied and promising BBB penetration strategy. The aforementioned RVG29 peptide, transferrin receptor antibodies, and others are based on this principle [119, 127].
3. Physical/Chemical Methods Assistance: For example, focused ultrasound combined with microbubbles can temporarily open the blood-brain barrier (BBB), creating a window period for nanocarriers to enter specific brain regions; nasal administration can utilize the olfactory or trigeminal nerve pathways to achieve drug delivery to the brain, avoiding systemic circulation and reducing systemic toxicity [118, 120]. For instance, liposomes loaded with miR-128-3p can be non-invasively delivered to the brains of neonatal mice with hypoxic-ischemic brain injury via intranasal administration [118].

**Table 2:** The assembly mechanisms of commonly used nanomaterials targeting miR-7

Materials	Assembly mechanism	Advantages	Challenges
Cationic polymer [121, 128–130]	Electrostatic adsorption compresses miR-7 to form polymeric complexes	Simple synthesis, high loading rate, and enhanced endosomal escape	Cytotoxicity is relatively high (especially for high-molecular-weight PEI), and serum stability may be poor.
Liposomes [113, 131, 132]	Self-assembled formation with a hydrophilic core encapsulating miR-7 and a stable lipid bilayer structure	Good biocompatibility, high transfection efficiency, and easy industrialization	The composition needs precise regulation to balance stability, efficiency and toxicity.
Inorganic nanomaterials [122, 133–136]	Physically adsorbed on the surface/pores, or chemically bonded through chemical bonds	Having controllable morphological dimensions and exhibiting unique optical/magnetic properties	The biodegradability may be poor, and long-term toxicity needs to be assessed.
Exosome/Bionic vesicle [137, 138]	Electroporation, incubation, or engineering maternal cells to encapsulate them	Natural targeting ability, low immunogenicity, strong penetration capability	Large-scale production and standardization are difficult to achieve and drug loading is inconsistent
Hybrid/Smart responsive materials [126]	Combine multiple materials and introduce environment-responsive bonds (such as pH- and GSH-sensitive bonds)	Achieve multi-functional integration and precise control release	The synthesis and characterization are relatively complex.

After successfully penetrating the BBB, nanocarriers must further target specific brain cell types, such as dopaminergic neurons in PD, hippocampal neurons in AD, tumor cells in GBM, surviving neurons in cerebral ischemia, and activated microglia/astrocytes. This is typically achieved by modifying the nanocarrier surface with ligands targeting specific cell surface markers. For example, the RVG29 peptide for targeting neurons, CD11b antibodies for targeting microglia, and EGFR antibodies for targeting tumor cells [119, 122].

Although nanocarriers offer promise for the delivery of miR-7 to the brain, their biosafety issues remain a key factor limiting clinical translation. The toxicity of nanomaterials is closely related to their chemical composition, size, surface charge, shape, as well as the route of administration and dosage.

Cytotoxicity is a primary concern that must be considered. Many nanomaterials, particularly cationic polymers (such as high molecular weight PEI) and unmodified metal nanoparticles, can cause cellular damage through mechanisms including membrane disruption, induction of oxidative stress, interference with mitochondrial function, or activation of apoptosis pathways. For instance, high concentrations of GO may cause physical damage to cell membranes. Therefore, during the selection and optimization of carrier materials, rigorous cytotoxicity assessments (e.g., MTT assay, CCK-8 assay, LDH release assay) must be conducted. Additionally, strategies such as surface modification (e.g., PEGylation), size control, and surface charge modulation should be employed to reduce inherent toxicity [112, 118, 122].

Systemic toxicity is also a barrier that must be overcome for the clinical application of nanocarriers. Intravenously injected nanocarriers distribute to various organs throughout the body and may cause accumulation toxicity in the liver, kidneys, spleen, and other organs. For example, the RES system (primarily macrophages in the liver and spleen) takes up a large number of circulating nanoparticles, which may lead to functional impairment of these organs. Surface modifications such as PEGylation can reduce RES uptake and prolong circulation time, but may also increase accumulation in other organs. Therefore, detailed studies on the in vivo pharmacokinetics and tissue distribution of nanocarriers are necessary, along with an evaluation of their long-term toxicity and immunogenicity [113, 116].

Biodegradability and the safety of metabolites are equally crucial. Ideal nanocarriers should be degradable by the body’s enzyme systems into non-toxic small molecules after completing their delivery tasks and be excreted through normal physiological pathways (such as renal excretion and respiration) to avoid long-term accumulation in the body. Biodegradable polymers like PLGA and polyamino acids, as well as liposomes, have advantages in this regard, while the degradability and long-term safety of some inorganic nanomaterials (such as certain metal oxides) still require further evaluation [115, 121].

Immunogenicity and inflammatory responses are another potential risk. Nanoparticles may be recognized by the body as foreign substances, activating the immune system and triggering immune or even allergic reactions. For example, certain liposomes or virus-like nanoparticles may activate the complement system. Although biologically derived carriers such as extracellular vesicles have lower immunogenicity, they may still carry harmful factors from donor cells. Therefore, a comprehensive evaluation of the immunogenicity of nanocarriers is necessary, including their effects on complement activation, cytokine release, and antibody production [124, 125].

To enhance the safety of nano-delivery systems, researchers are focusing on developing “smart-responsive” nanocarriers. These carriers remain stable under normal physiological conditions but undergo structural changes and release miR-7 specifically in response to microenvironmental stimuli at disease sites—such as reduced pH, elevated glutathione levels, the presence of specific enzymes, temperature variations, or external physical triggers like light or magnetic fields—thereby minimizing exposure and toxicity to healthy tissues [118, 126]. For instance, pH-sensitive polymeric nanocarriers protonate or hydrolyze in the weakly acidic tumor microenvironment or within acidic endosomal compartments, facilitating carrier disassembly and miR-7 release [118]. Light-responsive nanocarriers (e.g., the aforementioned semiconductor polymer nanoparticles) enable precise spatiotemporal targeting and therapeutic accuracy by allowing controlled miR-7 release at specific times and locations via external NIR laser irradiation [126].

In summary, the selection and modification of nanomaterials directly determine the performance of the miR-7 delivery system, with targeting ability and safety serving as the core evaluation criteria for its successful application in the clinical treatment of brain diseases. Although significant progress has been made, the development of a truly efficient, safe, highly targeted, and scalable miR-7 nano-delivery system still requires continuous efforts and innovation across multidisciplinary fields such as materials science, medicine, and biology (Table 2).

### Research examples of miR-7-based nanomedicines in the treatment of brain diseases

With the deepening understanding of miR-7‘s mechanism of action in various brain diseases and the rapid development of nanocarrier technology, miR-7-based nanomedicines have been actively explored for therapeutic applications in multiple animal models of brain disorders, yielding encouraging research outcomes. These examples not only validate the effectiveness of miR-7 as a therapeutic target but also demonstrate the great potential of nanomedicine in overcoming the delivery bottlenecks of miRNAs. Depending on the type of disease, the design strategies, delivery routes, and therapeutic effects of nanomedicines vary in focus.

#### miR-7 nano-delivery to neurodegenerative disease models

In the field of neurodegenerative diseases, PD is the most concentrated area of research for miR-7 nanomedicine. As mentioned earlier, one of the core pathological features of PD is the abnormal aggregation of α-Syn, and miR-7 is an important negative regulator of α-Syn. Therefore, delivering miR-7 mimics via nanocarriers to inhibit α-Syn has become a primary therapeutic strategy. Nanodiamonds (NDs) were selected as the delivery vehicle for miR-7 due to their excellent biocompatibility and low toxicity. In one study, researchers prepared a nanodiamond complex loaded with miR-7 (*N*-7) and evaluated its protective effects on dopaminergic neurons both in vitro and in vivo. The results showed that *N*-7 could be efficiently taken up by dopaminergic neurons and significantly inhibit the expression of α-Syn. In cellular models, *N*-7 alleviated α-Syn-induced oxidative stress damage and restored mitochondrial function. In in vivo PD models (such as those induced by 6-OHDA or MPTP), intracerebral injection of *N*-7 effectively reduced the loss of dopaminergic neurons in the substantia nigra pars compacta, increased the levels of striatal dopamine and its metabolites, and significantly improved the motor coordination and behavioral deficits of the model animals [122]. This study confirmed the feasibility and effectiveness of nanodiamonds as a delivery vehicle for miR-7 in PD treatment.

In addition to nanodiamonds, liposomes and polymeric nanoparticles have also been utilized for miR-7 delivery in PD. For instance, PEG-PLGA nanoparticles were employed to encapsulate miR-7 mimics, with their surfaces modified with RVG29 peptides to enhance BBB penetration and neuronal targeting. Upon tail vein injection, these modified nanoparticles accumulated more effectively in the substantia nigra and striatum regions, where they were endocytosed by dopaminergic neurons and released miR-7. Compared to unmodified nanoparticles, RVG29-modified miR-7 nanomedicines demonstrated superior effects in reducing α-synuclein levels, inhibiting microglial activation (e.g., reducing Iba-1-positive cells and the release of pro-inflammatory cytokines such as IL-1β and TNF-α), and improving motor symptoms in PD model animals [47, 117]. Furthermore, studies have explored the use of mesenchymal stem cell (MSC)-derived extracellular vesicles (EVs) as a natural delivery system for miR-7. By genetically engineering MSCs to overexpress miR-7, the secreted EVs, which are enriched with miR-7, were collected. When administered intranasally or intravenously to PD model animals, these EVs leveraged their inherent properties to cross the BBB, target damaged dopaminergic neurons, and release miR-7 to exert neuroprotective effects. This approach offers advantages such as low immunogenicity and high biocompatibility [125].

In the AD model, although there is relatively little research on nanomedicines directly targeting miR-7, intervention strategies based on the miR-7 regulatory network have shown potential. As mentioned earlier, the loss of ciRS-7 in the AD brain may lead to miR-7 hyperactivity, which in turn suppresses UBE2A expression and affects Aβ clearance. Therefore, theoretically, nanomedicines capable of delivering ciRS-7 mimics or miR-7 inhibitors can be designed to correct this imbalance. A DNA nanoflower (DFs)-based delivery system has been used for the co-delivery of miR-124 and rutin to treat AD, achieving efficient BBB penetration and neuron targeting through RVG29 peptide modification [127]. Similar strategies could be adopted to deliver miR-7 inhibitors or ciRS-7 mimics to the hippocampal and cortical regions of AD model animals to restore UBE2A expression and function and promote Aβ degradation. Furthermore, considering that miR-7 may have a dual role in AD, future developments may include smart responsive nanomedicines capable of dynamically releasing miR-7 mimics or inhibitors based on miR-7 expression levels in different stages of AD or different brain regions.

#### Delivery of miR-7 nanocarriers to brain tumor models

Glioma (particularly GBM) is the most common malignant brain tumor, characterized by high invasiveness and resistance to radiotherapy and chemotherapy, resulting in an extremely poor prognosis. miR-7 is often downregulated in GBM, and its role as a tumor-suppressive miRNA makes it an ideal target for GBM gene therapy, while nanocarriers ensure the effective delivery of miR-7 in GBM.

**Fig. 5:**
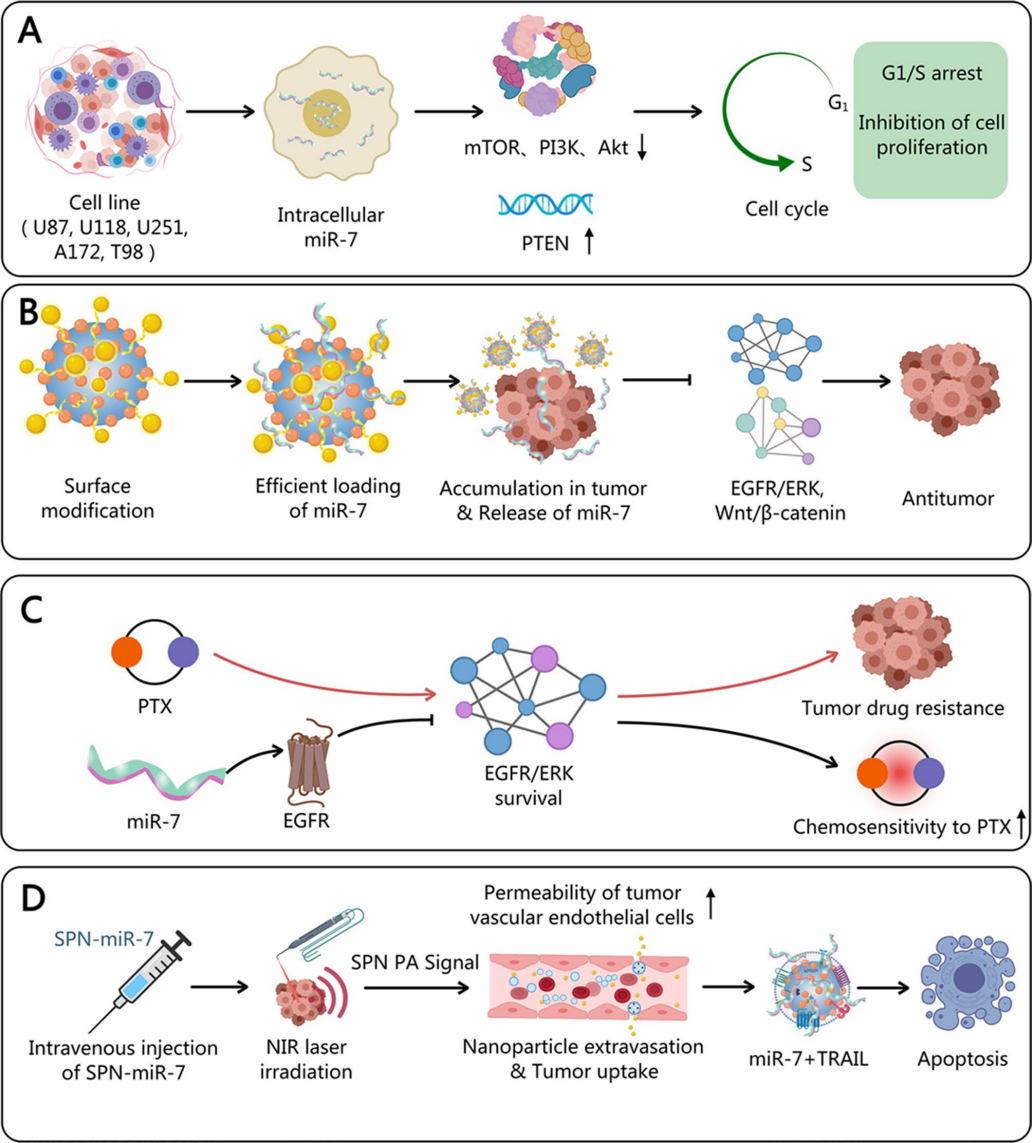
Multi-panel illustration of miR-7-based nanotherapeutics for glioma treatment. (**A**) In glioma cell lines (U87, U118, U251, A172, T98), intracellular miR-7 modulates key signaling pathways (mTOR, PI3K, akt) and upregulates PTEN, leading to G1/S cell cycle arrest and inhibition of cell proliferation. (**B**) Gold nanoparticles: surface-modified with polyethylene glycol (PEG) and targeting peptides, efficiently loaded with miR-7 mimics. miR-7 inhibits EGFR/ERK, Wnt/β-catenin, and other signaling pathways, thereby exerting anti-tumor effects. Surface-modified nanocarriers enable efficient loading of miR-7, which accumulates in the tumor and releases miR-7. This suppresses EGFR/ERK and Wnt/β-catenin signaling, contributing to antitumor effects. (**C**) mPEG-PLGA-PLL triblock copolymer nanoparticles: miR-7 directly targets EGFR, inhibiting the EGFR/ERK survival signaling pathway with paclitaxel (PTX) overcomes EGFR-mediated survival and tumor drug resistance, thereby enhancing chemosensitivity to PTX. (**D**) Semiconducting polymer nanocarriers: following intravenous injection of SPN-miR-7 complexes, near-infrared (NIR) laser irradiation (20 minutes) at the tumor site enhances the delivery efficiency of miR-7 within the tumor. Intravenous injection of SPN-miR-7 (semiconducting polymer nanoparticle-miR-7) followed by NIR laser irradiation increases the permeability of tumor vascular endothelial cells, promoting nanoparticle extravasation and tumor uptake, as detected by SPN photoacoustic (PA) signal. Subsequently, miR-7 acts as a sensitizer for tumor necrosis factor-related apoptosis-inducing ligand (TRAIL), synergizing with TRAIL-inducing compounds (TICs) to sustainably upregulate TRAIL expression, thereby effectively inducing apoptosis in GBM cells and significantly inhibiting tumor growth

Graphene oxide (GO) demonstrates advantages in miR-7 delivery for glioblastoma (GBM) due to its unique two-dimensional structure and high loading capacity. Researchers developed a GO-miR-7 mimic nanosystem via a self-assembly method, which achieves efficient loading of miR-7 through electrostatic interactions and π-π stacking between GO and miRNA. In in vitro experiments, the GO-miR-7 nanosystem effectively transfected various GBM cell lines, including U87, U118, U251, A172, and T98. Following transfection, miR-7 levels in GBM cells significantly increased, while the mRNA and protein expression levels of its downstream target genes, such as mTOR, PI3K, and Akt, were markedly reduced, and the expression of the tumor suppressor gene PTEN was upregulated. These molecular changes led to GBM cell cycle arrest at the G0/G1 phase, significant inhibition of cell proliferation, increased apoptosis rates, and reduced cell migration and invasion capabilities due to alterations in epithelial-mesenchymal transition (EMT)-related markers (Fig. 5A) [112]. In U87 and A172 GBM xenograft mouse models, the GO-miR-7 nanosystem, administered via intratumoral injection or systemic delivery (requiring surface modification to cross the blood-brain barrier), effectively inhibited tumor growth, reduced tumor volume, and prolonged the survival of tumor-bearing mice [112].

Apart from GO, other inorganic nanomaterials such as gold nanoparticles have also been attempted for miR-7 delivery in GBM. Gold nanoparticles exhibit excellent biocompatibility and unique optical properties, facilitating tracking and imaging. Through surface modification with polyethylene glycol (PEG) and targeting peptides (such as those targeting EGFR or integrins highly expressed in GBM cells), gold nanoparticles can efficiently load miR-7 mimics and specifically target GBM cells. In animal models, this targeted nanomedicine accumulates in tumor sites, and after releasing miR-7, it exerts anti-tumor effects by inhibiting signaling pathways such as EGFR/ERK and Wnt/β-catenin (Fig. 5B) [126].

Polymer nanoparticles show great potential in the combined therapy of miR-7 for GBM. For instance, an mPEG-PLGA-PLL triblock copolymer nanoparticle was designed for the co-delivery of the chemotherapeutic drug paclitaxel (PTX) and miR-7 mimics. PTX is one of the first-line chemotherapeutic agents for GBM, but its use is often limited by drug resistance. Studies have found that PTX treatment activates the EGFR/ERK survival signaling pathway, which is one of the mechanisms underlying tumor cell resistance. In contrast, miR-7 can directly target EGFR, effectively inhibiting the activation of this pathway and thereby enhancing the chemosensitivity of PTX. This co-delivery system achieves spatiotemporal synergistic release of PTX and miR-7, significantly inhibiting the proliferation and invasion of GBM cells in vitro and effectively suppressing tumor growth in in vivo xenograft models. Its efficacy is markedly superior to that of PTX or miR-7 used alone (Fig. 5C) [121]. Similarly, the use of biodegradable PLGA nanoparticles for the co-delivery of miR-7 and temozolomide (TMZ, another first-line chemotherapeutic drug for GBM) has also been demonstrated to overcome TMZ resistance and improve therapeutic efficacy through synergistic effects.

Semiconducting polymer nanoparticles (SPNs) represent a novel light-controlled miRNA delivery system that demonstrates unique advantages in glioblastoma (GBM) treatment. Researchers constructed a near-infrared (NIR) light-responsive SPN that loads miR-7 mimics through electrostatic interactions. When exposed to NIR pulsed laser irradiation, the SPN generates a photoacoustic radiation force, which can instantaneously increase the permeability of tumor vascular endothelial cells, promoting the extravasation of nanoparticles and their uptake by tumor tissues. In a GBM xenograft model, intravenous injection of the SPN-miR-7 complex followed by NIR laser irradiation (20 minutes) at the tumor site increased the delivery efficiency of miR-7 into the tumor by fivefold. Subsequently, miR-7 acts as a sensitizer for tumor necrosis factor-related apoptosis-inducing ligand (TRAIL), synergizing with TRAIL-inducing compounds (TICs) to continuously upregulate TRAIL expression, thereby effectively inducing apoptosis in GBM cells and significantly inhibiting tumor growth (Fig. 5D) [126]. This light-controlled delivery strategy not only enhances the targeted delivery efficiency of miR-7 but also achieves spatiotemporally precise regulation of treatment, offering new insights for the precision therapy of GBM.

#### miR-7 nano-delivery to cerebrovascular disease models

Cerebrovascular diseases, primarily including ischemic stroke (cerebral infarction) and ICH, impose a heavy burden on society and families due to their high incidence, disability, and mortality rates. The neuroprotective role of miR-7 in cerebral ischemia and ICH has been confirmed, and the application of nanomedicines further enhances the therapeutic efficacy of exogenous miR-7 in these disease models.

In an ischemic stroke model, the nano-delivery of miR-7 mimics significantly improves brain injury and neurological function recovery. Studies show that sustained downregulation of miR-7 after cerebral ischemia leads to increased disinhibited expression of α-Syn, which exacerbates neuronal death by promoting mitochondrial fragmentation, oxidative stress, and abnormal autophagy [67, 68]. Therefore, supplementing miR-7 is an effective therapeutic strategy. Liposomes are one of the commonly used carriers for miR-7 delivery in cerebral ischemia. For example, miR-7a-5p mimics encapsulated in neutral or mildly cationic liposomes were administered via tail vein injection to rats with middle cerebral artery occlusion (MCAO). The results showed that liposome-encapsulated miR-7a-5p could partially cross the damaged BBB and accumulate in the ischemic penumbra region. Compared to free miR-7a-5p, the liposomal delivery system significantly enhanced the bioavailability of miR-7a-5p in the brain, more effectively inhibited the induced expression of α-Syn, reduced infarct volume, and improved motor and cognitive function recovery in model animals. Its neuroprotective mechanism also involves inhibiting the overactivation of microglia/macrophages and the release of pro-inflammatory cytokines (IL-1β, TNF-α, IL-6) [67]. Further studies found that miR-7 knockout (miR-7-/-) mice exhibited more severe brain injury and motor dysfunction after MCAO, while intravenous injection of miR-7 mimic liposomes restored miR-7 levels and reversed this aggravated damage, confirming the critical protective role of miR-7 in ischemic brain injury [68].

To enhance the delivery efficiency and targeting of miR-7 in ischemic stroke, researchers modified the surface of liposomes. For instance, liposomes modified with transferrin receptor monoclonal antibody (TfR mAb) were used to load miR-7 mimics. TfR is highly expressed on the surfaces of brain microvascular endothelial cells and neurons. Through receptor-mediated transcytosis, TfR mAb-modified liposomes can more effectively cross the blood-brain barrier and target neurons in the ischemic penumbra. In the MCAO model, this targeted liposome-delivered miR-7 mimic demonstrated better neuroprotective effects than unmodified liposomes, significantly reducing infarct volume, alleviating cerebral edema, and promoting long-term neurological recovery (e.g., improved cognitive function as shown in the Morris water maze test) [119].

In addition to liposomes, extracellular vesicle (EV)-based delivery of miR-7 has also shown potential in cerebral ischemia models. For example, EVs isolated from miR-7-overexpressing bone marrow mesenchymal stem cells (BMSCs) are enriched with miR-7. When these EVs were administered to MCAO model rats via tail vein injection, they accumulated in ischemic brain tissue through endogenous homing mechanisms. The released miR-7 not only functions by inhibiting α-Syn but also targets other genes related to ischemic injury, such as the pro-apoptotic Bax and anti-apoptotic Bcl-2, thereby inhibiting neuronal apoptosis and promoting angiogenesis and neuroregeneration [125].

In the ICH model, the nano-delivery of miR-7 mimics primarily exerts neuroprotective effects by inhibiting inflammatory responses and alleviating cerebral edema. Following ICH, intense inflammatory reactions and oxidative stress occur in the perihematomal tissue, activating multiple cell death pathways and leading to secondary brain injury. Studies have found that miR-7 expression is reduced in the brains of ICH model rats, and exogenous supplementation of miR-7 can mitigate these pathological processes by inhibiting the NLRP3 inflammasome and the EGFR/STAT3 pathway [28, 33]. To enhance the delivery efficiency of miR-7 in the ICH model, researchers employed stereotactic injection combined with nanocarriers. For instance, polyethyleneimine (PEI)-modified gold nanoparticles loaded with miR-7 mimics were stereotactically injected into the perihematomal region of ICH model rats using a microinjector. The proton sponge effect of PEI facilitates the cellular uptake of nanoparticles and the release of miR-7. Results showed that this localized delivery method significantly increased miR-7 levels in the perihematomal tissue, effectively suppressed the expression of NLRP3, ASC, and caspase-1, as well as the maturation and release of IL-1β, and reduced the infiltration and activation of microglia/macrophages. Concurrently, miR-7 inhibited the EGFR/STAT3 pathway, attenuating astrocyte hyperproliferation and glial scar formation, thereby alleviating cerebral edema and improving neurological deficit scores [28, 33].

Additionally, studies have explored non-invasive nanodelivery strategies for miR-7 therapy in ICH. For instance, focused ultrasound (FUS) combined with microbubble technology is used to temporarily open the BBB, while miR-7-loaded PEGylated liposomes are administered intravenously. FUS-induced microbubble vibration reversibly disrupts the tight junctions of the BBB, allowing liposomes to enter brain tissue more efficiently. This approach achieves non-invasive, localized, and controllable delivery of miR-7, demonstrating effects in reducing neuroinflammation and improving neurological function in ICH models, while also minimizing the toxic side effects associated with systemic administration [119].

In summary, nano-drugs based on miR-7 have demonstrated significant therapeutic effects in animal models of neurodegenerative diseases (primarily PD), brain tumors (primarily GBM), and cerebrovascular diseases (ischemic stroke and ICH). These examples not only validate the effectiveness of miR-7 as a therapeutic target but also fully demonstrate the critical role of nanocarrier technology in overcoming miRNA delivery barriers and enhancing therapeutic outcomes. Different types of nanocarriers (liposomes, polymers, inorganic nanomaterials, and biologically derived carriers) each have their advantages. Through rational design and surface modification, efficient, targeted, and safe delivery of miR-7 can be achieved, offering new hope for the treatment of these refractory brain diseases.

### Bioengineering miR-7 and combined therapeutic strategies

With the development of gene editing technology and synthetic biology, bioengineered miR-7 and miR-7-based combination therapy strategies have become important research directions for enhancing the therapeutic efficacy of brain diseases and overcoming the limitations of single treatments. Bioengineered miR-7 aims to improve its stability, targeting specificity, or biological activity by optimizing its sequence, structure, or expression regulatory elements. Combination therapy involves integrating miR-7 with other treatment methods (such as small molecule drugs, chemotherapy drugs, other nucleic acid drugs, cell therapy, or phototherapy) to achieve a “1+1 > 2” therapeutic effect through synergistic interactions.

The design and application of bioengineered miR-7 primarily revolve around the following aspects:

1. miR-7 Analogs with Enhanced Stability: Natural miRNAs are highly susceptible to degradation by nucleases in vivo and have a short half-life. By chemically modifying the ribose of miR-7 (e.g., 2’-O-methylation, 2’-fluoro modification, locked nucleic acid (LNA) modification, etc.), its resistance to nucleases can be significantly improved, extending its circulation time and duration of action in the body. For example, LNA-modified miR-7 mimics exhibit higher thermodynamic stability and target gene binding affinity, achieving similar or even better inhibition of α-Syn or other target genes at lower doses compared to unmodified miR-7 [115, 118]. These chemically modified miR-7 analogs are often used in combination with nanocarriers to further enhance their delivery efficiency and targeting specificity.
2. Targeted enhancement of miR-7: Utilizing bioinformatics and genetic engineering techniques, fine-tune the seed sequence or other target recognition regions of miR-7 to enhance its binding specificity to specific target genes and reduce the “off-target effects” on non-target genes. For example, in PD, miR-7 variants with higher affinity and specificity for SNCA mRNA can be designed to more precisely inhibit the expression of α-Syn while reducing the regulation of other potentially beneficial target genes [39, 110]. Additionally, fusion-type miR-7 can be constructed by fusing the miR-7 sequence with coding sequences of cell-penetrating peptides (such as TAT peptide) or nuclear localization signal peptides, enabling intracellular autonomous synthesis and targeted delivery of miR-7 through viral vectors or plasmids.
3. Conditional expression of miR-7: Utilizing disease-specific promoters (such as promoters induced by α-Syn overexpression or oxidative stress in PD, or promoters activated by hypoxia or specific oncogenes in brain tumors) to drive the expression of miR-7, constructing gene therapy vectors (e.g., recombinant adeno-associated virus rAAV). This conditional expression system enables miR-7 to be specifically and highly expressed only in diseased cells or tissues, thereby improving treatment precision and reducing potential toxicity to normal tissues. For example, in GBM cells, hypoxia-inducible factor (HIF-1α) is highly expressed in the tumor microenvironment. Using an rAAV vector with an HIF-1α-responsive promoter to drive miR-7 expression allows for the specific release of miR-7 in hypoxic regions of GBM, exerting an anti-tumor effect [51].
4. Multi-target synergistic miRNA clusters: Construct “miRNA cluster” gene therapy vectors by tandemly expressing miR-7 with other miRNAs that exhibit synergistic effects in the same brain disorder (e.g., miR-124 and miR-132 in PD, miR-124 and miR-21 inhibitors in GBM). This strategy enables simultaneous regulation of multiple disease-related signaling pathways, thereby enhancing therapeutic efficacy. For instance, in PD models, both miR-7 and miR-124 can suppress α-Syn expression and exert neuroprotective effects, and their tandem expression may yield superior outcomes compared to individual miRNA application [98].

Combination therapy strategies based on miR-7 are currently a research hotspot. By combining with treatment methods of different mechanisms, they can effectively overcome the limitations of single miR-7 therapy (such as insufficient efficacy and susceptibility to drug resistance) and broaden its application scope.

1. Combined therapy of miR-7 with small molecule drugs: Neurodegenerative diseases: In PD, combining miR-7 mimics with dopamine precursors (such as levodopa) or dopamine receptor agonists can improve symptoms while delaying disease progression through the disease-modifying effects of miR-7. Co-administration of miR-7 with antioxidants (such as vitamin E, melatonin) or mitochondrial protectants can synergistically reduce oxidative stress and mitochondrial dysfunction, enhancing neuroprotective effects [39, 41]. In AD, the combination of miR-7 (or its regulators such as ciRS-7 mimics) with Aβ clearance agents (such as anti-Aβ antibodies) or tau kinase inhibitors may simultaneously target multiple pathological pathways of AD.
  Brain tumors: The combined application of miR-7 with chemotherapeutic drugs is an important strategy for GBM treatment. As mentioned earlier, the co-delivery system of miR-7 and PTX enhances the chemosensitivity of PTX by inhibiting PTX-induced activation of the EGFR/ERK pathway [121]. Similarly, the combination of miR-7 with TMZ can reverse GBM cell resistance to TMZ by downregulating MGMT (O^6^ -methylguanine-DNA methyltransferase, an enzyme associated with TMZ resistance) or regulating genes related to the DNA damage repair pathway. The combination of miR-7 with radiotherapy can enhance the sensitivity of GBM cells to radiation by inhibiting the expression of DNA damage repair proteins (such as ATM, ATR) [51, 112].
  Cerebrovascular diseases: In ischemic stroke, the combination of miR-7 mimics with antiplatelet drugs (such as aspirin), anticoagulants, or free radical scavengers (such as edaravone) can synergistically exert neuroprotective effects by improving cerebral blood flow, reducing inflammation, and alleviating oxidative stress.
2. Combination therapy of miR-7 with other nucleic acid drugs: miRNA-mRNA combination: Co-delivering miR-7 mimics with genes encoding neurotrophic factors (such as BDNF, GDNF) to inhibit pathological processes while promoting the repair and regeneration of damaged neurons. For example, in PD models, miR-7 suppresses α-Syn toxicity, while BDNF promotes the survival of dopaminergic neurons; their combined use can produce synergistic neuroprotective and neurorestorative effects [75].
  miRNA-miRNA combination: As previously mentioned, the “miRNA cluster” strategy or the combined use of miR-7 mimics with inhibitors of other disease-related miRNAs. For example, in GBM, the combination of miR-7 (tumor-suppressive) and miR-21 inhibitors (miR-21 is an oncogenic miRNA) can synergistically inhibit tumor growth through different signaling pathways [112].
  miRNA-siRNA combination: For certain key target genes (such as EGFR, mTOR) that are difficult to be fully suppressed by a single miRNA, miR-7 can be co-delivered with siRNA targeting that gene. Through different mechanisms (miRNA-mediated translational inhibition and siRNA-mediated mRNA degradation), they synergistically silence the target gene, enhancing the suppression effect [126].
3. miR-7 combined with cell therapy: Stem cell-miRNA combination: Transplanting miR-7-modified neural stem cells (NSCs) or mesenchymal stem cells (MSCs) into brain lesion areas. Stem cells can not only differentiate into functional neurons or glial cells to replenish damaged cells, but their secreted cytokines can also create a favorable microenvironment. At the same time, the highly expressed miR-7 within stem cells can be transferred to surrounding diseased cells through paracrine extracellular vesicles (EVs) or direct cell contact, exerting therapeutic effects. For example, in PD models, transplantation of MSCs overexpressing miR-7 not only differentiates into dopaminergic neuron-like cells but also releases miR-7-enriched EVs that inhibit α-Syn aggregation in host substantia nigra neurons [125].
  Immune cell-miRNA combination: In brain tumor immunotherapy, dendritic cells (DCs) or CAR-T cells loaded with miR-7 are reinfused into the body. miR-7 can regulate the activity and function of immune cells (such as enhancing the antigen-presenting ability of DCs and reducing T cell exhaustion), while directly acting on tumor cells, achieving a synergy between immunotherapy and gene therapy [51].
4. miR-7 and Nanocarrier-Mediated Multimodal Therapy: Photothermal/Photodynamic-Gene Combined Therapy: miR-7 is combined with nanomaterials possessing photothermal or photodynamic therapeutic effects (such as gold nanoparticles, copper sulfide nanoparticles, porphyrin compounds). Under NIR laser irradiation, the nanomaterials generate thermal effects or reactive oxygen species (ROS) to kill diseased cells, while simultaneously releasing miR-7 to inhibit the survival and proliferation of residual cells, effectively preventing recurrence. For example, in GBM treatment, gold nanoshells loaded with miR-7 produce a photothermal effect under laser irradiation to kill tumor cells, while miR-7 inhibits tumor cell migration and angiogenesis [126].
  Imaging-Therapy Integration (Theranostics): Utilizing nanocarriers with imaging functions (such as magnetic nanoparticles for MRI imaging, quantum dots for fluorescence imaging, and gold nanoparticles for CT imaging) to load miR-7. This “theranostic” nanosystem enables real-time monitoring of the distribution of nanomedicines in the body, their accumulation at lesion sites, and treatment efficacy, providing a basis for adjusting individualized treatment plans [119, 122].
5. Combination of miR-7 with surgical treatment: After surgical resection of brain tumors (such as GBM), miR-7 is continuously released through local injection or implantation of sustained-release gels or biodegradable scaffolds containing miR-7 nanomedicines, aiming to eliminate residual tumor cells post-surgery and inhibit tumor recurrence and invasion. This combined strategy can significantly improve the therapeutic efficacy of GBM and patient survival rates [51, 112].

In the aforementioned combination therapy strategies, nanocarrier technology often plays the role of an “all-in-one platform,” enabling the co-delivery and spatiotemporally coordinated release of multiple therapeutic agents. For instance, liposomes or PLGA nanoparticles can simultaneously encapsulate miR-7 mimics, chemotherapeutic drugs, and fluorescent probes, achieving multifunctional integration including targeted delivery, combination therapy, and real-time imaging monitoring [118, 121].

However, bioengineered miR-7 and combination therapy strategies also face numerous challenges, such as the complexity of multi-gene delivery, increased potential immunogenicity, long-term safety assessment, and more complex pharmacokinetic and pharmacodynamic interactions. In the future, more in-depth basic research and rigorous preclinical trials are needed to optimize the efficacy and safety of these novel therapeutic strategies, laying the foundation for their eventual clinical application.

In summary, advances in bioengineering technology have provided new tools for optimizing the function and precise delivery of miR-7, while combination therapy strategies have significantly enhanced the application prospects of miR-7 in treating brain diseases through multi-mechanism, multi-target synergistic effects. The deep integration of these innovative approaches with nanomedicine holds promise for achieving breakthrough progress in tackling challenging brain disorders such as neurodegenerative diseases, brain tumors, and cerebrovascular diseases (Table 3).

**Table 3:** Nanofication of MiR-7 in different disease models

Application fields	Major disease models	Core roles and design principles of nanocarriers	Representative nanomaterials	Ref.
Cancer therapy	Ovarian Cancer Adrenocortical carcinoma Colorectal cancer Glioma NSCLC Papillary thyroid Cervical carcinoma	1. Protection and stability: preventing degradation. 2. Tumor targeting: through EPR effect or surface modification with targeting ligands. 3. Enhancing cellular uptake and endosomal escape. 4. Combination therapy: co-loading with chemotherapeutic drugs.	PLGA-PEG Cationic liposomes ^EGFR^EDV^TM^ nanocells Magnetic nanoparticles FA/PAMAM nanoparticles Au-Nanocube Monoolein cubosome Integrin-targeted biodegradable polymeric nanoparticles DOX/GO/Fe_3_O_4_ Semiconducting polymer (SP) nanocarrier	[113, 121, 126, 128, 129, 131, 133, 134, 136, 137]
Neurodegenerative diseases	Parkinson’s disease Alzheimer’s disease	1. Crossing the BBB: the biggest challenge, often used to describe BBB-penetrating peptides. 2. Neural cell targeting: targeting neurons or microglia. 3. Long-term sustained release: maintaining effective concentration in the brain.	Glb-loaded nanovectors Nanodiamonds hucMSCs-Exos	[122, 132, 138]
Inflammatory diseases	Systemic lupus erythematosus	1. Inflammatory site targeting: utilizing enhanced vascular permeability at inflamed sites. 2. Immune cell targeting: specific delivery to macrophages/microglia to regulate polarization. 3. Responsive release: triggered release in inflammatory microenvironment (pH, ROS).	SA-PLGA	[130]
Integrated diagnosis and treatment	*Helicobacter pylori* infection	1. Integration of therapeutic and imaging functions. 2. Real-time monitoring: tracking carrier distribution, evaluating targeting efficiency and monitoring therapeutic efficacy.	Metal-based nanoparticles	[135]

## Challenges and future directions in miR-7 research

Although significant progress has been made in the study of miR-7 in brain diseases, and its value as a potential therapeutic target and biomarker is widely recognized, numerous challenges remain in areas such as mechanism elucidation, clinical translation, and technological application. A deeper understanding of these bottlenecks and the exploration of solutions are key to advancing miR-7 from basic research to clinical application. This chapter will systematically analyze the main challenges currently facing miR-7 research and, in combination with cutting-edge interdisciplinary technologies, outline future directions for development.

### The complexity of miR-7‘s mechanism of action and the necessity for In-depth research

The biological function of miR-7 is not achieved through a single target or linear pathway but involves participation in cellular physiological and pathological processes via a complex regulatory network. This complexity is primarily reflected in the systematic nature of its regulatory network and its cell specificity. In-depth analysis of these aspects is a prerequisite for elucidating the role of miR-7 in brain diseases.

#### Systematic analysis of the miR-7 regulatory network

miR-7, as a highly conserved microRNA, regulates target gene expression through the principle of base complementary pairing, and the complexity of its functional network far exceeds initial expectations. At the level of epigenetic regulation, miR-7 not only influences mRNA stability and translation efficiency through the canonical targeting inhibition mechanism but also participates in the fine-tuning of gene expression by forming ceRNA networks with other non-coding RNAs, such as circRNA and lncRNA. Among these, ciRS-7, one of the most extensively studied circRNAs, acts as an endogenous miR-7 “sponge” through approximately 70 miR-7 binding sites within its sequence, significantly inhibiting miR-7 function [21, 80]. In the brains of Alzheimer’s disease (AD) patients, the loss of ubiquitin-conjugating enzyme UBE2A leads to decreased stability of ciRS-7, thereby relieving its inhibition of miR-7. This results in abnormal expression of miR-7 target genes (such as UBE2A) and exacerbates AD pathogenesis [34]. Similarly, in glioblastoma, high expression of ciRS-7 sequesters miR-7, alleviating its suppression of target genes like EGFR and PI3KCD, and promotes tumor cell proliferation and invasion [36, 139]. These studies suggest that imbalances in the ceRNA network may be a critical factor contributing to miR-7 dysregulation, though the specific regulatory mechanisms in different brain disorders require systematic elucidation.

In addition to the ceRNA mechanism, miR-7 also influences cell fate decisions by participating in the cross-regulation of multiple signaling pathways. For example, in Parkinson’s disease (PD), miR-7 not only inhibits the expression of α-synuclein (SNCA) by targeting its 3‘UTR [5, 41], but also alleviates neuroinflammation by suppressing NLRP3 inflammasome activation [33], while regulating mitochondrial permeability transition pore (mPTP) function by targeting VDAC1 to improve mitochondrial homeostasis [40]. This multi-target synergistic effect enables miR-7 to exert neuroprotective effects across multiple dimensions, including protein aggregation, inflammatory responses, and energy metabolism. In cerebrovascular diseases, miR-7 inhibits the TLR4/NF-κB pathway by targeting MMP-14, reducing the release of inflammatory factors (such as TNF-α and IL-6), thereby alleviating secondary brain injury after intracerebral hemorrhage [140]. Meanwhile, miR-7 also regulates angiogenesis by inhibiting the EGFR/ERK pathway, influencing vascular repair processes after cerebral ischemia [141]. These findings suggest that miR-7 may act as a key node in signaling pathways, participating in the occurrence and progression of diseases by integrating multiple upstream and downstream signals.

However, the current understanding of the miR-7 regulatory network remains limited. First, the identification of miR-7 target genes largely relies on bioinformatic predictions and in vitro validation, lacking systematic screening at the in vivo level. For instance, although studies have confirmed that SNCA, EGFR, NLRP3, and others are direct target genes of miR-7, the target gene profile of miR-7 may vary across different brain diseases or different stages of the disease, and such dynamic changes have not yet been fully elucidated. Second, the complexity of the ceRNA network far exceeds current knowledge; besides ciRS-7, other circRNAs (such as circHIPK3, hsa_circ_0000735) and lncRNAs (such as HMMR-AS1, LincIN) have also been reported to participate in brain diseases by regulating miR-7 [36, 102, 104], but the interactions among these RNA molecules and their synergistic effects in diseases still require in-depth investigation. Additionally, the regulatory mechanisms of miR-7 expression itself need further refinement. For example, in gastric cancer, abnormal Dicer1 expression leads to impaired pre-miR-7 processing, resulting in downregulation of miR-7 [142]; whereas in rheumatoid arthritis, the negative regulatory relationship between ciRS-7 and miR-7 is involved in the abnormal activation of fibroblast-like synoviocytes [37]. These studies suggest that miR-7 expression is regulated at multiple levels, including transcription and post-transcriptional processing, but its specific regulatory mechanisms in brain diseases still need further clarification.

#### The specific role of miR-7 in different cell types

The central nervous system is composed of various cell types, including neurons, astrocytes, microglia, and oligodendrocytes, each playing distinct roles in physiological and pathological processes. The expression patterns and functional specificity of miR-7 in different brain cell types represent another important feature of its involvement in the regulation of brain diseases, which is also one of the current research challenges.

In neurons, miR-7 is abundantly expressed and crucial for maintaining neuronal survival and function. Studies show that miR-7 inhibits α-synuclein aggregation by targeting SNCA, protecting dopaminergic neurons from toxic damage [5, 41]; meanwhile, miR-7 promotes autophagy to clear abnormal intracellular proteins, reducing Lewy body formation [41]. In AD models, miR-7 alleviates neurofibrillary tangles and senile plaque formation by inhibiting the processing of β-amyloid precursor protein (APP) and tau protein phosphorylation [1]. These findings suggest that the loss of miR-7 in neurons may directly lead to protein homeostasis imbalance, triggering neurodegenerative diseases.

Microglia, as immune cells of the central nervous system, have an activation state (M1/M2 polarization) closely related to neuroinflammation. Although miR-7 expression in microglia is lower than in neurons, its regulatory role in the inflammatory response cannot be overlooked. In intracerebral hemorrhage models, miR-7 inhibits the activation of the inflammasome in microglia/macrophages by targeting NLRP3, reducing the release of pro-inflammatory cytokines such as IL-1β and IL-6, thereby alleviating neuroinflammation and cerebral edema [33]. Similarly, in Parkinson’s disease, miR-7 suppresses excessive microglial activation by inhibiting the TLR4/TRAF6/NF-κB pathway, mitigating dopaminergic neuronal damage [30, 143]. These studies indicate that miR-7 primarily exerts anti-inflammatory effects in microglia, though whether its specific regulatory mechanisms differ from those in neurons remains to be verified.

Astrocytes, as the most abundant cell type in the central nervous system, are involved in various functions such as blood-brain barrier maintenance, nutrient supply, and neural repair. Recent studies have found that miR-7 also plays a significant role in astrocyte activation. In cerebral ischemia models, the high expression of hypoxia-inducible factor-1α (HIF-1α) may upregulate miR-7 expression in astrocytes, thereby inhibiting VEGF expression and affecting post-ischemic angiogenesis and blood-brain barrier repair [141]. Additionally, in glioblastoma, astrocyte-derived exosomal miR-7 exerts anti-tumor effects by suppressing the EGFR pathway in tumor cells [43]. However, compared to neurons and microglia, research on the functions of miR-7 in astrocytes remains relatively limited, and its specific roles in neurodegenerative and cerebrovascular diseases urgently require further exploration.

Oligodendrocytes are responsible for the formation and maintenance of myelin, and their dysfunction is closely associated with demyelinating diseases such as multiple sclerosis. Current research on miR-7 in oligodendrocytes is limited, but existing evidence suggests that miR-7 may influence oligodendrocyte differentiation and myelination by regulating the PI3K/AKT/mTOR pathway [144]. For instance, in oligodendrocyte precursor cells (OPCs), overexpression of miR-7 may inhibit AKT pathway activity by targeting PIK3R1 (a PI3K regulatory subunit), thereby promoting the differentiation of OPCs into mature oligodendrocytes. Although this hypothesis has not been validated in brain disease models, it indicates that miR-7 may be involved in the regulation of myelin homeostasis, offering new directions for research on related diseases.

It is noteworthy that the functions of miR-7 in different cell types may exhibit synergistic or antagonistic effects. For instance, in Parkinson’s disease (PD), the loss of miR-7 in neurons leads to α-synuclein aggregation, while its deficiency in microglia exacerbates neuroinflammation, both of which collectively promote disease progression [33, 39]. This intercellular synergy makes the regulatory network of miR-7 more complex and poses challenges for its clinical application—targeting miR-7 in a single cell type may not achieve the desired therapeutic effect. Therefore, future research needs to integrate technologies such as single-cell sequencing and spatial transcriptomics to systematically analyze the expression profiles and functional differences of miR-7 in various brain cell types, providing a theoretical basis for precise targeted therapy.

### Bottlenecks in the clinical translation of miR-7

Although significant progress has been made in basic research on miR-7 in brain diseases, its clinical translation still faces many challenges, mainly reflected in the efficiency and safety of delivery systems and the standardized detection of biomarkers. Resolving these bottleneck issues is key to advancing miR-7 from the laboratory to the clinic.

#### Efficiency and safety of the delivery system

miR-7, as an endogenous non-coding RNA, possesses the advantage of multi-target synergistic effects. However, the primary obstacle to its clinical application is the lack of an efficient and safe delivery system. The unique characteristics of brain diseases, such as the presence of the blood-brain barrier and the vulnerability of neurons, impose higher demands on delivery systems: they must not only cross biological barriers but also achieve cell-specific delivery while avoiding immunogenicity and neurotoxicity.

Currently, the delivery systems for miR-7 primarily include viral vectors and non-viral vectors. Viral vectors (such as adeno-associated virus, AAV) offer advantages like high transfection efficiency and sustained expression, but their immunogenicity and potential risk of insertional mutations limit their clinical application [39]. Non-viral vectors (such as lipid nanoparticles (LNP), polymeric nanoparticles, and graphene oxide) have become a research focus due to their high safety profile and scalability for large-scale production. For example, encapsulating miR-7 mimics in lipid nanoparticles and administering them via tail vein injection enables efficient delivery in mouse liver cancer models, significantly inhibiting tumor growth [116]. In brain diseases, intranasal delivery of LNP-encapsulated miR-7 can reach the brain via the olfactory nerve pathway, reducing α-synuclein aggregation and dopaminergic neuron loss in Parkinson’s disease model mice [39]. However, the delivery efficiency of LNPs in the brain remains relatively low, and they are easily cleared by the mononuclear phagocyte system, which affects therapeutic efficacy.

Graphene oxide (GO), as a novel nanomaterial, is utilized for the brain delivery of miR-7 due to its high specific surface area and excellent biocompatibility. Studies have shown that GO-miR-7 nanocomplexes can cross the blood-brain barrier via intravenous injection, achieving efficient delivery of miR-7 in glioblastoma models, significantly inhibiting tumor cell proliferation and downregulating the activity of the EGFR/PI3K/mTOR pathway [112]. However, the long-term biosafety of GO still requires evaluation, as its potential oxidative stress damage and neurotoxicity may limit its clinical application. Additionally, the development of responsive nanocarriers (such as pH-sensitive, temperature-sensitive, or enzyme-sensitive carriers) offers new strategies for improving the targeting and controllability of miR-7 delivery. For example, in cerebral ischemia models, nanocarriers responsive to hypoxic environments enable the specific release of miR-7 in ischemic regions, reducing toxic side effects on normal brain tissue [51]. Nevertheless, the preparation process for such carriers is complex, and their efficacy in large animal models has yet to be validated.

#### Limitations of the delivery system

The delivery of microRNA-based therapeutics to the CNS faces formidable obstacles, chief among them the BBB, poor in vivo stability, and the need for controlled release [145–148]. For miR-7 – a key regulator of α-synuclein, inflammasome pathways, and insulin signaling – overcoming these barriers is essential for treating Parkinson’s disease, Alzheimer’s disease, cerebrovascular disorders and brain tumors [1].

Table 4 provides a comparative summary of currently explored nanocarrier systems for miRNA delivery across the BBB. Conventional liposomes, though widely used, achieve less than 1% of the injected dose in the brain and exhibit uncontrolled burst release, limiting therapeutic efficacy [149, 150]. Graphene oxide-based carriers offer high drug loading but their BBB fate remains poorly characterized, and their poor biodegradability raises concerns about long-term retention and chronic neurotoxicity [151–154]. Adeno-associated virus (AAV) vectors, while stable and efficient in rodents, show substantially lower BBB penetration in humans (often < 5% of rodent levels) and lack on-demand release; moreover, pre-existing immunity can neutralize the vector [155–157].

**Table 4:** Comparison of current nanocarrier systems for CNS delivery of miRNAs

Nanocarrier type	BBB penetration mechanism	Delivery efficiency (brain)	Release kinetics	Biodegradability	Immunogenicity/Toxicity	Ref.
Conventional liposomes	Passive diffusion/surface modification (e.g., transferrin)	<1% ID	Burst release, poorly tunable	Low	Low immunogenicity, but off-target accumulation	[149, 150]
Graphene oxide (GO)	Unknown; likely passive	Unknown (quantitative data lacking)	Not controlled	Poor (persists in tissues)	Chronic retention risk, possible neuroinflammation	[151–154]
AAV vectors	Receptor-mediated (e.g., AAV9)	5–10% in rodents, <2% in humans	Constitutive (no on-demand)	Biodegradable but immunogenic	Moderate to high (neutralizing antibodies)	[155–157]
Exosomes (endogenous)	Natural transcytosis	Up to 10–15% ID in rodents	Sustained, natural turnover	Fully biodegradable	Very low (autologous)	[158–162]
Cell-penetrating peptide (CPP)-modified nanoparticles	Cationic adsorption/macropinocytosis	3–8% ID	Tunable (e.g., pH-sensitive)	Moderate (depends on core)	Low to moderate	[163, 164]
Stimuli-responsive polymers (e.g., ROS/pH)	Receptor-mediated + triggered release	5–12% ID in preclinical models	On-demand (enzyme/redox/pH)	Good (hydrolyzable)	Low	[165–168]

ID, injected dose; ROS, reactive oxygen species

Emerging platforms are addressing these shortcomings. Exosomes–naturally occurring extracellular vesicles – leverage endogenous transcytosis pathways and have achieved brain deliveries of 10–15% ID in rodent models, with excellent biodegradability and minimal immunogenicity [158–162]. However, scale-up and miRNA loading consistency remain challenges. Cell-penetrating peptide (CPP)-modified nanoparticles (e.g., TAT or penetratin conjugates) enhance BBB crossing via adsorptive-mediated transcytosis, though their specificity is lower than receptor-mediated systems [163, 164].

Stimuli-responsive nanocarriers represent a major advance [165–168]. By incorporating chemical linkers sensitive to pH (e.g., in endosomes), redox potential (glutathione), or disease-associated enzymes (e.g., matrix metalloproteinases), these systems achieve on-demand miR-7 release specifically within the brain parenchyma or inflamed lesions. For instance, a dual-responsive polymer that releases miR-7 only after triggering by reactive oxygen species (elevated in neurodegeneration) could simultaneously improve efficacy and reduce peripheral toxicity.

#### Translational gap and future directions

Despite these innovations, no miR-7 nanocarrier has entered clinical trials for CNS disorders. The key hurdles that remain underexplored are: (1) quantitative comparison of BBB penetration using standardized in vivo models; (2) long-term stability and release kinetics in non-human primates; (3) off-target effects of miR-7 in peripheral organs; and (4) scalable manufacturing under good manufacturing practices (GMP). Future designs should prioritize receptor-mediated transcytosis (e.g., using transferrin, LDLR, or ApoE-derived peptides), stimuli-responsive release, and complete biodegradability to avoid chronic foreign-body reactions. Combining these features-for example, an exosome-mimetic liposome with a redox-sensitive linker and surface-conjugated brain-homing peptide – could represent the next generation of CNS miRNA therapeutics.

In summary, the comparative analysis in Table 4 highlights that no single carrier currently satisfies all requirements for clinical translation of miR-7. However, rationally designed hybrid systems that integrate BBB-targeting, controlled release, and biodegradability have the potential to unlock miR-7-based therapies for Parkinson’s diseases, Alzheimer’s diseases, cerebrovascular diseases and brain tumors.

Current strategies for crossing the BBB mainly include receptor-mediated endocytosis (such as transferrin receptor TfR and insulin receptor IR-targeting peptide modification), transient BBB opening (such as focused ultrasound combined with microbubbles), and intranasal administration. For example, miR-7 encapsulated in liposomes modified with TfR antibodies can be efficiently delivered to the brain through receptor-mediated transcytosis, significantly reducing Aβ deposition in AD models [1]. However, this method may suffer from reduced delivery efficiency due to receptor saturation, and long-term use may trigger immune responses. Focused ultrasound technology temporarily disrupts the BBB through microbubble vibration, enabling localized delivery of miR-7, but its invasiveness and potential risk of neural damage still need to be weighed. Intranasal administration, as a non-invasive method, can deliver miR-7 to the brain via the olfactory mucosa and trigeminal nerve pathways, showing some neuroprotective effects in PD models [39]. However, its low delivery efficiency and significant individual variability limit its clinical application.

Beyond delivery efficiency, safety is another core issue in the clinical translation of miR-7. Non-viral vectors exhibit relatively low immunogenicity but may cause inflammatory responses and cytotoxicity. For instance, lipid nanoparticles can activate the complement system after intravenous injection, triggering allergic reactions [116]. Meanwhile, high concentrations of miR-7 mimics may saturate the RNA-induced silencing complex (RISC), leading to off-target effects that interfere with normal gene expression. Additionally, long-term overexpression of miR-7 may disrupt the balance of endogenous microRNA networks, resulting in unforeseen side effects. For example, in non-small cell lung cancer, abnormally high expression of miR-7 may promote cancer progression by suppressing tumor suppressor genes [144], suggesting that its function may exhibit a “double-edged sword” effect in different diseases or cellular contexts. Therefore, developing delivery systems with cell specificity and spatiotemporal controllability is key to reducing off-target effects of miR-7 and enhancing therapeutic safety.

#### Standardized detection of biomarkers

miR-7, as a potential biomarker for brain diseases, faces another critical bottleneck in clinical translation: the standardization of its detection. Standardized biomarker detection involves multiple steps, including sample collection, processing, preservation, detection methods, and data analysis. Variations in any of these steps can lead to inconsistent results, undermining its clinical utility.

In the selection of sample types, the detection of miR-7 primarily relies on cerebrospinal fluid (CSF) and peripheral blood (such as serum and plasma). As a bodily fluid in direct contact with the central nervous system, CSF is considered the “gold standard” for reflecting pathological changes in the brain. In the CSF of PD patients, miR-7 levels are significantly reduced and negatively correlated with disease severity (e.g., UPDRS scores) [39, 75]. However, CSF collection is an invasive procedure (lumbar puncture), resulting in poor patient compliance, making it difficult to use for large-scale screening and long-term monitoring. Peripheral blood, as an easily accessible sample source, shows great potential in miR-7 detection. Studies have found that serum miR-7 levels are significantly elevated in AD patients and are correlated with the Aβ42/40 ratio and tau protein levels [1]. In patients with acute ischemic stroke, plasma miR-7 levels significantly increase within 24 hours of onset, making it a potential biomarker for early diagnosis [140]. However, miR-7 in peripheral blood may be influenced by various factors (e.g., hemolysis, diet, medication), and its stability and specificity still require validation. For instance, in an atrazine-induced PD rat model, miR-7 expression was upregulated in the brain but downregulated in peripheral blood. This tissue-specific discrepancy may lead to inconsistencies between peripheral blood miR-7 detection results and brain pathological changes [75], suggesting that single peripheral blood miR-7 detection may not accurately reflect brain conditions and requires combined diagnosis with other biomarkers.

Standardization of detection methods is the core to ensuring the accuracy of miR-7 detection. Currently, the main methods for detecting miR-7 include real-time quantitative PCR (qRT-PCR), microarray chips, and next-generation sequencing (NGS). qRT-PCR is widely used in the clinical detection of miR-7 due to its high sensitivity, strong specificity, and low cost. For example, TaqMan probe-based qRT-PCR can detect low expression of miR-7 in the CSF of PD patients [39], but the results are susceptible to factors such as RNA extraction efficiency, reverse transcriptase activity, and primer design. Microarray chips can simultaneously detect the expression profiles of multiple microRNAs, making them suitable for the combined analysis of miR-7 and other biomarkers, but their low sensitivity makes it difficult to detect low-abundance miR-7. NGS technology offers advantages such as high throughput and the ability to work without prior sequence knowledge, making it useful for discovering new miR-7 isoforms or mutants, but its high cost and complex data analysis limit its routine clinical application. Therefore, establishing unified miR-7 detection standards (e.g., RNA extraction methods, reference gene selection, PCR cycling parameters, etc.) is a prerequisite for achieving comparability of results across different laboratories.

The standardization of data analysis and interpretation is also a crucial aspect of the application of miR-7 as a biomarker. The expression level of miR-7 typically requires normalization using reference genes (such as U6 and RNU48) to reduce inter-sample variability. However, the choice of reference genes can affect the accuracy of the detection results. For example, in cerebral ischemia models, the expression of U6 may be altered due to ischemic stress, leading to bias in the relative quantification of miR-7 [140]. Therefore, screening for stably expressed reference genes or using spike-in external references for calibration is key to improving the reliability of data analysis. Additionally, the clinical interpretation of miR-7‘s significance requires integrating clinical information such as the patient’s age, sex, and comorbidities to establish a multifactorial diagnostic model. For instance, in glioblastoma, the low expression of miR-7 is closely associated with high expression of EGFR, and their combined detection can enhance the accuracy of prognostic assessment [36, 139]. However, there is currently a lack of large-scale, multicenter clinical studies to validate the diagnostic efficacy of miR-7, and the determination of reference ranges and cutoff values still requires further exploration.

### Future research directions and prospects

Facing the current challenges in miR-7 research, future advancements must rely on interdisciplinary integration, the development of innovative technologies and strategies, and the promotion of miR-7‘s translation from basic research to clinical applications. The following sections outline research directions for miR-7 in brain diseases from four perspectives: interdisciplinary collaboration, novel delivery systems, clinical trial design, and the application of cutting-edge technologies.

#### Interdisciplinary integration (epigenetics, nanomedicine, systems biology, etc.)

The mechanism of miR-7 in brain diseases involves multiple levels, including epigenetic regulation, signal transduction, and metabolic reprogramming, and its in-depth research relies on interdisciplinary integration. The combination of epigenetics and systems biology will provide new perspectives for deciphering the regulatory network of miR-7. For example, using ATAC-seq (Assay for Transposase-Accessible Chromatin with sequencing) and ChIP-seq technologies can identify epigenetic modifications (such as DNA methylation and histone acetylation) in the promoter region of miR-7, revealing the upstream mechanisms of its expression regulation. Combining weighted gene co-expression network analysis (WGCNA) and protein interaction network analysis can construct miR-7-related ceRNA networks and signaling pathway interaction maps, clarifying its core regulatory modules in various brain diseases [37, 52]. Additionally, the integration of metabolomics and miR-7 research holds promise for revealing its role in brain energy metabolism. For instance, miR-7 inhibits glycolysis in tumor cells by targeting key enzymes such as HK2 and PFKFB3 [52], and whether similar mechanisms are involved in brain metabolic abnormalities in neurodegenerative diseases warrants further exploration.

Advances in nanomedicine and materials science will provide technical support for the brain delivery of miR-7. The development of nanocarriers with high targeting specificity and biocompatibility is key to overcoming the blood-brain barrier and improving the delivery efficiency of miR-7. For instance, the use of biomimetic nanocarriers (such as nanoparticles coated with red blood cell membranes) can reduce immunogenicity and prolong circulation time. Combining pH-sensitive or ATP-responsive release mechanisms enables the precise release of miR-7 at disease sites [112, 139]. Additionally, 3D bioprinting technology can construct biomimetic brain microenvironment models to evaluate the efficacy and safety of miR-7 delivery systems, offering a more reliable screening platform for preclinical research.

#### Development of novel delivery systems (e.g., responsive nanocarriers)

In response to the shortcomings of existing delivery systems, the development of novel responsive nanocarriers will become a key focus of future research. Responsive nanocarriers can trigger the release of miR-7 based on the microenvironmental characteristics of the lesion site (such as pH, temperature, enzyme concentration, redox potential, etc.), enabling spatiotemporally controlled delivery. For example, in cerebral ischemia regions, hypoxia and acidic microenvironments can serve as stimuli to trigger pH-sensitive nanocarriers to release miR-7, specifically inhibiting neuroinflammation and apoptosis [52, 140]. In glioblastoma, matrix metalloproteinases (MMPs) highly expressed by tumor cells can degrade peptides on the surface of nanocarriers, releasing miR-7 to target and inhibit tumor growth [36]. Additionally, photothermal-responsive nanocarriers (such as gold nanoparticles) can generate localized thermal effects under near-infrared light irradiation, not only directly killing tumor cells but also triggering the release of miR-7, achieving a synergistic effect of chemotherapy and photothermal therapy [112].

In addition to single-responsive vectors, the development of multi-responsive intelligent vectors will further enhance the precision of delivery. For instance, dual-responsive carriers combining pH sensitivity and ATP sensitivity can release miR-7 in a stepwise manner within tumor cells under low pH and high ATP conditions, reducing off-target effects [139]. Moreover, modifying nanocarriers with cell-penetrating peptides (CPPs) can enhance their ability to cross the blood-brain barrier and cell membranes. For example, TAT peptide-modified liposomes encapsulating miR-7 significantly increase its accumulation in the brain and enhance neuroprotective effects in PD models [39]. However, the non-specific binding of CPPs may lead to toxic side effects, and the development of cell-specific CPPs (such as RVG peptides targeting neurons and CD11b antibodies targeting microglial cells) is a future direction.

#### Clinical trial design and validation

The clinical translation of miR-7 relies on scientifically sound clinical trial design. Early-phase clinical trials (Phase I/II) should focus on the safety and tolerability of the miR-7 delivery system, determining the maximum tolerated dose (MTD) and recommended clinical dose (RCD). In trial design, the choice of administration route (e.g., intravenous injection, intrathecal injection, intranasal delivery) must be considered. For instance, intrathecal injection can directly deliver miR-7 to the cerebrospinal fluid, making it suitable for treating neurodegenerative and cerebrovascular diseases, though its potential toxicity to the central nervous system must be evaluated [39]. Although intravenous injection is convenient, it requires addressing the challenge of blood-brain barrier penetration, which may necessitate combination with BBB-opening techniques such as focused ultrasound [112].

In the selection of biomarkers for clinical trials, a multimodal joint evaluation strategy should be adopted. In addition to detecting the expression level of miR-7, it is also necessary to combine imaging indicators (such as PET-CT, MRI) and clinical scales (such as UPDRS, MMSE) to assess treatment efficacy. For example, in PD clinical trials, 18F-DOPA PET can be used to monitor the recovery of dopaminergic neuron function, and combined analysis with miR-7 levels and α-synuclein content in CSF can comprehensively evaluate the neuroprotective effect of miR-7 [39]. Furthermore, regarding the immunogenicity and long-term safety of miR-7, long-term follow-up observation is required, especially in viral vector delivery systems, where potential insertion mutations and autoimmune reaction risks need to be monitored [39].

Given the characteristics of different brain diseases, clinical trial designs should reflect the principle of individualized treatment. For example, in glioblastoma, the therapeutic efficacy of miR-7 may be related to EGFR mutation status, and personalized dosing regimens should be developed based on patients’ molecular subtypes [36, 139]. In Alzheimer’s disease, miR-7 therapy may need to be combined with Aβ antibodies or tau protein inhibitors to achieve multi-target synergistic treatment. However, the safety of combination therapy and drug interactions still require in-depth investigation to avoid the superposition of side effects caused by the non-specific targeting of miR-7.

#### Application of single-cell sequencing and spatial transcriptomics in miR-7 research

Traditional bulk sequencing techniques are unable to reveal the differential expression of miR-7 across various cell types and subpopulations, whereas the development of single-cell RNA sequencing (scRNA-seq) and spatial transcriptomics technologies provides powerful tools to address this issue. Single-cell sequencing enables the profiling of miR-7 expression at the individual cell level, identifying its specific roles in different cell types such as neurons and glial cells. For instance, using single-cell small RNA sequencing, miR-7 target genes that are specifically highly expressed in dopaminergic neurons of the substantia nigra in Parkinson’s disease models can be identified, elucidating their molecular mechanisms in neuronal death [5, 41]. In glioblastoma, single-cell sequencing can reveal differences in miR-7 expression between tumor stem cells and non-stem cell subpopulations, providing a basis for developing therapeutic strategies targeting tumor stem cells [36, 139].

Spatial transcriptomics technology enables in situ detection of miR-7 expression while preserving tissue spatial structure, revealing its distribution characteristics in different regions of the brain. For example, in brain sections of AD models, spatial transcriptomics can visually demonstrate changes in miR-7 expression in regions such as the hippocampus and cortex, as well as its spatial correlation with Aβ deposition and neurofibrillary tangles [1, 34]. Combined with in situ hybridization (ISH) and immunofluorescence techniques, it can also achieve co-localization analysis of miR-7 and target genes (such as SNCA and NLRP3), providing spatial-level evidence to validate the regulatory relationships of miR-7 [33, 41].

Furthermore, the integration of single-cell epigenomics (such as scATAC-seq) and single-cell proteomics technologies can dissect the regulatory network of miR-7 at both epigenetic and protein levels. For example, scATAC-seq can identify chromatin accessibility changes in the promoter region of miR-7, revealing the epigenetic mechanisms of its expression regulation [34]; single-cell proteomics can detect expression changes of miR-7 target proteins at the single-cell level, validating its functional effects [41]. The integrative analysis of these multi-omics data will help construct a multi-dimensional “gene-epigenetic-protein” regulatory network of miR-7 in brain disorders, providing clues for the discovery of new therapeutic targets.

In summary, although research on miR-7 in brain diseases faces numerous challenges, its value as a potential therapeutic target and biomarker has been widely recognized. Through interdisciplinary integration, the development of innovative delivery systems, optimization of clinical trial designs, and the incorporation of cutting-edge technologies such as single-cell sequencing and spatial transcriptomics, it is expected that the clinical translation of miR-7 will be advanced within the next 5–10 years, opening new avenues for the diagnosis and treatment of brain diseases.

## Conclusions

While our findings highlight the tumor-suppressive functions of miR-7 and suggest its potential as a therapeutic target, several major translational challenges must be acknowledged before any clinical application can be contemplated. First, the pleiotropic nature of miRNAs raises concerns about off-target effects; miR-7 may regulate hundreds of downstream genes beyond the identified targets, and unintended modulation of these genes could lead to unpredictable toxicity. Second, efficient and specific delivery of miRNA mimics or inhibitors to the desired tissues or tumors remains a formidable hurdle. Current delivery vehicles (e.g., lipid nanoparticles, viral vectors) face issues such as rapid renal clearance, endosomal entrapment, and potential immunogenicity, particularly for systemic administration. Third, the long-term safety of modulating miR-7 levels is unknown. Chronic suppression of its physiological targets might lead to adaptive resistance or on-target off-tumor toxicities. Therefore, the current evidence, including our own, remains largely preclinical. While miR-7 holds promise, future work should prioritize improving delivery specificity and conducting rigorous long-term toxicological studies in large animal models. Consequently, the translational implications of our study should be interpreted cautiously. Collectively, this review systematically organizes the mechanistic roles of miR-7 in various brain diseases, its potential as a biomarker, and the current research status of miR-7-based therapeutic strategies and nanomedicine applications. Substantial evidence indicates that miR-7, as an important non-coding RNA molecule, plays a critical role in the pathogenesis and progression of various brain disorders, including neurodegenerative diseases, cerebrovascular diseases, and brain tumors through its complex epigenetic regulatory network. In neurodegenerative diseases such as Parkinson’s disease, miR-7 inhibits the expression and aggregation of α-synuclein (SNCA) by directly targeting its 3’ untranslated region, thereby mitigating damage to dopaminergic neurons [5, 6, 169]. Meanwhile, miR-7 can also suppress neuroinflammatory responses by regulating the activation of the NLRP3 inflammasome. For example, in α-synuclein preformed fibril (PFF)-induced Parkinson’s disease models, miR-7 significantly reduces NLRP3 expression, decreases the release of pro-inflammatory cytokines such as IL-1β, and improves motor dysfunction [7, 34, 47]. Additionally, miR-7 is involved in regulating mitochondrial function. By targeting genes such as voltage-dependent anion channel 1 (VDAC1), it improves mitochondrial structure and function, reduces oxidative stress and apoptosis, as demonstrated in MPP+-induced neurotoxicity models [18, 40].

In the context of cerebrovascular diseases, changes in miR-7 expression are closely related to disease occurrence and prognosis. Studies have found that miR-7 expression is significantly downregulated following cerebral ischemia-reperfusion injury, and exogenous supplementation of miR-7 can reduce neuronal death and blood-brain barrier disruption by inhibiting α-synuclein expression, thereby promoting neurological recovery [20, 67, 68]. In intracerebral hemorrhage models, miR-7 can inhibit the overactivation of astrocytes and inflammatory responses by targeting the EGFR/STAT3 signaling pathway, thus alleviating cerebral edema and neurological deficits [28, 33, 170]. For brain arteriovenous malformations (BAVM), miR-7-5p has been found to be associated with the vascular endothelial growth factor (VEGF) signaling pathway, and its abnormal expression may be involved in the regulation of angiogenesis [171, 172].

In brain tumors, particularly glioblastoma (GBM), miR-7 primarily functions as a tumor suppressor gene. It inhibits tumor cell proliferation, migration, invasion, and the epithelial-mesenchymal transition (EMT) process by targeting multiple oncogenes and signaling pathways, such as EGFR, the PI3K/AKT/mTOR pathway, and TCF7L2 [36, 111, 173, 174]. Additionally, miR-7 can regulate tumor cell autophagy and metabolic reprogramming, for example, by inhibiting autophagy-related genes or key glycolytic enzymes, thereby affecting the energy supply and survival of tumor cells [27, 175]. Clinical studies have shown that miR-7 expression levels are typically reduced in GBM tissues, and its low expression is associated with poor patient prognosis, suggesting that miR-7 may serve as a potential biomarker for GBM diagnosis and prognostic evaluation [83, 173, 176].

As a biomarker, changes in the expression of miR-7 in body fluids (such as blood and cerebrospinal fluid) and extracellular vesicles demonstrate significant diagnostic and prognostic value. For instance, elevated levels of plasma miR-7-5p in ischemic stroke patients are associated with the risk of hemorrhagic transformation [177]; in Parkinson’s disease patients, miR-7 expression is also altered in saliva [178]. Furthermore, miR-7 has been identified as a potential biomarker in plasma-derived extracellular vesicles of individuals with alcohol use disorder (AUD), potentially participating in the pathological processes of AUD by regulating hippocampal target genes [179]. These findings provide new insights for the early diagnosis and monitoring of brain disorders.

Therapeutic strategies based on miR-7, particularly those combined with nanomedicine-based delivery systems, demonstrate broad application prospects. Researchers have successfully achieved efficient in vivo delivery and targeted expression of miR-7 using various nanocarriers or gene delivery systems, such as liposomes, graphene oxide, and adeno-associated viruses (AAV). For instance, miR-7-loaded lipid nanoparticles (LNPs) effectively inhibited tumor growth in GBM models [112, 113]; AAV-mediated miR-7 overexpression significantly alleviated α-synuclein-induced pathological damage in Parkinson’s disease models [6]. Furthermore, the combination of miR-7 with chemotherapeutic drugs (e.g., doxorubicin, temozolomide) or natural compounds (e.g., luteolin, silibinin) enhanced antitumor efficacy through synergistic effects and reduced drug resistance [36, 43, 175]. Bioengineered miR-7 and combination therapeutic strategies based on miR-7, such as pairing with immune checkpoint inhibitors or other tumor-suppressive miRNAs, hold promise for further improving treatment specificity and efficacy.

However, research on miR-7 still faces numerous challenges. Firstly, the mechanism of action of miR-7 is extremely complex, involving a regulatory network that encompasses multiple target genes, signaling pathways, and interactions with other non-coding RNAs. For example, ciRS-7, as an endogenous sponge for miR-7, can competitively bind to miR-7, thereby regulating the expression of its target genes. Such intricate ceRNA networks have been reported in models of cerebral ischemia and Parkinson’s disease [2, 19, 20, 69]. Secondly, the specific roles and regulatory mechanisms of miR-7 in different cell types and disease microenvironments require further in-depth analysis. The application of new technologies such as single-cell sequencing and spatial transcriptomics will help reveal its cell-specific functions [180, 181].

In terms of clinical translation, the efficiency and safety of the miR-7 delivery system remain major bottlenecks. Achieving efficient and targeted delivery of miR-7 to the brain while avoiding off-target effects and immune responses continues to be a key focus of current research. Novel responsive nanocarriers, such as pH-sensitive, photothermally responsive, or magnetically targeted nanoparticles, offer new directions to address this challenge [112, 126, 136]. Additionally, standardized detection methods for biomarkers have yet to be established, and issues such as the stability of miR-7 in various body fluids, as well as the sensitivity and specificity of its detection, urgently need to be resolved to advance its clinical application [179, 182, 183].

Looking ahead, research on miR-7 should increasingly focus on interdisciplinary integration, combining cutting-edge technologies such as epigenetics, systems biology, and nanomedicine to deeply explore its functional networks in brain diseases. For instance, using CRISPR-Cas9 technology to construct miR-7 gene-edited animal models will help more precisely investigate its causal relationships in disease development; developing intelligent, multifunctional nanodelivery systems to achieve controllable release and real-time monitoring of miR-7 will lay the foundation for its clinical translation. Meanwhile, conducting large-scale, multicenter clinical trials to validate the efficacy and safety of miR-7 as a biomarker and therapeutic target is a critical step in advancing miR-7 from basic research to clinical application. Additionally, in-depth studies of key nodes within the miR-7 regulatory network, such as the ciRS-7/miR-7/α-synuclein axis, may provide new targets for developing novel combination therapeutic strategies.

Collectively, miR-7 plays a significant biological role and holds clinical application potential in brain diseases. Although numerous challenges remain, with the continuous advancement of research and technology, miR-7 is expected to become a crucial molecular target for the future diagnosis, prognosis assessment, and treatment of brain diseases, offering new hope for improving patient outcomes and quality of life.

## Data Availability

Data sharing is not applicable to this article as no datasets were generated or analyzed during the current study.

## Abbreviations

3’-UTR: 3’-Untranslated region
AAV: Adeno-associated virus
AD: Alzheimer’s disease
Aβ: Amyloid-beta
APP: Amyloid precursor protein
AUC: Under the curve
BBB: Blood-brain barrier
BDNF: Brain-derived neurotrophic factor
BMSCs: Bone marrow mesenchymal stem cells
BTI: Brain tissue inflammation
CDK4: Cyclin-dependent kinase 4
CeRNA: Competitive endogenous RNA
CircRNA: Circular RNA
CSF: Cerebrospinal fluid
CT: Computed tomography
DA: Dopaminergic neuron
DAI: Disease activity index
DCs: Dendritic cells
DFs: DNA nanoflowers
DOPE: 1,2-dioleoyl-sn-glycero-3-phosphoethanolamine
DOTAP: 1,2-dioleoyl-3-trimethylammonium-propane
dPCR: Digital PCR
DR5: Death receptor 5
ECM: Extracellular matrix
EGFR: Epidermal growth factor receptor
EMT: Epithelial-mesenchymal transition
EVs: Extracellular vesicles
FAM177A: Family with sequence similarity 177 member A
FGF-20: Fibroblast growth factor 20
FLS: Fibroblast-like synoviocytes
FUS: Focused ultrasound
GBM: Glioblastoma
GDNF: Glial cell line-derived neurotrophic factor
GFAP: Glial fibrillary acidic protein
GO: Graphene oxide
GSCs: GBM stem cells
HCC: Hepatocellular carcinoma
HD: Huntington’s disease
HIF-1α: Hypoxia-inducible factor
HUVEC: Human umbilical vein endothelial cells
ICH: Intracerebral hemorrhage
IDE: Insulin-degrading enzyme
IR: Insulin receptor
IRS-2: Insulin receptor substrate 2
KLF4: Kruppel-like factor 4
LNA: Locked nucleic acid
LNPs: Lipid nanoparticles
LPS: Lipopolysaccharide
LRP1: Lipoprotein receptor-related protein 1
LXR: Liver X receptor
MCAO: Middle cerebral artery occlusion
MiR-7: MicroRNA-7
miRNAs: MicroRNAs
MMPs: Matrix metalloproteinases
MMSE: Mini-Mental State Examination
mPEG-PLGA-PLL: Polyethylene glycol-poly(lactic-co-glycolic acid)-polylysine
MPP+: 1-methyl-4-phenylpyridinium
MPTP: 1-methyl-4-phenyl-1,2,3,6-tetrahydropyridine
mPTP: Mitochondrial permeability transition pore
MRI: Magnetic resonance imaging
MSA: Multiple system atrophy
MSC: Mesenchymal stem cell
MVI: Microvascular invasion
nAChRs: Nicotinic acetylcholine receptors
NDs: Nanodiamonds
NGS: Next-generation sequencing
NIR: Near infrared
NSCLC: Non-small cell lung cancer
NSCs: Neural stem cells
OPCs: Oligodendrocyte precursor cells
PD: Parkinson’s disease
PEG-PLGA: Polyethylene glycol-poly(lactic-co-glycolic acid)
PEGylation: Polyethylene glycol modification
PEI: Polyethyleneimine
PET: Positron emission tomography
PFF: Pre-formed fibril
PFS: Progression-free survival
PIK3CD: PI3K catalytic subunit δ
PLGA: Poly(lactic-co-glycolic acid)
Pri-miR-7: Primary transcript of miR-7
PTX: Paclitaxel
qRT-PCR: Quantitative real-time PCR
RA: Rheumatoid arthritis
RD: Retinal detachment
RES: Reticuloendothelial system
RISC: RNA-induced silencing complex
ROS: Reactive oxygen species
SCAs: Spinocerebellar ataxias
SCI: Spinal cord injury
Sirt2: Sirtuin 2
SNCA: α-synuclein
SOPs: Standardized operating procedures
SP: Semiconductor polymer
SPNs: Semiconducting polymer nanoparticles
STAT3: Signal transducer and activator of transcription 3
TBX2: T-box transcription factor 2
TFF3: Trefoil factor 3
TfR mAb: Transferrin receptor monoclonal antibody
TICs: TRAIL-inducing compounds
TLR4: Toll-like receptor 4
TMZ: Temozolomide
TNBS: Trinitrobenzene sulfonic acid
TRAIL: Tumor necrosis factor-related apoptosis-inducing ligand
TYRO3: Tyrosine-protein kinase receptor TYRO3
UPDRS: Unified Parkinson’s Disease Rating Scale
VDAC1: Voltage-dependent anion channel 1
XIAP: X-linked inhibitor of apoptosis protein
